# Use of expressed sequence tags as an alternative approach for the identification of *Taenia solium* metacestode excretion/secretion proteins

**DOI:** 10.1186/1756-0500-6-224

**Published:** 2013-06-06

**Authors:** Bjorn Victor, Pierre Dorny, Kirezi Kanobana, Katja Polman, Johan Lindh, André M Deelder, Magnus Palmblad, Sarah Gabriël

**Affiliations:** 1 Veterinary Helminthology Unit, Department of Biomedical Sciences, Institute of Tropical Medicine, Antwerp, Belgium; 2 Medical Helminthology Unit, Department of Biomedical Sciences, Institute of Tropical Medicine, Antwerp, Belgium; 3 Swedish Institute for Communicable Disease Control, Solna, Sweden; 4 Department of Microbiology, Tumor and Cell Biology, Karolinska Institutet, Stockholm, Sweden; 5 Biomolecular Mass Spectrometry Unit, Department of Parasitology, Leiden University Medical Center, Leiden, The Netherlands

**Keywords:** Expressed sequence tag, Excretion/secretion proteins, *Taenia solium*, Proteomics

## Abstract

**Background:**

*Taenia solium* taeniasis/cysticercosis is a zoonotic helminth infection mainly found in rural regions of Africa, Asia and Latin America. In endemic areas, diagnosis of cysticercosis largely depends on serology, but these methods have their drawbacks and require improvement. This implies better knowledge of the proteins secreted and excreted by the parasite. In a previous study, we used a custom protein database containing protein sequences from related helminths to identify *T. solium* metacestode excretion/secretion proteins. An alternative or complementary approach would be to use expressed sequence tags combined with BLAST and protein mapping to supercontigs of *Echinococcus granulosus*, a closely related cestode. In this study, we evaluate this approach and compare the results to those obtained in the previous study.

**Findings:**

We report 297 proteins organized in 106 protein groups based on homology. Additional classification was done using Gene Ontology information on biological process and molecular function. Of the 106 protein groups, 58 groups were newly identified, while 48 groups confirmed previous findings. Blast2GO analysis revealed that the majority of the proteins were involved in catalytic activities and binding.

**Conclusions:**

In this study, we used translated expressed sequence tags combined with BLAST and mapping strategies to both confirm and complement previous research. Our findings are comparable to recent studies on other helminth genera like *Echinococcus*, *Schistosoma* and *Clonorchis*, indicating similarities between helminth excretion/secretion proteomes.

## Findings

## Introduction

*Taenia solium* taeniasis/cysticercosis is a zoonotic helminth infection mainly found in poor and rural regions of Africa, Asia and Latin America where it has a large impact on public health [1-3]. The adult tapeworm develops in the small intestine of humans (taeniasis). Mature proglottids full of eggs break off from the distal end of the worm and leave the body with the stool. Both humans and pigs can act as intermediate hosts when the infective larval stages (oncospheres) inside the eggs are ingested and liberated in the stomach. The oncospheres then enter the blood flow through the intestinal mucosa. Cysticercosis is caused when oncospheres lodge themselves in the subcutaneous and muscle tissues and the central nervous system, where they develop into metacestode larval stages (cysts). In humans, epilepsy and other neurological symptoms can be provoked by immunological reactions against degenerating cysts that have developed in the central nervous system (neurocysticercosis).

Diagnosis of porcine and human (neuro) cysticercosis largely depends on antigen and/or antibody detection, but these serological methods also have their specific drawbacks [4]. Improving current diagnostic assays automatically implies better knowledge of the proteins secreted and excreted by the metacestodes.

Proteomic experiments involving liquid chromatography and tandem mass spectrometry (LC-MS/MS) typically attempt to match the generated experimental spectra to *in silico* spectra from a (target) protein database. Ideally, this database contains every protein likely to be in the sample, but obtaining such an all-including protein database proves difficult when there is little to no genomic information available, as was the case for *T. solium* until recently [5]. In our previous study, we bypassed this limitation by using a custom database with known proteins from related helminths (*Taenia*, *Echinococcus*, *Schistosoma* and *Trichinella*) as a target database in the LC-MS/MS experiments [6]. We deliberately did not use translated expressed sequence tags (ESTs), because we wanted to investigate to usefulness of a target database made up of protein sequences originating mostly from (closely) related helminths.

The usefulness of ESTs for the identification of helminth proteins has already been described for e.g. *Haemonchus contortus*[7,8] and *Echinococcus granulosus*[9]. In the case of *T. solium*, ESTs from different parasite stages have been made available by different research groups, both published [10,11] and unpublished (Huang J. *et al.*, Analysis of *Taenia solium* and *Taenia saginata* adult gene expression profile, 2009 and Aguilar-Diaz H. *et al.*, *Taenia solium* larva/adult ESTs, 2007). In this study, we use *T. solium* ESTs combined with the Basic Local Alignment Search Tool (BLAST) and protein mapping to supercontigs of *E. granulosus* (a member of the Taeniidae family) to investigate whether we could increase the number of *T. solium* metacestode excretion/secretion protein identifications from the previous study.

## Materials and methods

### Generation of the data set

The *in vitro* production of the *T. solium* metacestode excretion/secretion proteins from Peru and Zambia at 24h and 48h and the generation of line spectra mzXML files have been previously described [6].

### Database design and data analysis

To construct the target database, 30,700 expressed sequence tags were downloaded from the National Center for Biotechnology Information (NCBI) website in April 2012 and a six frame translation was performed using transeq [12]. A *Sus scrofa* database with 1,388 Swiss-Prot sequences (http://www.uniprot.org/) and the common Repository of Adventitious Proteins database (112 protein sequences; http://ftp.thegpm.org/fasta/cRAP/crap.fasta) were also included to assist detection of host proteins and accidental contaminations, respectively. A decoy database with 185,700 reversed sequences was created using decoyfasta. These databases were fused into one final database. Database searching with X!Tandem (2010.10.01.1) [13] and subsequent analyses with PeptideProphet [14,15], iProphet [16] and ProteinProphet [17] were also performed as previously described [6]. All above mentioned tools, except transeq, are included with the Trans-Proteomic Pipeline v4.5 RAPTURE rev 2 [18]. The identified translated ESTs were further filtered to a false discovery rate of < 1% and ESTs with an individual probability of zero were discarded. The remaining ESTs were blasted against the NCBI nonredundant database (E-value < 10 ^−10^) and for each recognized EST, the best matching protein was retained. The resulting proteins were then screened by mapping the proteins to the *E. granulosus* supercontigs using TBLASTN (http://www.sanger.ac.uk/cgi-bin/blast/submitblast/Echinococcus). Identifications with a Score > 200 were considered valid. Identifications with a lower score were manually evaluated and proteins originating from *T. solium* were retained. This step also helped to filter out host contaminations. Finally, proteins were grouped based on homology. All proteins that could not be grouped and were identified by only one EST were also discarded. Finally, Blast2GO was used for Gene Ontology (GO) annotations (biological process, molecular function and cellular component) and the construction of level 2 pie charts [19]. In order to gain more specific information, the largest categories were analyzed to levels 3 and 4.

## Results and discussion

### Identified proteins and gene ontology annotation

In this study, 297 proteins (from 1,787 translated ESTs) were identified and organized in 106 protein groups based on homology (Additional file 1). For simplicity, each protein group is represented by one protein. The groups were further organized by Gene Ontology annotation information on biological process and molecular function. A total of 48 protein groups are labelled with an asterisk, indicating that they were also identified in the previous study (Additional file 2) [6]. For brevity, Table 1 shows only the 58 newly identified protein groups. For a number of proteins/protein groups, no Gene Ontology information was available. Nonetheless, many of them, like the 8 kDa protein family [20], have been extensively studied and used in diagnostic assays.

**Table 1:** **Protein groups (*n *= 58) newly identified in *Taenia solium *metacestode excretion/secretion proteins, organized by Gene Ontology annotation information on biological processes and molecular functions**

**Gene ontology classification**		**Closest organism**	**gi code**	**Proteins**^***a)***^	**ESTs**^***b)***^
	**Protein group**				
**1) No Gene Ontology classification**					
	Major egg antigen	*Clonorchis sinensis*	358336515	1	2
	ES1 protein homolog	Multiple^*c*)^	-	3	5
	Phosphoglyceride transfer protein	*Taenia asiatica*	124782980	1	7
	Alpha-2-macroglobulin-like protein 1	*Clonorchis sinensis*	358333571	1	5
	Aldose 1-epimerase	*Clonorchis sinensis*	358334888	1	2
	SJCHGC02626 protein	*Schistosoma japonicum*	-	3	7
	Hypothetical protein	*Schistosoma mansoni*	256079415	1	4
	TSP1	*Echinococcus multilocularis*	209967595	1	4
	Putative major vault protein	*Echinococcus granulosus*	62178032	1	2
**2) Binding (miscellaneous)**					
	Filamin	Multiple	-	3	7
	Methionyl-tRNA synthetase cytoplasmic	*Clonorchis sinensis*	358255967	1	5
	SJCHGC09631 protein	*Schistosoma* spp.	-	2	2
	four and a half LIM domains protein 3	*Clonorchis sinensis*	358341124	1	7
	Alpha-actinin isoform B	*Taenia asiatica*	124783372	1	3
	Calumenin	*Taenia asiatica*	124784033	1	2
	Calcium-binding protein	*Schistosoma mansoni*	256071353	1	2
	Lysyl oxidase-like	*Schistosoma mansoni*	256072781	1	2
	Porphobilinogen synthase	Multiple	-	3	3
	Phosphoglucomutase-1	*Clonorchis sinensis*	358337844	1	2
	Fibrillar collagen	Multiple	-	9	16
**3) Glycolysis/Metabolic processes (miscellaneous)**					
	Adenylosuccinate synthetase	*Schistosoma mansoni*	387912858	1	4
	Adenylate kinase	Multiple	-	2	4
	UDP-glucose pyrophosphorylase 2	*Schistosoma* spp.	-	2	2
	Hypothetical protein SINV_09109	*Solenopsis invicta*	322793762	1	2
	Aspartate aminotransferase	Multiple	-	3	5
	Lactate dehydrogenase A	*Taenia solium*	318054471	1	6
	SJCHGC05968 protein	Multiple	-	2	2
	Methylthioadenosine phosphorylase	Multiple	-	2	2
	Ornithine aminotransferase	Multiple	-	3	3
	Endoglycoceramidase	Multiple	-	3	3
	Aminoacylase	Multiple	-	2	2
	Glucose-6-phosphate 1-dehydrogenase-like	*Sus scrofa*	350595984	1	2
	Phosphoglycerate mutase	Multiple	-	3	7
**4) (Endo)peptidase activity**					
	Calpain	Multiple	-	5	5
	UDP-glucose 4-epimerase	Multiple	-	2	2
	Dipeptidyl-peptidase	Multiple	-	2	3
	Glutamate carboxypeptidase 2	*Clonorchis sinensis*	358331956	1	3
**5) Endopeptidase inhibitor activity**					
	Kunitz protein 8	Multiple	-	2	3
**6) Cell redox homeostasis/Oxidation-reduction related**					
	Carbonyl reductase	*Schistosoma* spp.	-	3	3
	Methionine sulfoxide reductase	Multiple	-	2	4
	procollagen-lysine, 2-oxoglutarate	Multiple	-	3	6
	5-dioxygenase 3				
**7) Transport**					
	Charged multivesicular body protein	Multiple^c)^	-	3	4
	SJCHGC06082 protein	Multiple	-	2	8
	Glycolipid transfer protein-like protein	*Taenia asiatica*	124782916	1	2
	Gamma-soluble NSF attachment protein	Multiple	-	4	10
	Sodium/glucose cotransporter	Multiple	-	2	7
**8) Motor activity/Cytoskeleton and Microtubule related**					
	Tubulin polymerization-promoting protein	Multiple	-	2	2
	Myophilin	Multiple	-	2	19
**9) Miscellaneous Gene Ontology classification**					
	Translation initiation factor 5A	Multiple	-	2	4
	Ubiquitin-conjugating enzyme	Multiple	-	4	10
	Protein-l-isoaspartate o-methyltransferase	*Schistosoma mansoni*	256081696	1	2
	Protein DJ-1-like	Multiple	-	2	4
	6-phosphogluconolactonase	Multiple	-	2	4
	SJCHGC02435 protein	*Schistosoma japonicum*	56756018	1	5
	Family T2 unassigned peptidase	*Schistosoma mansoni*	256088374	1	4
	3’(2’), 5’-bisphosphate nucleotidase	Multiple	-	2	2
	RAB GDP dissociation inhibitor alpha	Multiple	-	2	3
	Laminin	Multiple	-	2	2

a) The number of proteins in each protein group.

b) The number of expressed sequence tags that were matched to proteins in this protein group.

c) ’Multiple’ indicates that different (helminth) genera have identified proteins in that protein group.

For simplicity, all protein groups are represented by one protein.

Most of the identified protein groups could be categorized in miscellaneous binding activities (e.g. Actin binding, calcium binding and metal ion binding), various metabolic processes, gluconeogenesis (Triosephosphate isomerase, Enolase, Phosphoenolpyruvate carboxykinase and Phosphoglucose isomerase), glycolysis (Glyceraldehyde-3-phosphate dehydrogenase, Phosphoglycerate kinase, Phosphoglycerate mutase and Fructosebisphosphate aldolase) and proteins with (endo) peptidase activity, including cysteine-type (Calpain, UDP-glucose 4-epimerase and Cathepsin), threonine-type (Proteasome subunits) and serine-type endopeptidase activity (Trypsin-like protein). Endopeptidase inhibitors with both serine-type (Kunitz protein 8 and Leukocyte elastase inhibitor) and cysteine-type endopeptidase inhibitor activity (Immunogenic protein Ts11) and components of the enzymatic antioxidant system of Taeniidae (Cu/Zn Superoxide dismutase, Glutathione S-transferase and Peroxiredoxin) were also identified [21].

**Figure 1:**
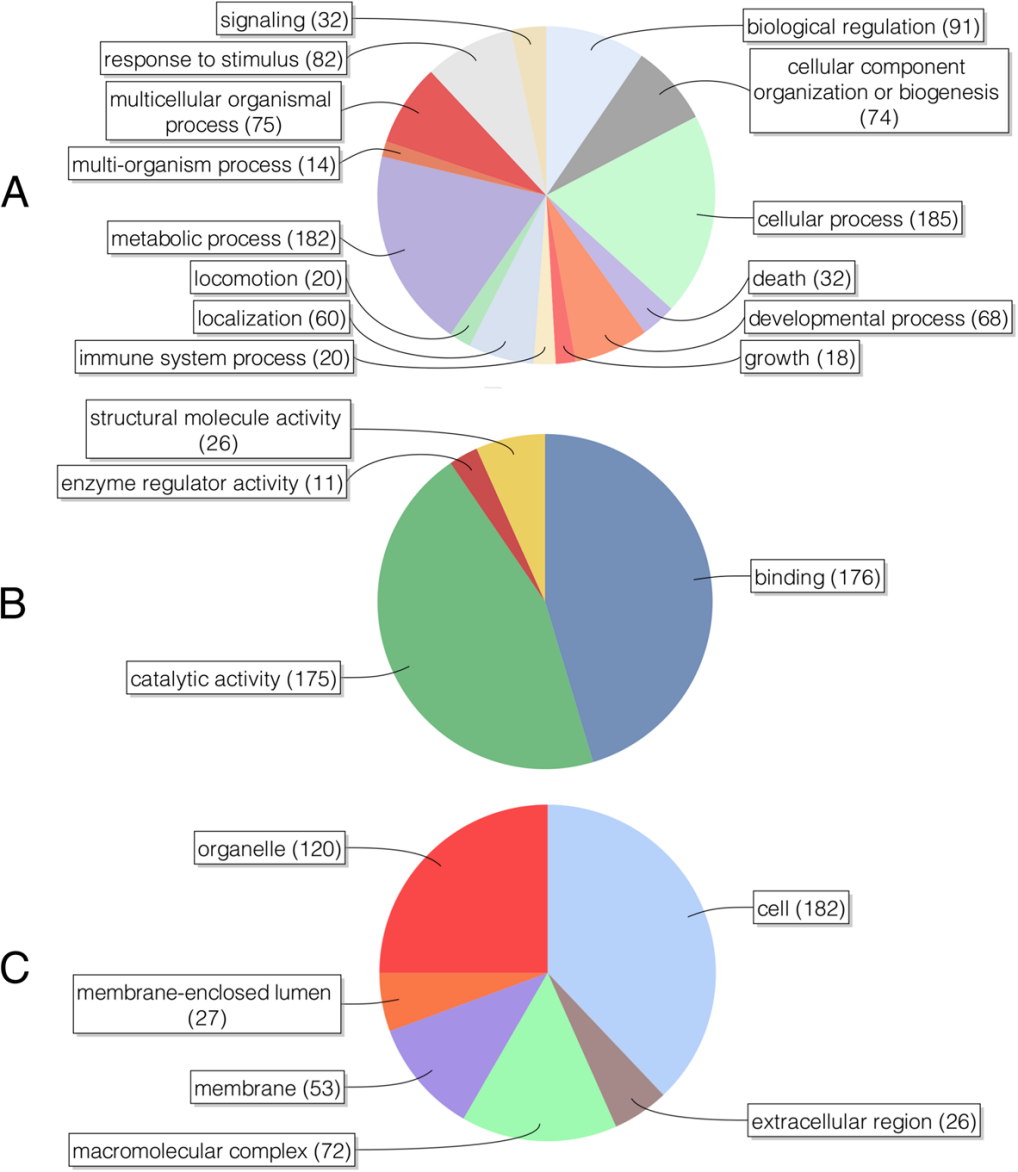
**Gene Ontology level 2 pie charts displaying the biological processes (A), the molecular functions (B) and the cellular components (C) of the 297 proteins that were identified in the *Taenia solium *metacestode excretion/secretion proteins.** Values within parentheses are the number of sequences associated with each Gene Ontology term. The biological processes are mostly metabolic and cellular processes, while the molecular functions are predominantly catalytic activity and binding. The cellular components reveal a number of intercellular proteins. All charts were created using Blast2GO with the sequence filter set to 10.

Gene Ontology level 2 pie charts were created for biological process (Figure 1A), molecular function (Figure 1B) and cellular component (Figure 1C). To avoid overly busy charts, the sequence filter was set to 10. The two largest categories of the biological process chart were cellular and metabolic processes. Others included biological regulation, response to stimulus, multicellular organismal processes and cellular component organization or biogenesis. Further investigation of the general cellular and metabolic processes revealed primary and cellular metabolic processes at level 3 and protein, cellular macromolecule and cellular nitrogen compound metabolic processes at level 4 (Additional file 3, tab 1). Molecular function was clearly divided between binding and catalytic activity. GO level 3 showed protein binding and hydrolase activity while level 4 entailed mostly nucleotide binding, hydrolase activity (acting on acid anhydrides), cation binding, peptidase activity, cytoskeletal and identical protein binding (Additional file 3, tab 2). The level 2 pie chart for the cellular component indicated cell and organelle as the largest categories. Further analyses showed mostly cell part and membrane-bound organelle, and intracellular (part) GO terms at levels 3 and 4, respectively (Additional file 3, tab 3). Human Keratin and porcine Trypsin were identified in all samples. As Keratin is a common contamination and Trypsin was deliberately added during the LC-MS/MS experiments, both were omitted from the final results.

The presence of intracellular/non-secreted proteins in the ESPs is interesting and has been observed in other ESP studies before [22,23]. Although it is highly likely that the majority of those proteins are indeed excreted or secreted by the parasite, the possibility that they are the result of leakage due to cyst damage or death should not be excluded.

In general, the findings reported in this study are comparable to recent studies on other helminth genera like *Echinococcus*[23], *Schistosoma*[24] and *Clonorchis*[25], indicating that excretion/secretion proteomes are not very different between helminth genera/species.

### Comparison between the two studies

When comparing the level 2 GO terms identified in both studies (Table 2), all GO terms from the previous study were identified here as well. Additionally, we identified 6 new GO terms with the EST analyses: rhythmic process (GO:0048511), antioxidant activity (GO:0016209), molecular transducer activity (GO:0060089), protein binding transcription factor activity (GO:0000988), receptor activity (GO:0004872) and synapse (GO:0045202). Although a direct comparison between numbers should be avoided (due to proteins having multiple GOs and the presence of homologous proteins in the proteins groups, especially in the previous study where it is a logical result of the target database construction), the general levels of abundance (= proteins in each GO term) are largely comparable between the two studies e.g. in both studies, cellular process, metabolic process and biological stimulation are the largest groups for ‘biological process’ while binding and catalytic activity are the largest groups for ‘molecular function’ and cell and organelle are the largest groups for ‘cellular component’. The 6 new GO terms were identified by a very small number of proteins and may be a result of proteins being linked to multiple GO terms. This is supported by the fact that the proteins linked to these GO terms are homologous to other proteins identified in both studies, so none of these GO terms was identified by a ’new’ protein group.

**Table 2:** Gene Ontology level 2 annotations identified in this study alongside the ones identified in the previous study

**Gene Ontology information**		**Current**	**Previous**
		**(EST) study**	**study [**[6]**]**
Biological process			
cellular process	GO:0009987	185	162
metabolic process	GO:0008152	182	150
biological regulation	GO:0065007	91	153
response to stimulus	GO:0050896	82	147
multicellular organismal process	GO:0032501	75	107
cellular component organization or biogenesis	GO:0071840	74	92
developmental process	GO:0032502	68	85
localization	GO:0051179	60	88
signaling	GO:0023052	32	55
death	GO:0016265	32	65
immune system process	GO:0002376	20	59
locomotion	GO:0040011	20	29
growth	GO:0040007	18	28
multi-organism process	GO:0051704	14	65
reproduction	GO:0000003	10	36
biological adhesion	GO:0022610	9	13
viral reproduction	GO:0016032	8	12
cell proliferation	GO:0008283	5	24
cell killing	GO:0001906	2	21
rhythmic process	GO:0048511	2	-
Molecular function			
binding	GO:0005488	176	168
catalytic activity	GO:0003824	175	129
structural molecule activity	GO:0005198	26	20
enzyme regulator activity	GO:0030234	11	21
electron carrier activity	GO:0009055	7	13
antioxidant activity	GO:0016209	7	-
transporter activity	GO:0005215	6	14
molecular transducer activity	GO:0060089	4	-
protein binding transcription factor activity	GO:0000988	3	-
nucleic acid binding transcription factor activity	GO:0001071	2	17
receptor activity	GO:0004872	1	-
Cellular component			
cell	GO:0005623	182	168
organelle	GO:0043226	120	155
macromolecular complex	GO:0032991	72	79
membrane	GO:0016020	53	76
membrane-enclosed lumen	GO:0031974	27	63
extracellular region	GO:0005576	26	71
extracellular matrix	GO:0031012	10	11
synapse	GO:0045202	5	-
cell junction	GO:0030054	2	12

Although a direct comparison between numbers should be avoided (due to proteins having multiple GOs and the presence of homologous proteins in the proteins groups, especially in the previous study where it is a logical result of the target database construction), the general levels of abundance are largely comparable between the two studies. Additionally, this study revealed 6 new GO annotations.

## Concluding remarks

In this study, we have used a library of translated ESTs combined with BLAST and mapping strategies not only to confirm previously identified *T. solium* metacestode excretion/secretion proteins, but to identify several new proteins as well, thereby effectively increasing the overall number of protein identifications.

The larger and more complete the EST database, the better proteomic coverage likely obtained. No ESTs from other Taeniidae were used in this study, since the available *T. solium* ESTs were already a merge of EST submissions by different groups and were therefore likely to offer decent proteome coverage. However, in cases where only a small EST library is available with low coverage, one could also include protein sequences and/or ESTs from related organisms in a combined database. This may be particularly advantageous in proteomic studies on less studied, unsequenced, organisms. It should be noted that research on non-sequenced organisms mostly relies on homology to already existing proteins from other (preferably closely related) organisms. Therefore, there is no possibility of finding unique proteins, unless (i) *de novo* sequencing is performed on the good quality unmatched experimental spectra or (ii) ESTs that were identified by spectra but remained unmatched during BLAST are further investigated.

Finally, it is important to realize that, although the mapping to the *E. granulosus* supercontigs helped to remove *S. scrofa* host proteins (e.g. Albumin, Protegrin and Hemopexin), some may still be present. Heat shock protein 70, for example, is identified both in *S. scrofa* and *E. granulosus*.

In future *T. solium* work, it is sensible to make use of the *T. solium* genome sequence that was recently published [5]. However, since no curated protein database or convenient mapping solution is currently available and, for many other helminths, no complete genome sequence is available, the method described here is still valid.

## Availability of supporting data

The data sets supporting the results of this article are available in the PRIDE repository at http://www.ebi.ac.uk/pridewith accession numbers 19232 – 19267.

## Abbreviations

BLAST: Basic Local Alignment Search Tool; ESPs: Excretion/Secretion Proteins; ESTs: Expressed Sequence Tags; GO: Gene Ontology; LC-MS/MS: Liquid Chromatography and tandem Mass Spectrometry; NCBI: National Center for Biotechnology Information.

## Competing interests

The authors declare that they have no competing interests.

## Authors’ contributions

BV carried out the LC-MS/MS experiments and the data analyses and drafted the manuscript. AMD and MP supervised the LC-MS/MS experiments and initial bioinformatic efforts. SG, PD, KP, KK designed the study. JL participated in the analysis of the ESTs. All authors have participated in the manuscript preparation. All authors read and approved the final manuscript.

## Supplementary Material

Additional file 1 **List of all 297 proteins identified in this study, grouped based on homology, including the 1,787 translated ESTs that are linked to those proteins as well as the protein that represents each group and the TBLASTN scores of the queries to the *Echinococcus granulosus *supercontigs.** Click here for file

Additional file 2 **Protein groups (*n *= 106) identified in *Taenia solium *metacestode excretion/secretion proteins, organized by Gene Ontology annotation information on biological process and molecular function.** Groups marked with an asterisk have been identified in the previous analysis as well. For simplicity, all protein groups are represented by one protein. Click here for file

Additional file 3 Gene Ontology information on biological process (tab 1), molecular function (tab 2) and cellular component (tab 3) including graph levels, GO terms, number of sequences (#Seq), node scores and parents. Click here for file
