# Supplementary material for: Use of expressed sequence tags as an alternative approach for the identification of Taenia solium metacestode excretion/secretion proteins
Source: BMC Res Notes. 2013 Jun 6;6:224. doi: 10.1186/1756-0500-6-224 (PMC3686625; doi:10.1186/1756-0500-6-224)
Supplement: Additional file 1 — List of all 297 proteins identified in this study, grouped based on homology, including the 1,787 translated ESTs that are linked to those proteins as well as the protein that represents each group and the TBLASTN scores of the queries to the Echinococcus granulosus supercontigs. [file 1756-0500-6-224-S1.pdf]

Table 1: List of all 297 proteins identified in this study, grouped based on homology, including the 1,787 translated ESTs that are linked to those proteins as well as the protein that represents each group and the TBLASTN scores of the queries to the *Echinococcus granulosus* supercontigs.

**Protein representing the protein group**<br>>Protein identified by ESTs<br>EST	number of ESTs linked to protein	TBLASTN score
**Fibrillar collagen**		
>gi|401653303|gb|EJS70851.1| hypothetical protein ICW_01872, partial [Bacillus cereus BAG2X1-2]	found 1 time	342
EL748816_1 LV0346006 Taenia solium UNAM-cd2_larva Taenia solium cDNA, mRNA sequence		
>gi|225868801|ref|YP_002744749.1| collagen-like cell surface-anchored protein SclF [Streptococcus equi subsp. zooepidemicus]	found 1 time	257
EL754829_1 AD0023085 Taenia solium UNAM-cd1_adult Taenia solium cDNA, mRNA sequence		
>gi|386362622|ref|YP_006071953.1| LPXTG-motif cell wall anchor domain protein [Streptococcus pyogenes Alab49]	found 1 time	373
EL750075_2 AD0003217 Taenia solium UNAM-cd1_adult Taenia solium cDNA, mRNA sequence		
>gi|56759180|gb|AAW27730.1| SJCHGC09290 protein [Schistosoma japonicum]	found 1 time	299
EL751142_1 AD0008153 Taenia solium UNAM-cd1_adult Taenia solium cDNA, mRNA sequence		
>gi|109939862|gb|AAI18145.1| COL27A1 protein [Bos taurus]	found 1 time	257
EL758263_2 AD0103007 Taenia solium UNAM-cd1_adult Taenia solium cDNA, mRNA sequence		
>gi|256085618|ref|XP_002579013.1| Collagen alpha-1(V) chain precursor [Schistosoma mansoni]	found 2 times	489
EL756789_3 AD0031062 Taenia solium UNAM-cd1_adult Taenia solium cDNA, mRNA sequence		
EL751700_2 AD0010193 Taenia solium UNAM-cd1_adult Taenia solium cDNA, mRNA sequence		
>gi|360043236|emb|CCD78649.1| putative collagen alpha-1(V) chain precursor [Schistosoma mansoni]	found 1 time	489
EL753192_2 AD0016230 Taenia solium UNAM-cd1_adult Taenia solium cDNA, mRNA sequence		
>gi|353231962|emb|CCD79317.1| putative collagen alpha-1(I) chain precursor [Schistosoma mansoni]	found 1 time	314
EL753089_1 AD0016127 Taenia solium UNAM-cd1_adult Taenia solium cDNA, mRNA sequence		
>gi|124783696|gb|ABN14936.1| fibrillar collagen [Taenia asiatica]	found 7 times	542
EL762686_3 AD0191047 Taenia solium UNAM-cd1_adult Taenia solium cDNA, mRNA sequence		
EL761576_4 AD0166017 Taenia solium UNAM-cd1_adult Taenia solium cDNA, mRNA sequence		
EL758468_1 AD0106011 Taenia solium UNAM-cd1_adult Taenia solium cDNA, mRNA sequence		
EL756433_2 AD0029228 Taenia solium UNAM-cd1_adult Taenia solium cDNA, mRNA sequence		
EL749195_3 LV0355009 Taenia solium UNAM-cd2_larva Taenia solium cDNA, mRNA sequence		
EL747903_3 LV0330068 Taenia solium UNAM-cd2_larva Taenia solium cDNA, mRNA sequence		
EL745857_4 LV0298014 Taenia solium UNAM-cd2_larva Taenia solium cDNA, mRNA sequence		
**Tubulin polymerization-promoting protein**		
>gi|56755069|gb|AAW25714.1| SJCHGC01071 protein [Schistosoma japonicum]	found 1 time	469
EL758975_2 AD0114045 Taenia solium UNAM-cd1_adult Taenia solium cDNA, mRNA sequence		
>gi|358253100|dbj|GAA52055.1| tubulin polymerization-promoting protein [Clonorchis sinensis]	found 1 time	414
EL757727_2 AD0095002 Taenia solium UNAM-cd1_adult Taenia solium cDNA, mRNA sequence		
**Ferritin**		
>gi|1297064|emb|CAA65097.1| ferritin [Taenia saginata]	found 15 times	533
EL761895_4 AD0173016 Taenia solium UNAM-cd1_adult Taenia solium cDNA, mRNA sequence		
EL761532_5 AD0165030 Taenia solium UNAM-cd1_adult Taenia solium cDNA, mRNA sequence		
EL761264_4 AD0160015 Taenia solium UNAM-cd1_adult Taenia solium cDNA, mRNA sequence		
EL760465_2 AD0143007 Taenia solium UNAM-cd1_adult Taenia solium cDNA, mRNA sequence		
EL759831_3 AD0130021 Taenia solium UNAM-cd1_adult Taenia solium cDNA, mRNA sequence		
EL758273_2 AD0103017 Taenia solium UNAM-cd1_adult Taenia solium cDNA, mRNA sequence		
EL757703_1 AD0094043 Taenia solium UNAM-cd1_adult Taenia solium cDNA, mRNA sequence		
EL752728_3 AD0014269 Taenia solium UNAM-cd1_adult Taenia solium cDNA, mRNA sequence		
EL750419_5 AD0005082 Taenia solium UNAM-cd1_adult Taenia solium cDNA, mRNA sequence		
EL750172_1 AD0004087 Taenia solium UNAM-cd1_adult Taenia solium cDNA, mRNA sequence		
EL749010_1 LV0350019 Taenia solium UNAM-cd2_larva Taenia solium cDNA, mRNA sequence		
EL748286_2 LV0337007 Taenia solium UNAM-cd2_larva Taenia solium cDNA, mRNA sequence		
EL745135_2 LV0286052 Taenia solium UNAM-cd2_larva Taenia solium cDNA, mRNA sequence		
EL744792_2 LV0280018 Taenia solium UNAM-cd2_larva Taenia solium cDNA, mRNA sequence		
EL741550_1 LV0228032 Taenia solium UNAM-cd2_larva Taenia solium cDNA, mRNA sequence		
**RAB GDP dissociation inhibitor alpha**		
>gi|4585567|gb|AAD25536.1| RAB GDP dissociation inhibitor alpha [Rattus norvegicus]	found 2 times	277
EL763087_3 AD0198025 Taenia solium UNAM-cd1_adult Taenia solium cDNA, mRNA sequence		
EL751623_2 AD0010116 Taenia solium UNAM-cd1_adult Taenia solium cDNA, mRNA sequence		
>gi|312285586|gb|ADQ64483.1| hypothetical protein [Bactrocera oleae]	found 1 time	447
GT227872_3 tscaa0_002626.z1.scf Taenia solium adult full-length cDNA library Taenia solium cDNA clone tscaa0_002626.z1.scf 5', mRNA sequence		
**Immunogenic protein Ts11**		
>gi|7339849|gb|AAF60974.1| immunogenic protein Ts11 [Taenia solium]	found 3 times	407
EL758313_2 AD0103057 Taenia solium UNAM-cd1_adult Taenia solium cDNA, mRNA sequence		
EL742536_1 LV0243034 Taenia solium UNAM-cd2_larva Taenia solium cDNA, mRNA sequence		
EL740489_2 LV0210013 Taenia solium UNAM-cd2_larva Taenia solium cDNA, mRNA sequence		
**Triosephosphate isomerase**		
>gi|38258647|sp|Q9GTX8.1|TPIS_TAESO RecName: Full=Triosephosphate isomerase; Short=TIM; AltName: Full=Triosephosphate isomerase	found 1 time	509
EL749505_1 AD0001128 Taenia solium UNAM-cd1_adult Taenia solium cDNA, mRNA sequence		
>gi|80971510|ref|NP_001032228.1| triosephosphate isomerase [Sus scrofa]	found 1 time	350
sp|Q29371|TPIS_PIG Triosephosphate isomerase OS=Sus scrofa GN=TPI1 PE=2 SV=4		
**Adenylosuccinate synthetase**		
>gi|387912858|sp|C4QCD2.2|PURA_SCHMA RecName: Full=Adenylosuccinate synthetase; Short=AMPSase; Short=AdSS; AltName: Full=IMP–aspartate ligase	found 4 times	426
EL761387_6 AD0162048 Taenia solium UNAM-cd1_adult Taenia solium cDNA, mRNA sequence		
EL761074_6 AD0156014 Taenia solium UNAM-cd1_adult Taenia solium cDNA, mRNA sequence		
EL753435_4 AD0017193 Taenia solium UNAM-cd1_adult Taenia solium cDNA, mRNA sequence		
EL750408_4 AD0005071 Taenia solium UNAM-cd1_adult Taenia solium cDNA, mRNA sequence		
**Protein-l-isoaspartate o-methyltransferase**		

Table 1: Continued.

**Protein representing protein group**<br>>Protein identified by ESTs<br>EST	number of ESTs linked to protein	TBLASTN score
>gi|256081696|ref|XP_002577104.1| protein-l-isoaspartate o-methyltransferase [Schistosoma mansoni]	found 2 times	240
EL754820_1 AD0023076 Taenia solium UNAM-cd1_adult Taenia solium cDNA, mRNA sequence		
EL753156_2 AD0016194 Taenia solium UNAM-cd1_adult Taenia solium cDNA, mRNA sequence		
**Major egg antigen**		
>gi|358336515|dbj|GAA34151.2| major egg antigen [Clonorchis sinensis]	found 2 times	301
EL748439_1 LV0340013 Taenia solium UNAM-cd2_larva Taenia solium cDNA, mRNA sequence		
EL748281_2 LV0337002 Taenia solium UNAM-cd2_larva Taenia solium cDNA, mRNA sequence		
**6-phosphogluconolactonase**		
>gi|56756467|gb|AAW26406.1| SJCHGC05391 protein [Schistosoma japonicum]	found 2 times	350
EL753775_3 AD0019062 Taenia solium UNAM-cd1_adult Taenia solium cDNA, mRNA sequence		
EL751511_3 AD0010004 Taenia solium UNAM-cd1_adult Taenia solium cDNA, mRNA sequence		
>gi|350033949|dbj|GAA34277.1| 6-phosphogluconolactonase [Clonorchis sinensis]	found 2 times	349
EL757250_2 AD0034066 Taenia solium UNAM-cd1_adult Taenia solium cDNA, mRNA sequence		
EL748823_2 LV0346013 Taenia solium UNAM-cd2_larva Taenia solium cDNA, mRNA sequence		
**Paramyosin**		
>gi|42559495|sp|Q8T305.1|MYSP_TAESA RecName: Full=Paramyosin	found 1 time	590
EL751042_3 AD0008053 Taenia solium UNAM-cd1_adult Taenia solium cDNA, mRNA sequence		
>gi|62178036|gb|AAX73178.1| putative paramyosin, partial [Echinococcus granulosus]	found 1 time	649
GT227969_1 tscaa0_002868.z1.scf Taenia solium adult full-length cDNA library Taenia solium cDNA clone tscaa0_002868.z1.scf 5', mRNA sequence		
**H17g protein, tegumental antigen**		
>gi|34368418|emb|CAE46111.1| H17g protein, tegumental antigen [Taenia solium]	found 6 times	503
GT889483_3 TSAB.R47.esd Taenia solium cysticerca cDNA library Taenia solium cDNA, mRNA sequence		
GT890601_3 TSAB.R29.esd Taenia solium cysticerca cDNA library Taenia solium cDNA, mRNA sequence		
EL746514_2 LV0310011 Taenia solium UNAM-cd2_larva Taenia solium cDNA, mRNA sequence		
EL745839_1 LV0297068 Taenia solium UNAM-cd2_larva Taenia solium cDNA, mRNA sequence		
EL744814_3 LV0280040 Taenia solium UNAM-cd2_larva Taenia solium cDNA, mRNA sequence		
EL742870_3 LV0248032 Taenia solium UNAM-cd2_larva Taenia solium cDNA, mRNA sequence		
>gi|1495818|emb|CAA65728.1| myosin-like protein [Taenia saginata]	found 4 times	503
EL747144_2 LV0319003 Taenia solium UNAM-cd2_larva Taenia solium cDNA, mRNA sequence		
EL743499_3 LV0258039 Taenia solium UNAM-cd2_larva Taenia solium cDNA, mRNA sequence		
EL741262_3 LV0223040 Taenia solium UNAM-cd2_larva Taenia solium cDNA, mRNA sequence		
EL741177_3 LV0222010 Taenia solium UNAM-cd2_larva Taenia solium cDNA, mRNA sequence		
>gi|124783806|gb|ABN14945.1| myosin [Taenia asiatica]	found 1 time	503
GT227048_2 tscaa0_002818.z1.scf Taenia solium adult full-length cDNA library Taenia solium cDNA clone tscaa0_002818.z1.scf 5', mRNA sequence		
**Annexin**		
>gi|63029698|gb|AAY27744.1| annexin B3 [Taenia solium]	found 5 times	306
EL761467_4 AD0164023 Taenia solium UNAM-cd1_adult Taenia solium cDNA, mRNA sequence		
EL757794_2 AD0095069 Taenia solium UNAM-cd1_adult Taenia solium cDNA, mRNA sequence		
EL756122_1 AD0028180 Taenia solium UNAM-cd1_adult Taenia solium cDNA, mRNA sequence		
EL743370_2 LV0256031 Taenia solium UNAM-cd2_larva Taenia solium cDNA, mRNA sequence		
EL742716_3 LV0245071 Taenia solium UNAM-cd2_larva Taenia solium cDNA, mRNA sequence		
>gi|149721769|ref|XP_001497917.1| PREDICTED: annexin A13-like [Equus caballus]	found 1 time	244
EL746770_1 LV0313053 Taenia solium UNAM-cd2_larva Taenia solium cDNA, mRNA sequence		
>gi|338716868|ref|XP_003363530.1| PREDICTED: annexin A7-like isoform 3 [Equus caballus]	found 1 time	323
EL746641_2 LV0312002 Taenia solium UNAM-cd2_larva Taenia solium cDNA, mRNA sequence		
>gi|358340880|dbj|GAA48684.1| annexin A7 [Clonorchis sinensis]	found 1 time	270
EL749463_2 AD0001086 Taenia solium UNAM-cd1_adult Taenia solium cDNA, mRNA sequence		
>gi|4960053|gb|AAD34598.1|AF147955_1 antigen cC1 [Taenia solium]	found 4 times	299
GT893347_2 TSAD.R50.esd Taenia solium cysticerca cDNA library Taenia solium cDNA, mRNA sequence		
GT227873_3 tscaa0_002630.z1.scf Taenia solium adult full-length cDNA library Taenia solium cDNA clone tscaa0_002630.z1.scf 5', mRNA sequence		
EL743513_2 LV0258053 Taenia solium UNAM-cd2_larva Taenia solium cDNA, mRNA sequence		
EL742803_2 LV0247021 Taenia solium UNAM-cd2_larva Taenia solium cDNA, mRNA sequence		
**Alpha-actinin isoform B**		
>gi|124783372|gb|ABN14924.1| alpha-actinin isoform B [Taenia asiatica]	found 3 times	465
GT892417_3 TSAT.R96.esd Taenia solium cysticerca cDNA library Taenia solium cDNA, mRNA sequence		
GT889841_3 TSCA.R66.esd Taenia solium cysticerca cDNA library Taenia solium cDNA, mRNA sequence		
EL746716_3 LV0312077 Taenia solium UNAM-cd2_larva Taenia solium cDNA, mRNA sequence		
**Charged multivesicular body protein**		
>gi|242247335|ref|NP_001156086.1| charged multivesicular body protein 3-like [Acyrthosiphon pisum]	found 1 time	249
EL761770_6 AD0170041 Taenia solium UNAM-cd1_adult Taenia solium cDNA, mRNA sequence		
>gi|349933025|dbj|GAA29015.1| charged multivesicular body protein 3 [Clonorchis sinensis]	found 2 times	256
EL757762_3 AD0095037 Taenia solium UNAM-cd1_adult Taenia solium cDNA, mRNA sequence		
EL747656_1 LV0326072 Taenia solium UNAM-cd2_larva Taenia solium cDNA, mRNA sequence		
>gi|327275059|ref|XP_003222291.1| PREDICTED: charged multivesicular body protein 5-like [Anolis carolinensis]	found 1 time	285
EL747036_3 LV0317033 Taenia solium UNAM-cd2_larva Taenia solium cDNA, mRNA sequence		
**Laminin**		
>gi|256080034|ref|XP_002576288.1| laminin beta chain-related [Schistosoma mansoni]	found 1 time	424
EL744355_1 LV0271037 Taenia solium UNAM-cd2_larva Taenia solium cDNA, mRNA sequence		
>gi|358334367|dbj|GAA52816.1| laminin alpha 1/2, partial [Clonorchis sinensis]	found 1 time	324
EL744833_1 LV0280059 Taenia solium UNAM-cd2_larva Taenia solium cDNA, mRNA sequence		

Table 1: Continued.

<table>
<tr><th>Protein representing protein group
>Protein identified by ESTs
EST</th><th>number of ESTs linked to protein</th><th>TBLASTN score</th></tr>
<tr><td>14-3-3 protein</td><td></td><td></td></tr>
<tr><td>>gi|29336772|sp|Q8MM75.1|14332_ECHMU RecName: Full=14-3-3 protein homolog 2</td><td>found 7 times</td><td>660</td></tr>
<tr><td>GT890818_1 TSAR.R55.esd Taenia solium cysticerca cDNA library Taenia solium cDNA, mRNA sequence</td><td></td><td></td></tr>
<tr><td>EL761411_5 AD0163020 Taenia solium UNAM-cd1_adult Taenia solium cDNA, mRNA sequence</td><td></td><td></td></tr>
<tr><td>EL760857_1 AD0151014 Taenia solium UNAM-cd1_adult Taenia solium cDNA, mRNA sequence</td><td></td><td></td></tr>
<tr><td>EL759863_2 AD0131002 Taenia solium UNAM-cd1_adult Taenia solium cDNA, mRNA sequence</td><td></td><td></td></tr>
<tr><td>EL758797_3 AD0111062 Taenia solium UNAM-cd1_adult Taenia solium cDNA, mRNA sequence</td><td></td><td></td></tr>
<tr><td>EL755390_3 AD0025160 Taenia solium UNAM-cd1_adult Taenia solium cDNA, mRNA sequence</td><td></td><td></td></tr>
<tr><td>EL748382_1 LV0339020 Taenia solium UNAM-cd2_larva Taenia solium cDNA, mRNA sequence</td><td></td><td></td></tr>
<tr><td>>gi|29336773|sp|Q8MUA4.1|14332_ECHGR RecName: Full=14-3-3 protein homolog 2</td><td>found 7 times</td><td>655</td></tr>
<tr><td>GT892214_3 TSAC.R55.esd Taenia solium cysticerca cDNA library Taenia solium cDNA, mRNA sequence</td><td></td><td></td></tr>
<tr><td>EL758967_3 AD0114037 Taenia solium UNAM-cd1_adult Taenia solium cDNA, mRNA sequence</td><td></td><td></td></tr>
<tr><td>EL754546_3 AD0022071 Taenia solium UNAM-cd1_adult Taenia solium cDNA, mRNA sequence</td><td></td><td></td></tr>
<tr><td>EL753910_2 AD0019197 Taenia solium UNAM-cd1_adult Taenia solium cDNA, mRNA sequence</td><td></td><td></td></tr>
<tr><td>EL748836_2 LV0346026 Taenia solium UNAM-cd2_larva Taenia solium cDNA, mRNA sequence</td><td></td><td></td></tr>
<tr><td>EL744546_1 LV0275021 Taenia solium UNAM-cd2_larva Taenia solium cDNA, mRNA sequence</td><td></td><td></td></tr>
<tr><td>EL741862_1 LV0232067 Taenia solium UNAM-cd2_larva Taenia solium cDNA, mRNA sequence</td><td></td><td></td></tr>
<tr><td>>gi|365266851|gb|AEW70333.1| 14-3-3 zeta [Bombus terrestris]</td><td>found 1 time</td><td>692</td></tr>
<tr><td>EL761132_5 AD0157028 Taenia solium UNAM-cd1_adult Taenia solium cDNA, mRNA sequence</td><td></td><td></td></tr>
<tr><td>>gi|365266872|gb|AEW70348.1| 14-3-3 zeta [Megachile rotundata]</td><td>found 4 times</td><td>693</td></tr>
<tr><td>EL762210_4 AD0181007 Taenia solium UNAM-cd1_adult Taenia solium cDNA, mRNA sequence</td><td></td><td></td></tr>
<tr><td>EL750971_4 AD0007207 Taenia solium UNAM-cd1_adult Taenia solium cDNA, mRNA sequence</td><td></td><td></td></tr>
<tr><td>EL744383_2 LV0271065 Taenia solium UNAM-cd2_larva Taenia solium cDNA, mRNA sequence</td><td></td><td></td></tr>
<tr><td>EL744019_3 LV0266019 Taenia solium UNAM-cd2_larva Taenia solium cDNA, mRNA sequence</td><td></td><td></td></tr>
<tr><td>>gi|239736502|gb|ACS12990.1| 14-3-3zeta [Helicoverpa armigera]</td><td>found 1 time</td><td>681</td></tr>
<tr><td>EL756700_2 AD0030232 Taenia solium UNAM-cd1_adult Taenia solium cDNA, mRNA sequence</td><td></td><td></td></tr>
<tr><td>>gi|346471453|gb|AEO35571.1| hypothetical protein [Amblyomma maculatum]</td><td>found 3 times</td><td>710</td></tr>
<tr><td>EL763008_2 AD0197017 Taenia solium UNAM-cd1_adult Taenia solium cDNA, mRNA sequence</td><td></td><td></td></tr>
<tr><td>EL761548_6 AD0165046 Taenia solium UNAM-cd1_adult Taenia solium cDNA, mRNA sequence</td><td></td><td></td></tr>
<tr><td>EL749382_2 AD0001005 Taenia solium UNAM-cd1_adult Taenia solium cDNA, mRNA sequence</td><td></td><td></td></tr>
<tr><td>>gi|351724401|ref|NP_001238592.1| 14-3-3 protein SGF14n [Glycine max]</td><td>found 1 time</td><td>662</td></tr>
<tr><td>EL744470_2 LV0273028 Taenia solium UNAM-cd2_larva Taenia solium cDNA, mRNA sequence</td><td></td><td></td></tr>
<tr><td>>gi|348537740|ref|XP_003456351.1| PREDICTED: 14-3-3 protein epsilon-like [Oreochromis niloticus]</td><td>found 2 times</td><td>714</td></tr>
<tr><td>EL742306_3 LV0240001 Taenia solium UNAM-cd2_larva Taenia solium cDNA, mRNA sequence</td><td></td><td></td></tr>
<tr><td>EL742049_3 LV0235038 Taenia solium UNAM-cd2_larva Taenia solium cDNA, mRNA sequence</td><td></td><td></td></tr>
<tr><td>8 kDa protein family</td><td></td><td></td></tr>
<tr><td>>gi|23477224|emb|CAD48846.1| secreted antigen Ts8B2 [Taenia solium]</td><td>found 39 times</td><td>201</td></tr>
<tr><td>EL748720_2 LV0344043 Taenia solium UNAM-cd2_larva Taenia solium cDNA, mRNA sequence</td><td></td><td></td></tr>
<tr><td>EL747775_3 LV0329013 Taenia solium UNAM-cd2_larva Taenia solium cDNA, mRNA sequence</td><td></td><td></td></tr>
<tr><td>EL746760_1 LV0313043 Taenia solium UNAM-cd2_larva Taenia solium cDNA, mRNA sequence</td><td></td><td></td></tr>
<tr><td>EL746611_3 LV0311040 Taenia solium UNAM-cd2_larva Taenia solium cDNA, mRNA sequence</td><td></td><td></td></tr>
<tr><td>EL746005_3 LV0300036 Taenia solium UNAM-cd2_larva Taenia solium cDNA, mRNA sequence</td><td></td><td></td></tr>
<tr><td>EL745836_1 LV0297065 Taenia solium UNAM-cd2_larva Taenia solium cDNA, mRNA sequence</td><td></td><td></td></tr>
<tr><td>EL745829_3 LV0297058 Taenia solium UNAM-cd2_larva Taenia solium cDNA, mRNA sequence</td><td></td><td></td></tr>
<tr><td>EL745694_3 LV0295052 Taenia solium UNAM-cd2_larva Taenia solium cDNA, mRNA sequence</td><td></td><td></td></tr>
<tr><td>EL745213_3 LV0287067 Taenia solium UNAM-cd2_larva Taenia solium cDNA, mRNA sequence</td><td></td><td></td></tr>
<tr><td>EL745152_3 LV0287006 Taenia solium UNAM-cd2_larva Taenia solium cDNA, mRNA sequence</td><td></td><td></td></tr>
<tr><td>EL745081_1 LV0285053 Taenia solium UNAM-cd2_larva Taenia solium cDNA, mRNA sequence</td><td></td><td></td></tr>
<tr><td>EL745040_2 LV0285012 Taenia solium UNAM-cd2_larva Taenia solium cDNA, mRNA sequence</td><td></td><td></td></tr>
<tr><td>EL745033_3 LV0285005 Taenia solium UNAM-cd2_larva Taenia solium cDNA, mRNA sequence</td><td></td><td></td></tr>
<tr><td>EL744859_3 LV0281019 Taenia solium UNAM-cd2_larva Taenia solium cDNA, mRNA sequence</td><td></td><td></td></tr>
<tr><td>EL744136_2 LV0268003 Taenia solium UNAM-cd2_larva Taenia solium cDNA, mRNA sequence</td><td></td><td></td></tr>
<tr><td>EL744118_1 LV0267057 Taenia solium UNAM-cd2_larva Taenia solium cDNA, mRNA sequence</td><td></td><td></td></tr>
<tr><td>EL744036_3 LV0266036 Taenia solium UNAM-cd2_larva Taenia solium cDNA, mRNA sequence</td><td></td><td></td></tr>
<tr><td>EL744020_3 LV0266020 Taenia solium UNAM-cd2_larva Taenia solium cDNA, mRNA sequence</td><td></td><td></td></tr>
<tr><td>EL743995_1 LV0265067 Taenia solium UNAM-cd2_larva Taenia solium cDNA, mRNA sequence</td><td></td><td></td></tr>
<tr><td>EL743965_1 LV0265037 Taenia solium UNAM-cd2_larva Taenia solium cDNA, mRNA sequence</td><td></td><td></td></tr>
<tr><td>EL743704_2 LV0261013 Taenia solium UNAM-cd2_larva Taenia solium cDNA, mRNA sequence</td><td></td><td></td></tr>
<tr><td>EL743479_1 LV0258019 Taenia solium UNAM-cd2_larva Taenia solium cDNA, mRNA sequence</td><td></td><td></td></tr>
<tr><td>EL743242_1 LV0254023 Taenia solium UNAM-cd2_larva Taenia solium cDNA, mRNA sequence</td><td></td><td></td></tr>
<tr><td>EL743158_3 LV0253005 Taenia solium UNAM-cd2_larva Taenia solium cDNA, mRNA sequence</td><td></td><td></td></tr>
<tr><td>EL743137_2 LV0252038 Taenia solium UNAM-cd2_larva Taenia solium cDNA, mRNA sequence</td><td></td><td></td></tr>
<tr><td>EL742801_3 LV0247019 Taenia solium UNAM-cd2_larva Taenia solium cDNA, mRNA sequence</td><td></td><td></td></tr>
<tr><td>EL742743_3 LV0246026 Taenia solium UNAM-cd2_larva Taenia solium cDNA, mRNA sequence</td><td></td><td></td></tr>
<tr><td>EL742445_2 LV0242009 Taenia solium UNAM-cd2_larva Taenia solium cDNA, mRNA sequence</td><td></td><td></td></tr>
<tr><td>EL742239_3 LV0238058 Taenia solium UNAM-cd2_larva Taenia solium cDNA, mRNA sequence</td><td></td><td></td></tr>
<tr><td>EL742136_1 LV0237024 Taenia solium UNAM-cd2_larva Taenia solium cDNA, mRNA sequence</td><td></td><td></td></tr>
<tr><td>EL742014_2 LV0235003 Taenia solium UNAM-cd2_larva Taenia solium cDNA, mRNA sequence</td><td></td><td></td></tr>
<tr><td>EL741651_1 LV0229062 Taenia solium UNAM-cd2_larva Taenia solium cDNA, mRNA sequence</td><td></td><td></td></tr>
<tr><td>EL741642_3 LV0229053 Taenia solium UNAM-cd2_larva Taenia solium cDNA, mRNA sequence</td><td></td><td></td></tr>
<tr><td>EL741248_1 LV0223026 Taenia solium UNAM-cd2_larva Taenia solium cDNA, mRNA sequence</td><td></td><td></td></tr>
<tr><td>EL740907_2 LV0218018 Taenia solium UNAM-cd2_larva Taenia solium cDNA, mRNA sequence</td><td></td><td></td></tr>
<tr><td>EL740448_3 LV0209026 Taenia solium UNAM-cd2_larva Taenia solium cDNA, mRNA sequence</td><td></td><td></td></tr>
<tr><td>EL740423_3 LV0209001 Taenia solium UNAM-cd2_larva Taenia solium cDNA, mRNA sequence</td><td></td><td></td></tr>
<tr><td>EL740387_2 LV0208007 Taenia solium UNAM-cd2_larva Taenia solium cDNA, mRNA sequence</td><td></td><td></td></tr>
<tr><td>EL740305_2 LV0206037 Taenia solium UNAM-cd2_larva Taenia solium cDNA, mRNA sequence</td><td></td><td></td></tr>
<tr><td>>gi|347546145|gb|AEP03196.1| CyDA variant 1 [Taenia solium]</td><td>found 1 time</td><td>203</td></tr>
<tr><td>EL746418_3 LV0308068 Taenia solium UNAM-cd2_larva Taenia solium cDNA, mRNA sequence</td><td></td><td></td></tr>
<tr><td>>gi|346680629|gb|AEO45118.1| hydrophobic ligand binding protein 1 [Taenia solium]</td><td>found 32 times</td><td>193</td></tr>
<tr><td>GT227063_3 tscaa0_002762.z1.scf Taenia solium adult full-length cDNA library Taenia solium cDNA clone tscaa0_002762.z1.scf 5', mRNA sequence</td><td></td><td></td></tr>
</table>

Table 1: Continued.

**Protein representing protein group**<br>>Protein identified by ESTs<br>EST	number of ESTs linked to protein	TBLASTN score
EL762896_2 AD0195016 Taenia solium UNAM-cd1_adult Taenia solium cDNA, mRNA sequence		
EL762254_4 AD0182010 Taenia solium UNAM-cd1_adult Taenia solium cDNA, mRNA sequence		
EL762140_6 AD0179033 Taenia solium UNAM-cd1_adult Taenia solium cDNA, mRNA sequence		
EL761372_5 AD0162033 Taenia solium UNAM-cd1_adult Taenia solium cDNA, mRNA sequence		
EL761339_5 AD0161056 Taenia solium UNAM-cd1_adult Taenia solium cDNA, mRNA sequence		
EL761211_6 AD0159008 Taenia solium UNAM-cd1_adult Taenia solium cDNA, mRNA sequence		
EL760986_4 AD0154018 Taenia solium UNAM-cd1_adult Taenia solium cDNA, mRNA sequence		
EL760966_5 AD0153040 Taenia solium UNAM-cd1_adult Taenia solium cDNA, mRNA sequence		
EL760933_3 AD0153007 Taenia solium UNAM-cd1_adult Taenia solium cDNA, mRNA sequence		
EL760717_3 AD0147049 Taenia solium UNAM-cd1_adult Taenia solium cDNA, mRNA sequence		
EL759976_3 AD0133021 Taenia solium UNAM-cd1_adult Taenia solium cDNA, mRNA sequence		
EL759888_2 AD0131027 Taenia solium UNAM-cd1_adult Taenia solium cDNA, mRNA sequence		
EL759390_2 AD0122034 Taenia solium UNAM-cd1_adult Taenia solium cDNA, mRNA sequence		
EL759065_3 AD0116028 Taenia solium UNAM-cd1_adult Taenia solium cDNA, mRNA sequence		
EL758738_3 AD0111003 Taenia solium UNAM-cd1_adult Taenia solium cDNA, mRNA sequence		
EL758586_1 AD0108017 Taenia solium UNAM-cd1_adult Taenia solium cDNA, mRNA sequence		
EL758173_1 AD0101053 Taenia solium UNAM-cd1_adult Taenia solium cDNA, mRNA sequence		
EL757925_2 AD0098010 Taenia solium UNAM-cd1_adult Taenia solium cDNA, mRNA sequence		
EL757560_1 AD0035153 Taenia solium UNAM-cd1_adult Taenia solium cDNA, mRNA sequence		
EL757208_2 AD0034024 Taenia solium UNAM-cd1_adult Taenia solium cDNA, mRNA sequence		
EL756367_1 AD0029162 Taenia solium UNAM-cd1_adult Taenia solium cDNA, mRNA sequence		
EL756133_6 AD0028191 Taenia solium UNAM-cd1_adult Taenia solium cDNA, mRNA sequence		
EL755065_2 AD0024082 Taenia solium UNAM-cd1_adult Taenia solium cDNA, mRNA sequence		
EL755005_3 AD0024022 Taenia solium UNAM-cd1_adult Taenia solium cDNA, mRNA sequence		
EL754906_3 AD0023162 Taenia solium UNAM-cd1_adult Taenia solium cDNA, mRNA sequence		
EL754836_4 AD0023092 Taenia solium UNAM-cd1_adult Taenia solium cDNA, mRNA sequence		
EL754478_3 AD0022003 Taenia solium UNAM-cd1_adult Taenia solium cDNA, mRNA sequence		
EL754307_3 AD0021097 Taenia solium UNAM-cd1_adult Taenia solium cDNA, mRNA sequence		
EL754220_1 AD0021010 Taenia solium UNAM-cd1_adult Taenia solium cDNA, mRNA sequence		
EL750405_3 AD0005068 Taenia solium UNAM-cd1_adult Taenia solium cDNA, mRNA sequence		
EL750127_3 AD0004042 Taenia solium UNAM-cd1_adult Taenia solium cDNA, mRNA sequence		
>gi|346680631|gb|AEO45119.1| hydrophobic ligand binding protein 2 [Taenia solium]	found 69 times	193
EL763482_2 AD0204045 Taenia solium UNAM-cd1_adult Taenia solium cDNA, mRNA sequence		
EL763327_4 AD0202027 Taenia solium UNAM-cd1_adult Taenia solium cDNA, mRNA sequence		
EL763248_3 AD0201011 Taenia solium UNAM-cd1_adult Taenia solium cDNA, mRNA sequence		
EL763231_3 AD0200046 Taenia solium UNAM-cd1_adult Taenia solium cDNA, mRNA sequence		
EL762974_1 AD0196027 Taenia solium UNAM-cd1_adult Taenia solium cDNA, mRNA sequence		
EL762726_1 AD0192024 Taenia solium UNAM-cd1_adult Taenia solium cDNA, mRNA sequence		
EL762576_2 AD0189033 Taenia solium UNAM-cd1_adult Taenia solium cDNA, mRNA sequence		
EL762391_3 AD0184032 Taenia solium UNAM-cd1_adult Taenia solium cDNA, mRNA sequence		
EL762312_5 AD0183013 Taenia solium UNAM-cd1_adult Taenia solium cDNA, mRNA sequence		
EL762302_5 AD0183003 Taenia solium UNAM-cd1_adult Taenia solium cDNA, mRNA sequence		
EL762263_4 AD0182019 Taenia solium UNAM-cd1_adult Taenia solium cDNA, mRNA sequence		
EL762226_5 AD0181023 Taenia solium UNAM-cd1_adult Taenia solium cDNA, mRNA sequence		
EL762132_4 AD0179025 Taenia solium UNAM-cd1_adult Taenia solium cDNA, mRNA sequence		
EL762070_3 AD0178004 Taenia solium UNAM-cd1_adult Taenia solium cDNA, mRNA sequence		
EL761966_6 AD0175012 Taenia solium UNAM-cd1_adult Taenia solium cDNA, mRNA sequence		
EL761910_4 AD0173031 Taenia solium UNAM-cd1_adult Taenia solium cDNA, mRNA sequence		
EL761695_5 AD0169012 Taenia solium UNAM-cd1_adult Taenia solium cDNA, mRNA sequence		
EL761549_2 AD0165047 Taenia solium UNAM-cd1_adult Taenia solium cDNA, mRNA sequence		
EL761403_5 AD0163012 Taenia solium UNAM-cd1_adult Taenia solium cDNA, mRNA sequence		
EL761354_1 AD0162015 Taenia solium UNAM-cd1_adult Taenia solium cDNA, mRNA sequence		
EL761296_3 AD0161013 Taenia solium UNAM-cd1_adult Taenia solium cDNA, mRNA sequence		
EL760114_1 AD0136005 Taenia solium UNAM-cd1_adult Taenia solium cDNA, mRNA sequence		
EL759677_2 AD0127018 Taenia solium UNAM-cd1_adult Taenia solium cDNA, mRNA sequence		
EL759349_1 AD0121045 Taenia solium UNAM-cd1_adult Taenia solium cDNA, mRNA sequence		
EL759116_2 AD0117019 Taenia solium UNAM-cd1_adult Taenia solium cDNA, mRNA sequence		
EL759049_6 AD0116012 Taenia solium UNAM-cd1_adult Taenia solium cDNA, mRNA sequence		
EL758909_2 AD0113050 Taenia solium UNAM-cd1_adult Taenia solium cDNA, mRNA sequence		
EL758525_2 AD0107018 Taenia solium UNAM-cd1_adult Taenia solium cDNA, mRNA sequence		
EL758458_1 AD0106001 Taenia solium UNAM-cd1_adult Taenia solium cDNA, mRNA sequence		
EL758167_5 AD0101047 Taenia solium UNAM-cd1_adult Taenia solium cDNA, mRNA sequence		
EL758156_3 AD0101036 Taenia solium UNAM-cd1_adult Taenia solium cDNA, mRNA sequence		
EL758088_2 AD0100033 Taenia solium UNAM-cd1_adult Taenia solium cDNA, mRNA sequence		
EL758028_2 AD0099040 Taenia solium UNAM-cd1_adult Taenia solium cDNA, mRNA sequence		
EL758014_2 AD0099026 Taenia solium UNAM-cd1_adult Taenia solium cDNA, mRNA sequence		
EL757848_3 AD0096051 Taenia solium UNAM-cd1_adult Taenia solium cDNA, mRNA sequence		
EL757812_1 AD0096015 Taenia solium UNAM-cd1_adult Taenia solium cDNA, mRNA sequence		
EL757788_1 AD0095063 Taenia solium UNAM-cd1_adult Taenia solium cDNA, mRNA sequence		
EL757770_6 AD0095045 Taenia solium UNAM-cd1_adult Taenia solium cDNA, mRNA sequence		
EL757646_2 AD0035239 Taenia solium UNAM-cd1_adult Taenia solium cDNA, mRNA sequence		
EL757483_1 AD0035076 Taenia solium UNAM-cd1_adult Taenia solium cDNA, mRNA sequence		
EL757405_1 AD0034221 Taenia solium UNAM-cd1_adult Taenia solium cDNA, mRNA sequence		
EL756872_1 AD0031145 Taenia solium UNAM-cd1_adult Taenia solium cDNA, mRNA sequence		
EL756815_1 AD0031088 Taenia solium UNAM-cd1_adult Taenia solium cDNA, mRNA sequence		
EL756563_2 AD0030095 Taenia solium UNAM-cd1_adult Taenia solium cDNA, mRNA sequence		
EL756479_2 AD0030011 Taenia solium UNAM-cd1_adult Taenia solium cDNA, mRNA sequence		
EL756378_6 AD0029173 Taenia solium UNAM-cd1_adult Taenia solium cDNA, mRNA sequence		
EL756055_4 AD0028113 Taenia solium UNAM-cd1_adult Taenia solium cDNA, mRNA sequence		
EL755673_1 AD0026196 Taenia solium UNAM-cd1_adult Taenia solium cDNA, mRNA sequence		
EL755047_1 AD0024064 Taenia solium UNAM-cd1_adult Taenia solium cDNA, mRNA sequence		
EL754807_3 AD0023063 Taenia solium UNAM-cd1_adult Taenia solium cDNA, mRNA sequence		

Table 1: Continued.

**Protein representing protein group**<br>>Protein identified by ESTs<br>EST	number of ESTs linked to protein	TBLASTN score
EL754641_1 AD0022166 Taenia solium UNAM-cd1_adult Taenia solium cDNA, mRNA sequence		
EL754250_1 AD0021040 Taenia solium UNAM-cd1_adult Taenia solium cDNA, mRNA sequence		
EL754227_6 AD0021017 Taenia solium UNAM-cd1_adult Taenia solium cDNA, mRNA sequence		
EL754152_1 AD0020197 Taenia solium UNAM-cd1_adult Taenia solium cDNA, mRNA sequence		
EL754128_4 AD0020173 Taenia solium UNAM-cd1_adult Taenia solium cDNA, mRNA sequence		
EL753400_3 AD0017158 Taenia solium UNAM-cd1_adult Taenia solium cDNA, mRNA sequence		
EL753165_5 AD0016203 Taenia solium UNAM-cd1_adult Taenia solium cDNA, mRNA sequence		
EL752456_4 AD0013223 Taenia solium UNAM-cd1_adult Taenia solium cDNA, mRNA sequence		
EL752231_1 AD0012259 Taenia solium UNAM-cd1_adult Taenia solium cDNA, mRNA sequence		
EL751826_5 AD0011069 Taenia solium UNAM-cd1_adult Taenia solium cDNA, mRNA sequence		
EL751589_3 AD0010082 Taenia solium UNAM-cd1_adult Taenia solium cDNA, mRNA sequence		
EL751517_2 AD0010010 Taenia solium UNAM-cd1_adult Taenia solium cDNA, mRNA sequence		
EL751497_6 AD0009235 Taenia solium UNAM-cd1_adult Taenia solium cDNA, mRNA sequence		
EL751282_5 AD0009020 Taenia solium UNAM-cd1_adult Taenia solium cDNA, mRNA sequence		
EL751253_1 AD0008264 Taenia solium UNAM-cd1_adult Taenia solium cDNA, mRNA sequence		
EL750852_1 AD0007088 Taenia solium UNAM-cd1_adult Taenia solium cDNA, mRNA sequence		
EL750450_1 AD0005113 Taenia solium UNAM-cd1_adult Taenia solium cDNA, mRNA sequence		
EL749588_5 AD0001211 Taenia solium UNAM-cd1_adult Taenia solium cDNA, mRNA sequence		
EL749541_3 AD0001164 Taenia solium UNAM-cd1_adult Taenia solium cDNA, mRNA sequence		
>gi|13398632|gb|AAK21960.1|AF350071_1 18 kDa glycoprotein variant 2 [Taenia solium]	found 38 times	95
EL747592_1 LV0326008 Taenia solium UNAM-cd2_larva Taenia solium cDNA, mRNA sequence		
EL747529_1 LV0325008 Taenia solium UNAM-cd2_larva Taenia solium cDNA, mRNA sequence		
EL747412_1 LV0323021 Taenia solium UNAM-cd2_larva Taenia solium cDNA, mRNA sequence		
EL747319_2 LV0321050 Taenia solium UNAM-cd2_larva Taenia solium cDNA, mRNA sequence		
EL747315_3 LV0321046 Taenia solium UNAM-cd2_larva Taenia solium cDNA, mRNA sequence		
EL747091_2 LV0318023 Taenia solium UNAM-cd2_larva Taenia solium cDNA, mRNA sequence		
EL746824_1 LV0314037 Taenia solium UNAM-cd2_larva Taenia solium cDNA, mRNA sequence		
EL746565_1 LV0310062 Taenia solium UNAM-cd2_larva Taenia solium cDNA, mRNA sequence		
EL746275_3 LV0306026 Taenia solium UNAM-cd2_larva Taenia solium cDNA, mRNA sequence		
EL745848_3 LV0298005 Taenia solium UNAM-cd2_larva Taenia solium cDNA, mRNA sequence		
EL745701_1 LV0295059 Taenia solium UNAM-cd2_larva Taenia solium cDNA, mRNA sequence		
EL745628_2 LV0294043 Taenia solium UNAM-cd2_larva Taenia solium cDNA, mRNA sequence		
EL745269_1 LV0288051 Taenia solium UNAM-cd2_larva Taenia solium cDNA, mRNA sequence		
EL745055_1 LV0285027 Taenia solium UNAM-cd2_larva Taenia solium cDNA, mRNA sequence		
EL745029_1 LV0285001 Taenia solium UNAM-cd2_larva Taenia solium cDNA, mRNA sequence		
EL744717_1 LV0278036 Taenia solium UNAM-cd2_larva Taenia solium cDNA, mRNA sequence		
EL744621_1 LV0276039 Taenia solium UNAM-cd2_larva Taenia solium cDNA, mRNA sequence		
EL744365_1 LV0271047 Taenia solium UNAM-cd2_larva Taenia solium cDNA, mRNA sequence		
EL744348_1 LV0271030 Taenia solium UNAM-cd2_larva Taenia solium cDNA, mRNA sequence		
EL743806_6 LV0262047 Taenia solium UNAM-cd2_larva Taenia solium cDNA, mRNA sequence		
EL743743_2 LV0261052 Taenia solium UNAM-cd2_larva Taenia solium cDNA, mRNA sequence		
EL743599_3 LV0259062 Taenia solium UNAM-cd2_larva Taenia solium cDNA, mRNA sequence		
EL743237_1 LV0254018 Taenia solium UNAM-cd2_larva Taenia solium cDNA, mRNA sequence		
EL743006_1 LV0250047 Taenia solium UNAM-cd2_larva Taenia solium cDNA, mRNA sequence		
EL742889_3 LV0249004 Taenia solium UNAM-cd2_larva Taenia solium cDNA, mRNA sequence		
EL742831_1 LV0247049 Taenia solium UNAM-cd2_larva Taenia solium cDNA, mRNA sequence		
EL742790_1 LV0247008 Taenia solium UNAM-cd2_larva Taenia solium cDNA, mRNA sequence		
EL742623_1 LV0244054 Taenia solium UNAM-cd2_larva Taenia solium cDNA, mRNA sequence		
EL742304_2 LV0239056 Taenia solium UNAM-cd2_larva Taenia solium cDNA, mRNA sequence		
EL742043_3 LV0235032 Taenia solium UNAM-cd2_larva Taenia solium cDNA, mRNA sequence		
EL741955_1 LV0234016 Taenia solium UNAM-cd2_larva Taenia solium cDNA, mRNA sequence		
EL741822_1 LV0232027 Taenia solium UNAM-cd2_larva Taenia solium cDNA, mRNA sequence		
EL741566_3 LV0228048 Taenia solium UNAM-cd2_larva Taenia solium cDNA, mRNA sequence		
EL741501_2 LV0227057 Taenia solium UNAM-cd2_larva Taenia solium cDNA, mRNA sequence		
EL741488_1 LV0227044 Taenia solium UNAM-cd2_larva Taenia solium cDNA, mRNA sequence		
EL741439_3 LV0226058 Taenia solium UNAM-cd2_larva Taenia solium cDNA, mRNA sequence		
EL740842_3 LV0217025 Taenia solium UNAM-cd2_larva Taenia solium cDNA, mRNA sequence		
EL740516_3 LV0210040 Taenia solium UNAM-cd2_larva Taenia solium cDNA, mRNA sequence		
>gi|13398630|gb|AAK21959.1|AF350070_1 18 kDa glycoprotein variant 1 [Taenia solium]	found 5 times	99
EL748435_1 LV0340009 Taenia solium UNAM-cd2_larva Taenia solium cDNA, mRNA sequence		
EL748026_1 LV0332048 Taenia solium UNAM-cd2_larva Taenia solium cDNA, mRNA sequence		
EL746340_2 LV0307054 Taenia solium UNAM-cd2_larva Taenia solium cDNA, mRNA sequence		
EL746106_3 LV0302001 Taenia solium UNAM-cd2_larva Taenia solium cDNA, mRNA sequence		
EL744566_2 LV0275041 Taenia solium UNAM-cd2_larva Taenia solium cDNA, mRNA sequence		
>gi|347546161|gb|AEP03204.1| Ts18 variant 2 [Taenia solium]	found 76 times	99
EL748106_1 LV0333057 Taenia solium UNAM-cd2_larva Taenia solium cDNA, mRNA sequence		
EL747818_1 LV0329056 Taenia solium UNAM-cd2_larva Taenia solium cDNA, mRNA sequence		
EL747749_1 LV0328037 Taenia solium UNAM-cd2_larva Taenia solium cDNA, mRNA sequence		
EL747514_1 LV0324050 Taenia solium UNAM-cd2_larva Taenia solium cDNA, mRNA sequence		
EL747377_1 LV0322054 Taenia solium UNAM-cd2_larva Taenia solium cDNA, mRNA sequence		
EL747373_1 LV0322050 Taenia solium UNAM-cd2_larva Taenia solium cDNA, mRNA sequence		
EL747285_1 LV0321016 Taenia solium UNAM-cd2_larva Taenia solium cDNA, mRNA sequence		
EL746910_1 LV0315052 Taenia solium UNAM-cd2_larva Taenia solium cDNA, mRNA sequence		
EL746704_2 LV0312065 Taenia solium UNAM-cd2_larva Taenia solium cDNA, mRNA sequence		
EL746362_1 LV0308012 Taenia solium UNAM-cd2_larva Taenia solium cDNA, mRNA sequence		
EL746204_1 LV0304029 Taenia solium UNAM-cd2_larva Taenia solium cDNA, mRNA sequence		
EL746186_1 LV0304011 Taenia solium UNAM-cd2_larva Taenia solium cDNA, mRNA sequence		
EL746130_1 LV0302025 Taenia solium UNAM-cd2_larva Taenia solium cDNA, mRNA sequence		
EL745992_1 LV0300023 Taenia solium UNAM-cd2_larva Taenia solium cDNA, mRNA sequence		
EL745960_1 LV0299049 Taenia solium UNAM-cd2_larva Taenia solium cDNA, mRNA sequence		
EL745955_1 LV0299044 Taenia solium UNAM-cd2_larva Taenia solium cDNA, mRNA sequence		
EL745935_1 LV0299024 Taenia solium UNAM-cd2_larva Taenia solium cDNA, mRNA sequence		

Table 1: Continued.

**Protein representing protein group**<br>>Protein identified by ESTs<br>EST	number of ESTs linked to protein	TBLASTN score
EL745539_1 LV0293017 Taenia solium UNAM-cd2_larva Taenia solium cDNA, mRNA sequence		
EL745518_1 LV0292052 Taenia solium UNAM-cd2_larva Taenia solium cDNA, mRNA sequence		
EL745492_3 LV0292026 Taenia solium UNAM-cd2_larva Taenia solium cDNA, mRNA sequence		
EL745231_1 LV0288013 Taenia solium UNAM-cd2_larva Taenia solium cDNA, mRNA sequence		
EL745203_1 LV0287057 Taenia solium UNAM-cd2_larva Taenia solium cDNA, mRNA sequence		
EL745138_1 LV0286055 Taenia solium UNAM-cd2_larva Taenia solium cDNA, mRNA sequence		
EL745120_1 LV0286037 Taenia solium UNAM-cd2_larva Taenia solium cDNA, mRNA sequence		
EL745088_1 LV0286004 Taenia solium UNAM-cd2_larva Taenia solium cDNA, mRNA sequence		
EL744856_1 LV0281016 Taenia solium UNAM-cd2_larva Taenia solium cDNA, mRNA sequence		
EL744750_3 LV0279019 Taenia solium UNAM-cd2_larva Taenia solium cDNA, mRNA sequence		
EL744734_1 LV0279003 Taenia solium UNAM-cd2_larva Taenia solium cDNA, mRNA sequence		
EL744710_3 LV0278029 Taenia solium UNAM-cd2_larva Taenia solium cDNA, mRNA sequence		
EL744610_1 LV0276028 Taenia solium UNAM-cd2_larva Taenia solium cDNA, mRNA sequence		
EL744520_1 LV0274023 Taenia solium UNAM-cd2_larva Taenia solium cDNA, mRNA sequence		
EL744420_1 LV0272035 Taenia solium UNAM-cd2_larva Taenia solium cDNA, mRNA sequence		
EL744372_1 LV0271054 Taenia solium UNAM-cd2_larva Taenia solium cDNA, mRNA sequence		
EL744370_1 LV0271052 Taenia solium UNAM-cd2_larva Taenia solium cDNA, mRNA sequence		
EL744318_1 LV0270065 Taenia solium UNAM-cd2_larva Taenia solium cDNA, mRNA sequence		
EL744213_1 LV0269024 Taenia solium UNAM-cd2_larva Taenia solium cDNA, mRNA sequence		
EL744205_1 LV0269016 Taenia solium UNAM-cd2_larva Taenia solium cDNA, mRNA sequence		
EL744162_1 LV0268029 Taenia solium UNAM-cd2_larva Taenia solium cDNA, mRNA sequence		
EL744044_1 LV0266044 Taenia solium UNAM-cd2_larva Taenia solium cDNA, mRNA sequence		
EL743994_1 LV0265066 Taenia solium UNAM-cd2_larva Taenia solium cDNA, mRNA sequence		
EL743967_1 LV0265039 Taenia solium UNAM-cd2_larva Taenia solium cDNA, mRNA sequence		
EL743909_1 LV0264038 Taenia solium UNAM-cd2_larva Taenia solium cDNA, mRNA sequence		
EL743903_1 LV0264032 Taenia solium UNAM-cd2_larva Taenia solium cDNA, mRNA sequence		
EL743440_1 LV0257057 Taenia solium UNAM-cd2_larva Taenia solium cDNA, mRNA sequence		
EL743322_1 LV0255036 Taenia solium UNAM-cd2_larva Taenia solium cDNA, mRNA sequence		
EL743299_1 LV0255013 Taenia solium UNAM-cd2_larva Taenia solium cDNA, mRNA sequence		
EL743144_3 LV0252045 Taenia solium UNAM-cd2_larva Taenia solium cDNA, mRNA sequence		
EL743011_1 LV0250052 Taenia solium UNAM-cd2_larva Taenia solium cDNA, mRNA sequence		
EL742977_1 LV0250018 Taenia solium UNAM-cd2_larva Taenia solium cDNA, mRNA sequence		
EL742945_1 LV0249060 Taenia solium UNAM-cd2_larva Taenia solium cDNA, mRNA sequence		
EL742822_1 LV0247040 Taenia solium UNAM-cd2_larva Taenia solium cDNA, mRNA sequence		
EL742764_1 LV0246047 Taenia solium UNAM-cd2_larva Taenia solium cDNA, mRNA sequence		
EL742693_1 LV0245048 Taenia solium UNAM-cd2_larva Taenia solium cDNA, mRNA sequence		
EL742310_1 LV0240005 Taenia solium UNAM-cd2_larva Taenia solium cDNA, mRNA sequence		
EL742275_3 LV0239027 Taenia solium UNAM-cd2_larva Taenia solium cDNA, mRNA sequence		
EL742272_2 LV0239024 Taenia solium UNAM-cd2_larva Taenia solium cDNA, mRNA sequence		
EL742219_1 LV0238038 Taenia solium UNAM-cd2_larva Taenia solium cDNA, mRNA sequence		
EL741993_1 LV0234054 Taenia solium UNAM-cd2_larva Taenia solium cDNA, mRNA sequence		
EL741664_1 LV0230011 Taenia solium UNAM-cd2_larva Taenia solium cDNA, mRNA sequence		
EL741530_1 LV0228012 Taenia solium UNAM-cd2_larva Taenia solium cDNA, mRNA sequence		
EL741490_1 LV0227046 Taenia solium UNAM-cd2_larva Taenia solium cDNA, mRNA sequence		
EL741350_1 LV0225001 Taenia solium UNAM-cd2_larva Taenia solium cDNA, mRNA sequence		
EL741331_2 LV0224051 Taenia solium UNAM-cd2_larva Taenia solium cDNA, mRNA sequence		
EL741327_1 LV0224047 Taenia solium UNAM-cd2_larva Taenia solium cDNA, mRNA sequence		
EL741079_1 LV0220052 Taenia solium UNAM-cd2_larva Taenia solium cDNA, mRNA sequence		
EL741016_1 LV0219066 Taenia solium UNAM-cd2_larva Taenia solium cDNA, mRNA sequence		
EL741013_1 LV0219063 Taenia solium UNAM-cd2_larva Taenia solium cDNA, mRNA sequence		
EL740974_1 LV0219024 Taenia solium UNAM-cd2_larva Taenia solium cDNA, mRNA sequence		
EL740962_1 LV0219012 Taenia solium UNAM-cd2_larva Taenia solium cDNA, mRNA sequence		
EL740711_2 LV0215001 Taenia solium UNAM-cd2_larva Taenia solium cDNA, mRNA sequence		
EL740699_2 LV0214024 Taenia solium UNAM-cd2_larva Taenia solium cDNA, mRNA sequence		
EL740680_1 LV0214005 Taenia solium UNAM-cd2_larva Taenia solium cDNA, mRNA sequence		
EL740467_1 LV0209045 Taenia solium UNAM-cd2_larva Taenia solium cDNA, mRNA sequence		
EL740365_1 LV0207049 Taenia solium UNAM-cd2_larva Taenia solium cDNA, mRNA sequence		
EL740307_1 LV0206039 Taenia solium UNAM-cd2_larva Taenia solium cDNA, mRNA sequence		
EL740283_1 LV0206015 Taenia solium UNAM-cd2_larva Taenia solium cDNA, mRNA sequence		
>gi|22657791|gb|AAD51767.2|AF098073_1 18 kDa glycoprotein TS18 variant 1 precursor [Taenia solium]	found 121 times	93
EL748174_1 LV0334057 Taenia solium UNAM-cd2_larva Taenia solium cDNA, mRNA sequence		
EL748119_1 LV0334002 Taenia solium UNAM-cd2_larva Taenia solium cDNA, mRNA sequence		
EL748076_1 LV0333027 Taenia solium UNAM-cd2_larva Taenia solium cDNA, mRNA sequence		
EL748064_1 LV0333015 Taenia solium UNAM-cd2_larva Taenia solium cDNA, mRNA sequence		
EL747983_1 LV0332005 Taenia solium UNAM-cd2_larva Taenia solium cDNA, mRNA sequence		
EL747935_1 LV0331028 Taenia solium UNAM-cd2_larva Taenia solium cDNA, mRNA sequence		
EL747932_1 LV0331025 Taenia solium UNAM-cd2_larva Taenia solium cDNA, mRNA sequence		
EL747834_1 LV0329072 Taenia solium UNAM-cd2_larva Taenia solium cDNA, mRNA sequence		
EL747779_1 LV0329017 Taenia solium UNAM-cd2_larva Taenia solium cDNA, mRNA sequence		
EL747762_1 LV0328050 Taenia solium UNAM-cd2_larva Taenia solium cDNA, mRNA sequence		
EL747686_1 LV0327029 Taenia solium UNAM-cd2_larva Taenia solium cDNA, mRNA sequence		
EL747553_1 LV0325032 Taenia solium UNAM-cd2_larva Taenia solium cDNA, mRNA sequence		
EL747544_4 LV0325023 Taenia solium UNAM-cd2_larva Taenia solium cDNA, mRNA sequence		
EL747502_1 LV0324038 Taenia solium UNAM-cd2_larva Taenia solium cDNA, mRNA sequence		
EL747441_3 LV0323050 Taenia solium UNAM-cd2_larva Taenia solium cDNA, mRNA sequence		
EL747400_1 LV0323009 Taenia solium UNAM-cd2_larva Taenia solium cDNA, mRNA sequence		
EL747398_1 LV0323007 Taenia solium UNAM-cd2_larva Taenia solium cDNA, mRNA sequence		
EL747372_1 LV0322049 Taenia solium UNAM-cd2_larva Taenia solium cDNA, mRNA sequence		
EL747322_1 LV0321053 Taenia solium UNAM-cd2_larva Taenia solium cDNA, mRNA sequence		
EL747267_1 LV0320064 Taenia solium UNAM-cd2_larva Taenia solium cDNA, mRNA sequence		
EL747227_1 LV0320024 Taenia solium UNAM-cd2_larva Taenia solium cDNA, mRNA sequence		
EL747210_1 LV0320007 Taenia solium UNAM-cd2_larva Taenia solium cDNA, mRNA sequence		

Table 1: Continued.

**Protein representing protein group**<br>>Protein identified by ESTs<br>EST	number of ESTs linked to protein	TBLASTN score
EL747118_1 LV0318050 Taenia solium UNAM-cd2_larva Taenia solium cDNA, mRNA sequence		
EL747031_1 LV0317028 Taenia solium UNAM-cd2_larva Taenia solium cDNA, mRNA sequence		
EL746959_2 LV0316036 Taenia solium UNAM-cd2_larva Taenia solium cDNA, mRNA sequence		
EL746932_1 LV0316009 Taenia solium UNAM-cd2_larva Taenia solium cDNA, mRNA sequence		
EL746866_3 LV0315008 Taenia solium UNAM-cd2_larva Taenia solium cDNA, mRNA sequence		
EL746789_1 LV0314002 Taenia solium UNAM-cd2_larva Taenia solium cDNA, mRNA sequence		
EL746759_1 LV0313042 Taenia solium UNAM-cd2_larva Taenia solium cDNA, mRNA sequence		
EL746689_1 LV0312050 Taenia solium UNAM-cd2_larva Taenia solium cDNA, mRNA sequence		
EL746564_1 LV0310061 Taenia solium UNAM-cd2_larva Taenia solium cDNA, mRNA sequence		
EL746490_1 LV0309060 Taenia solium UNAM-cd2_larva Taenia solium cDNA, mRNA sequence		
EL746478_1 LV0309048 Taenia solium UNAM-cd2_larva Taenia solium cDNA, mRNA sequence		
EL746390_1 LV0308040 Taenia solium UNAM-cd2_larva Taenia solium cDNA, mRNA sequence		
EL746253_3 LV0306004 Taenia solium UNAM-cd2_larva Taenia solium cDNA, mRNA sequence		
EL746194_1 LV0304019 Taenia solium UNAM-cd2_larva Taenia solium cDNA, mRNA sequence		
EL746183_3 LV0304008 Taenia solium UNAM-cd2_larva Taenia solium cDNA, mRNA sequence		
EL745994_1 LV0300025 Taenia solium UNAM-cd2_larva Taenia solium cDNA, mRNA sequence		
EL745927_2 LV0299016 Taenia solium UNAM-cd2_larva Taenia solium cDNA, mRNA sequence		
EL745844_3 LV0298001 Taenia solium UNAM-cd2_larva Taenia solium cDNA, mRNA sequence		
EL745794_1 LV0297023 Taenia solium UNAM-cd2_larva Taenia solium cDNA, mRNA sequence		
EL745785_1 LV0297014 Taenia solium UNAM-cd2_larva Taenia solium cDNA, mRNA sequence		
EL745781_1 LV0297010 Taenia solium UNAM-cd2_larva Taenia solium cDNA, mRNA sequence		
EL745764_1 LV0296054 Taenia solium UNAM-cd2_larva Taenia solium cDNA, mRNA sequence		
EL745756_1 LV0296046 Taenia solium UNAM-cd2_larva Taenia solium cDNA, mRNA sequence		
EL745753_1 LV0296043 Taenia solium UNAM-cd2_larva Taenia solium cDNA, mRNA sequence		
EL745679_1 LV0295037 Taenia solium UNAM-cd2_larva Taenia solium cDNA, mRNA sequence		
EL745537_1 LV0293015 Taenia solium UNAM-cd2_larva Taenia solium cDNA, mRNA sequence		
EL745522_1 LV0292056 Taenia solium UNAM-cd2_larva Taenia solium cDNA, mRNA sequence		
EL745496_1 LV0292030 Taenia solium UNAM-cd2_larva Taenia solium cDNA, mRNA sequence		
EL745371_4 LV0290026 Taenia solium UNAM-cd2_larva Taenia solium cDNA, mRNA sequence		
EL745365_1 LV0290020 Taenia solium UNAM-cd2_larva Taenia solium cDNA, mRNA sequence		
EL745116_1 LV0286033 Taenia solium UNAM-cd2_larva Taenia solium cDNA, mRNA sequence		
EL745093_1 LV0286009 Taenia solium UNAM-cd2_larva Taenia solium cDNA, mRNA sequence		
EL745077_1 LV0285049 Taenia solium UNAM-cd2_larva Taenia solium cDNA, mRNA sequence		
EL745017_1 LV0284048 Taenia solium UNAM-cd2_larva Taenia solium cDNA, mRNA sequence		
EL744937_1 LV0283001 Taenia solium UNAM-cd2_larva Taenia solium cDNA, mRNA sequence		
EL744884_2 LV0282010 Taenia solium UNAM-cd2_larva Taenia solium cDNA, mRNA sequence		
EL744773_3 LV0279042 Taenia solium UNAM-cd2_larva Taenia solium cDNA, mRNA sequence		
EL744703_1 LV0278022 Taenia solium UNAM-cd2_larva Taenia solium cDNA, mRNA sequence		
EL744642_5 LV0277019 Taenia solium UNAM-cd2_larva Taenia solium cDNA, mRNA sequence		
EL744597_1 LV0276015 Taenia solium UNAM-cd2_larva Taenia solium cDNA, mRNA sequence		
EL744454_1 LV0273012 Taenia solium UNAM-cd2_larva Taenia solium cDNA, mRNA sequence		
EL744442_1 LV0272057 Taenia solium UNAM-cd2_larva Taenia solium cDNA, mRNA sequence		
EL744374_1 LV0271056 Taenia solium UNAM-cd2_larva Taenia solium cDNA, mRNA sequence		
EL744354_1 LV0271036 Taenia solium UNAM-cd2_larva Taenia solium cDNA, mRNA sequence		
EL744313_1 LV0270060 Taenia solium UNAM-cd2_larva Taenia solium cDNA, mRNA sequence		
EL744302_1 LV0270049 Taenia solium UNAM-cd2_larva Taenia solium cDNA, mRNA sequence		
EL744219_1 LV0269030 Taenia solium UNAM-cd2_larva Taenia solium cDNA, mRNA sequence		
EL744201_1 LV0269012 Taenia solium UNAM-cd2_larva Taenia solium cDNA, mRNA sequence		
EL744140_1 LV0268007 Taenia solium UNAM-cd2_larva Taenia solium cDNA, mRNA sequence		
EL744099_1 LV0267038 Taenia solium UNAM-cd2_larva Taenia solium cDNA, mRNA sequence		
EL744068_3 LV0267007 Taenia solium UNAM-cd2_larva Taenia solium cDNA, mRNA sequence		
EL743949_1 LV0265021 Taenia solium UNAM-cd2_larva Taenia solium cDNA, mRNA sequence		
EL743944_2 LV0265016 Taenia solium UNAM-cd2_larva Taenia solium cDNA, mRNA sequence		
EL743942_1 LV0265014 Taenia solium UNAM-cd2_larva Taenia solium cDNA, mRNA sequence		
EL743925_1 LV0264054 Taenia solium UNAM-cd2_larva Taenia solium cDNA, mRNA sequence		
EL743864_1 LV0263033 Taenia solium UNAM-cd2_larva Taenia solium cDNA, mRNA sequence		
EL743784_1 LV0262025 Taenia solium UNAM-cd2_larva Taenia solium cDNA, mRNA sequence		
EL743699_4 LV0261008 Taenia solium UNAM-cd2_larva Taenia solium cDNA, mRNA sequence		
EL743583_2 LV0259046 Taenia solium UNAM-cd2_larva Taenia solium cDNA, mRNA sequence		
EL743524_1 LV0258064 Taenia solium UNAM-cd2_larva Taenia solium cDNA, mRNA sequence		
EL743328_1 LV0255042 Taenia solium UNAM-cd2_larva Taenia solium cDNA, mRNA sequence		
EL743241_1 LV0254022 Taenia solium UNAM-cd2_larva Taenia solium cDNA, mRNA sequence		
EL743228_1 LV0254009 Taenia solium UNAM-cd2_larva Taenia solium cDNA, mRNA sequence		
EL743227_1 LV0254008 Taenia solium UNAM-cd2_larva Taenia solium cDNA, mRNA sequence		
EL743107_1 LV0252008 Taenia solium UNAM-cd2_larva Taenia solium cDNA, mRNA sequence		
EL742946_1 LV0249061 Taenia solium UNAM-cd2_larva Taenia solium cDNA, mRNA sequence		
EL742846_1 LV0248008 Taenia solium UNAM-cd2_larva Taenia solium cDNA, mRNA sequence		
EL742802_1 LV0247020 Taenia solium UNAM-cd2_larva Taenia solium cDNA, mRNA sequence		
EL742795_1 LV0247013 Taenia solium UNAM-cd2_larva Taenia solium cDNA, mRNA sequence		
EL742659_1 LV0245014 Taenia solium UNAM-cd2_larva Taenia solium cDNA, mRNA sequence		
EL742637_1 LV0244068 Taenia solium UNAM-cd2_larva Taenia solium cDNA, mRNA sequence		
EL742570_1 LV0244001 Taenia solium UNAM-cd2_larva Taenia solium cDNA, mRNA sequence		
EL742532_1 LV0243030 Taenia solium UNAM-cd2_larva Taenia solium cDNA, mRNA sequence		
EL742529_1 LV0243027 Taenia solium UNAM-cd2_larva Taenia solium cDNA, mRNA sequence		
EL742517_1 LV0243015 Taenia solium UNAM-cd2_larva Taenia solium cDNA, mRNA sequence		
EL742377_1 LV0241003 Taenia solium UNAM-cd2_larva Taenia solium cDNA, mRNA sequence		
EL742338_1 LV0240033 Taenia solium UNAM-cd2_larva Taenia solium cDNA, mRNA sequence		
EL742332_1 LV0240027 Taenia solium UNAM-cd2_larva Taenia solium cDNA, mRNA sequence		
EL742119_1 LV0237007 Taenia solium UNAM-cd2_larva Taenia solium cDNA, mRNA sequence		
EL742004_1 LV0234065 Taenia solium UNAM-cd2_larva Taenia solium cDNA, mRNA sequence		
EL741948_3 LV0234009 Taenia solium UNAM-cd2_larva Taenia solium cDNA, mRNA sequence		
EL741794_1 LV0231072 Taenia solium UNAM-cd2_larva Taenia solium cDNA, mRNA sequence		

Table 1: Continued.

<table>
<tr><th>Protein representing protein group
>Protein identified by ESTs
EST</th><th>number of ESTs linked to protein</th><th>TBLASTN score</th></tr>
<tr><td>EL741736_1 LV0231014 Taenia solium UNAM-cd2_larva Taenia solium cDNA, mRNA sequence</td><td></td><td></td></tr>
<tr><td>EL741574_1 LV0228056 Taenia solium UNAM-cd2_larva Taenia solium cDNA, mRNA sequence</td><td></td><td></td></tr>
<tr><td>EL741547_1 LV0228029 Taenia solium UNAM-cd2_larva Taenia solium cDNA, mRNA sequence</td><td></td><td></td></tr>
<tr><td>EL741524_1 LV0228006 Taenia solium UNAM-cd2_larva Taenia solium cDNA, mRNA sequence</td><td></td><td></td></tr>
<tr><td>EL741466_1 LV0227022 Taenia solium UNAM-cd2_larva Taenia solium cDNA, mRNA sequence</td><td></td><td></td></tr>
<tr><td>EL741396_1 LV0226015 Taenia solium UNAM-cd2_larva Taenia solium cDNA, mRNA sequence</td><td></td><td></td></tr>
<tr><td>EL741389_1 LV0226008 Taenia solium UNAM-cd2_larva Taenia solium cDNA, mRNA sequence</td><td></td><td></td></tr>
<tr><td>EL741361_1 LV0225012 Taenia solium UNAM-cd2_larva Taenia solium cDNA, mRNA sequence</td><td></td><td></td></tr>
<tr><td>EL741259_1 LV0223037 Taenia solium UNAM-cd2_larva Taenia solium cDNA, mRNA sequence</td><td></td><td></td></tr>
<tr><td>EL741089_1 LV0220062 Taenia solium UNAM-cd2_larva Taenia solium cDNA, mRNA sequence</td><td></td><td></td></tr>
<tr><td>EL741039_1 LV0220012 Taenia solium UNAM-cd2_larva Taenia solium cDNA, mRNA sequence</td><td></td><td></td></tr>
<tr><td>EL740991_3 LV0219041 Taenia solium UNAM-cd2_larva Taenia solium cDNA, mRNA sequence</td><td></td><td></td></tr>
<tr><td>EL740640_1 LV0213022 Taenia solium UNAM-cd2_larva Taenia solium cDNA, mRNA sequence</td><td></td><td></td></tr>
<tr><td>EL740552_3 LV0211029 Taenia solium UNAM-cd2_larva Taenia solium cDNA, mRNA sequence</td><td></td><td></td></tr>
<tr><td>EL740475_1 LV0209053 Taenia solium UNAM-cd2_larva Taenia solium cDNA, mRNA sequence</td><td></td><td></td></tr>
<tr><td>EL740377_1 LV0207061 Taenia solium UNAM-cd2_larva Taenia solium cDNA, mRNA sequence</td><td></td><td></td></tr>
<tr><td>EL740281_1 LV0206013 Taenia solium UNAM-cd2_larva Taenia solium cDNA, mRNA sequence</td><td></td><td></td></tr>
<tr><td>>gi|6693838|gb|AAF25005.1|AF158184_1 14-kDa diagnostic antigen [Taenia solium]</td><td>found 61 times</td><td>108</td></tr>
<tr><td>EL748134_1 LV0334017 Taenia solium UNAM-cd2_larva Taenia solium cDNA, mRNA sequence</td><td></td><td></td></tr>
<tr><td>EL747953_3 LV0331046 Taenia solium UNAM-cd2_larva Taenia solium cDNA, mRNA sequence</td><td></td><td></td></tr>
<tr><td>EL747770_2 LV0329008 Taenia solium UNAM-cd2_larva Taenia solium cDNA, mRNA sequence</td><td></td><td></td></tr>
<tr><td>EL747512_3 LV0324048 Taenia solium UNAM-cd2_larva Taenia solium cDNA, mRNA sequence</td><td></td><td></td></tr>
<tr><td>EL747444_2 LV0323053 Taenia solium UNAM-cd2_larva Taenia solium cDNA, mRNA sequence</td><td></td><td></td></tr>
<tr><td>EL747360_3 LV0322037 Taenia solium UNAM-cd2_larva Taenia solium cDNA, mRNA sequence</td><td></td><td></td></tr>
<tr><td>EL747148_1 LV0319007 Taenia solium UNAM-cd2_larva Taenia solium cDNA, mRNA sequence</td><td></td><td></td></tr>
<tr><td>EL747126_2 LV0318058 Taenia solium UNAM-cd2_larva Taenia solium cDNA, mRNA sequence</td><td></td><td></td></tr>
<tr><td>EL747061_1 LV0317058 Taenia solium UNAM-cd2_larva Taenia solium cDNA, mRNA sequence</td><td></td><td></td></tr>
<tr><td>EL746960_1 LV0316037 Taenia solium UNAM-cd2_larva Taenia solium cDNA, mRNA sequence</td><td></td><td></td></tr>
<tr><td>EL746699_3 LV0312060 Taenia solium UNAM-cd2_larva Taenia solium cDNA, mRNA sequence</td><td></td><td></td></tr>
<tr><td>EL746661_1 LV0312022 Taenia solium UNAM-cd2_larva Taenia solium cDNA, mRNA sequence</td><td></td><td></td></tr>
<tr><td>EL746561_1 LV0310058 Taenia solium UNAM-cd2_larva Taenia solium cDNA, mRNA sequence</td><td></td><td></td></tr>
<tr><td>EL746513_2 LV0310010 Taenia solium UNAM-cd2_larva Taenia solium cDNA, mRNA sequence</td><td></td><td></td></tr>
<tr><td>EL746389_2 LV0308039 Taenia solium UNAM-cd2_larva Taenia solium cDNA, mRNA sequence</td><td></td><td></td></tr>
<tr><td>EL746268_1 LV0306019 Taenia solium UNAM-cd2_larva Taenia solium cDNA, mRNA sequence</td><td></td><td></td></tr>
<tr><td>EL746237_2 LV0305021 Taenia solium UNAM-cd2_larva Taenia solium cDNA, mRNA sequence</td><td></td><td></td></tr>
<tr><td>EL746135_3 LV0302030 Taenia solium UNAM-cd2_larva Taenia solium cDNA, mRNA sequence</td><td></td><td></td></tr>
<tr><td>EL746091_2 LV0301046 Taenia solium UNAM-cd2_larva Taenia solium cDNA, mRNA sequence</td><td></td><td></td></tr>
<tr><td>EL746019_3 LV0300050 Taenia solium UNAM-cd2_larva Taenia solium cDNA, mRNA sequence</td><td></td><td></td></tr>
<tr><td>EL745684_1 LV0295042 Taenia solium UNAM-cd2_larva Taenia solium cDNA, mRNA sequence</td><td></td><td></td></tr>
<tr><td>EL745486_1 LV0292020 Taenia solium UNAM-cd2_larva Taenia solium cDNA, mRNA sequence</td><td></td><td></td></tr>
<tr><td>EL745357_3 LV0290012 Taenia solium UNAM-cd2_larva Taenia solium cDNA, mRNA sequence</td><td></td><td></td></tr>
<tr><td>EL745215_3 LV0287069 Taenia solium UNAM-cd2_larva Taenia solium cDNA, mRNA sequence</td><td></td><td></td></tr>
<tr><td>EL745039_3 LV0285011 Taenia solium UNAM-cd2_larva Taenia solium cDNA, mRNA sequence</td><td></td><td></td></tr>
<tr><td>EL744915_1 LV0282041 Taenia solium UNAM-cd2_larva Taenia solium cDNA, mRNA sequence</td><td></td><td></td></tr>
<tr><td>EL744852_2 LV0281012 Taenia solium UNAM-cd2_larva Taenia solium cDNA, mRNA sequence</td><td></td><td></td></tr>
<tr><td>EL744728_2 LV0278047 Taenia solium UNAM-cd2_larva Taenia solium cDNA, mRNA sequence</td><td></td><td></td></tr>
<tr><td>EL744699_1 LV0278018 Taenia solium UNAM-cd2_larva Taenia solium cDNA, mRNA sequence</td><td></td><td></td></tr>
<tr><td>EL744602_1 LV0276020 Taenia solium UNAM-cd2_larva Taenia solium cDNA, mRNA sequence</td><td></td><td></td></tr>
<tr><td>EL744575_3 LV0275050 Taenia solium UNAM-cd2_larva Taenia solium cDNA, mRNA sequence</td><td></td><td></td></tr>
<tr><td>EL744491_3 LV0273049 Taenia solium UNAM-cd2_larva Taenia solium cDNA, mRNA sequence</td><td></td><td></td></tr>
<tr><td>EL744466_2 LV0273024 Taenia solium UNAM-cd2_larva Taenia solium cDNA, mRNA sequence</td><td></td><td></td></tr>
<tr><td>EL744436_1 LV0272051 Taenia solium UNAM-cd2_larva Taenia solium cDNA, mRNA sequence</td><td></td><td></td></tr>
<tr><td>EL743813_2 LV0262054 Taenia solium UNAM-cd2_larva Taenia solium cDNA, mRNA sequence</td><td></td><td></td></tr>
<tr><td>EL743542_2 LV0259005 Taenia solium UNAM-cd2_larva Taenia solium cDNA, mRNA sequence</td><td></td><td></td></tr>
<tr><td>EL743456_2 LV0257073 Taenia solium UNAM-cd2_larva Taenia solium cDNA, mRNA sequence</td><td></td><td></td></tr>
<tr><td>EL743413_3 LV0257030 Taenia solium UNAM-cd2_larva Taenia solium cDNA, mRNA sequence</td><td></td><td></td></tr>
<tr><td>EL743101_1 LV0252002 Taenia solium UNAM-cd2_larva Taenia solium cDNA, mRNA sequence</td><td></td><td></td></tr>
<tr><td>EL743003_3 LV0250044 Taenia solium UNAM-cd2_larva Taenia solium cDNA, mRNA sequence</td><td></td><td></td></tr>
<tr><td>EL742992_1 LV0250033 Taenia solium UNAM-cd2_larva Taenia solium cDNA, mRNA sequence</td><td></td><td></td></tr>
<tr><td>EL742559_1 LV0243057 Taenia solium UNAM-cd2_larva Taenia solium cDNA, mRNA sequence</td><td></td><td></td></tr>
<tr><td>EL742440_3 LV0242004 Taenia solium UNAM-cd2_larva Taenia solium cDNA, mRNA sequence</td><td></td><td></td></tr>
<tr><td>EL742384_3 LV0241010 Taenia solium UNAM-cd2_larva Taenia solium cDNA, mRNA sequence</td><td></td><td></td></tr>
<tr><td>EL742345_2 LV0240040 Taenia solium UNAM-cd2_larva Taenia solium cDNA, mRNA sequence</td><td></td><td></td></tr>
<tr><td>EL742331_1 LV0240026 Taenia solium UNAM-cd2_larva Taenia solium cDNA, mRNA sequence</td><td></td><td></td></tr>
<tr><td>EL742267_1 LV0239019 Taenia solium UNAM-cd2_larva Taenia solium cDNA, mRNA sequence</td><td></td><td></td></tr>
<tr><td>EL741839_1 LV0232044 Taenia solium UNAM-cd2_larva Taenia solium cDNA, mRNA sequence</td><td></td><td></td></tr>
<tr><td>EL741614_2 LV0229025 Taenia solium UNAM-cd2_larva Taenia solium cDNA, mRNA sequence</td><td></td><td></td></tr>
<tr><td>EL741611_1 LV0229022 Taenia solium UNAM-cd2_larva Taenia solium cDNA, mRNA sequence</td><td></td><td></td></tr>
<tr><td>EL741523_3 LV0228005 Taenia solium UNAM-cd2_larva Taenia solium cDNA, mRNA sequence</td><td></td><td></td></tr>
<tr><td>EL741349_2 LV0224069 Taenia solium UNAM-cd2_larva Taenia solium cDNA, mRNA sequence</td><td></td><td></td></tr>
<tr><td>EL741266_3 LV0223044 Taenia solium UNAM-cd2_larva Taenia solium cDNA, mRNA sequence</td><td></td><td></td></tr>
<tr><td>EL741223_2 LV0223001 Taenia solium UNAM-cd2_larva Taenia solium cDNA, mRNA sequence</td><td></td><td></td></tr>
<tr><td>EL741138_3 LV0221039 Taenia solium UNAM-cd2_larva Taenia solium cDNA, mRNA sequence</td><td></td><td></td></tr>
<tr><td>EL741122_3 LV0221023 Taenia solium UNAM-cd2_larva Taenia solium cDNA, mRNA sequence</td><td></td><td></td></tr>
<tr><td>EL740965_1 LV0219015 Taenia solium UNAM-cd2_larva Taenia solium cDNA, mRNA sequence</td><td></td><td></td></tr>
<tr><td>EL740796_1 LV0216030 Taenia solium UNAM-cd2_larva Taenia solium cDNA, mRNA sequence</td><td></td><td></td></tr>
<tr><td>EL740753_2 LV0215043 Taenia solium UNAM-cd2_larva Taenia solium cDNA, mRNA sequence</td><td></td><td></td></tr>
<tr><td>EL740549_3 LV0211026 Taenia solium UNAM-cd2_larva Taenia solium cDNA, mRNA sequence</td><td></td><td></td></tr>
<tr><td>EL740384_1 LV0208004 Taenia solium UNAM-cd2_larva Taenia solium cDNA, mRNA sequence</td><td></td><td></td></tr>
<tr><td>>gi|7339851|gb|AAF60975.1| immunogenic protein Ts21 [Taenia solium]</td><td>found 1 time</td><td>138</td></tr>
<tr><td>EL745919_1 LV0299008 Taenia solium UNAM-cd2_larva Taenia solium cDNA, mRNA sequence</td><td></td><td></td></tr>
<tr><td>>gi|19879954|gb|AAM00209.1|AF356340_1 8 kDa diagnostic antigen TsRS1 variant 1 [Taenia solium]</td><td>found 1 time</td><td>138</td></tr>
</table>

Table 1: Continued.

**Protein representing protein group**<br>>Protein identified by ESTs<br>EST	number of ESTs linked to protein	TBLASTN score
EL747386_3 LV0322063 Taenia solium UNAM-cd2_larva Taenia solium cDNA, mRNA sequence		
>gi|23477222|emb|CAD48845.1| secreted antigen Ts8B1 [Taenia solium]	found 67 times	186
GT890297_2 TSAY.R21.esd Taenia solium cysticerca cDNA library Taenia solium cDNA, mRNA sequence		
EL762598_2 AD0189055 Taenia solium UNAM-cd1_adult Taenia solium cDNA, mRNA sequence		
EL761784_5 AD0171001 Taenia solium UNAM-cd1_adult Taenia solium cDNA, mRNA sequence		
EL761404_4 AD0163013 Taenia solium UNAM-cd1_adult Taenia solium cDNA, mRNA sequence		
EL759588_2 AD0125046 Taenia solium UNAM-cd1_adult Taenia solium cDNA, mRNA sequence		
EL758887_2 AD0113028 Taenia solium UNAM-cd1_adult Taenia solium cDNA, mRNA sequence		
EL758833_1 AD0112032 Taenia solium UNAM-cd1_adult Taenia solium cDNA, mRNA sequence		
EL758619_1 AD0108050 Taenia solium UNAM-cd1_adult Taenia solium cDNA, mRNA sequence		
EL758542_1 AD0107035 Taenia solium UNAM-cd1_adult Taenia solium cDNA, mRNA sequence		
EL758030_2 AD0099042 Taenia solium UNAM-cd1_adult Taenia solium cDNA, mRNA sequence		
EL757964_1 AD0098049 Taenia solium UNAM-cd1_adult Taenia solium cDNA, mRNA sequence		
EL757509_2 AD0035102 Taenia solium UNAM-cd1_adult Taenia solium cDNA, mRNA sequence		
EL757095_1 AD0032107 Taenia solium UNAM-cd1_adult Taenia solium cDNA, mRNA sequence		
EL756871_2 AD0031144 Taenia solium UNAM-cd1_adult Taenia solium cDNA, mRNA sequence		
EL756796_3 AD0031069 Taenia solium UNAM-cd1_adult Taenia solium cDNA, mRNA sequence		
EL756311_1 AD0029106 Taenia solium UNAM-cd1_adult Taenia solium cDNA, mRNA sequence		
EL756272_3 AD0029067 Taenia solium UNAM-cd1_adult Taenia solium cDNA, mRNA sequence		
EL755046_2 AD0024063 Taenia solium UNAM-cd1_adult Taenia solium cDNA, mRNA sequence		
EL754878_2 AD0023134 Taenia solium UNAM-cd1_adult Taenia solium cDNA, mRNA sequence		
EL754151_2 AD0020196 Taenia solium UNAM-cd1_adult Taenia solium cDNA, mRNA sequence		
EL753916_2 AD0019203 Taenia solium UNAM-cd1_adult Taenia solium cDNA, mRNA sequence		
EL751498_4 AD0009236 Taenia solium UNAM-cd1_adult Taenia solium cDNA, mRNA sequence		
EL751252_3 AD0008263 Taenia solium UNAM-cd1_adult Taenia solium cDNA, mRNA sequence		
EL750541_4 AD0005204 Taenia solium UNAM-cd1_adult Taenia solium cDNA, mRNA sequence		
EL750312_2 AD0004227 Taenia solium UNAM-cd1_adult Taenia solium cDNA, mRNA sequence		
EL749377_3 LV0286023 Taenia solium UNAM-cd2_larva Taenia solium cDNA, mRNA sequence		
EL749246_3 LV0356024 Taenia solium UNAM-cd2_larva Taenia solium cDNA, mRNA sequence		
EL748735_2 LV0344058 Taenia solium UNAM-cd2_larva Taenia solium cDNA, mRNA sequence		
EL748498_1 LV0341014 Taenia solium UNAM-cd2_larva Taenia solium cDNA, mRNA sequence		
EL748028_3 LV0332050 Taenia solium UNAM-cd2_larva Taenia solium cDNA, mRNA sequence		
EL747972_1 LV0331065 Taenia solium UNAM-cd2_larva Taenia solium cDNA, mRNA sequence		
EL747721_2 LV0328009 Taenia solium UNAM-cd2_larva Taenia solium cDNA, mRNA sequence		
EL747135_2 LV0318067 Taenia solium UNAM-cd2_larva Taenia solium cDNA, mRNA sequence		
EL747119_1 LV0318051 Taenia solium UNAM-cd2_larva Taenia solium cDNA, mRNA sequence		
EL746913_2 LV0315055 Taenia solium UNAM-cd2_larva Taenia solium cDNA, mRNA sequence		
EL746192_1 LV0304017 Taenia solium UNAM-cd2_larva Taenia solium cDNA, mRNA sequence		
EL746178_2 LV0304003 Taenia solium UNAM-cd2_larva Taenia solium cDNA, mRNA sequence		
EL746012_3 LV0300043 Taenia solium UNAM-cd2_larva Taenia solium cDNA, mRNA sequence		
EL745968_3 LV0299057 Taenia solium UNAM-cd2_larva Taenia solium cDNA, mRNA sequence		
EL745951_1 LV0299040 Taenia solium UNAM-cd2_larva Taenia solium cDNA, mRNA sequence		
EL745883_3 LV0298040 Taenia solium UNAM-cd2_larva Taenia solium cDNA, mRNA sequence		
EL745749_3 LV0296039 Taenia solium UNAM-cd2_larva Taenia solium cDNA, mRNA sequence		
EL745656_2 LV0295014 Taenia solium UNAM-cd2_larva Taenia solium cDNA, mRNA sequence		
EL745181_3 LV0287035 Taenia solium UNAM-cd2_larva Taenia solium cDNA, mRNA sequence		
EL744867_3 LV0281027 Taenia solium UNAM-cd2_larva Taenia solium cDNA, mRNA sequence		
EL744512_1 LV0274015 Taenia solium UNAM-cd2_larva Taenia solium cDNA, mRNA sequence		
EL744380_3 LV0271062 Taenia solium UNAM-cd2_larva Taenia solium cDNA, mRNA sequence		
EL744192_2 LV0269003 Taenia solium UNAM-cd2_larva Taenia solium cDNA, mRNA sequence		
EL744046_2 LV0266046 Taenia solium UNAM-cd2_larva Taenia solium cDNA, mRNA sequence		
EL743756_1 LV0261065 Taenia solium UNAM-cd2_larva Taenia solium cDNA, mRNA sequence		
EL743659_3 LV0260047 Taenia solium UNAM-cd2_larva Taenia solium cDNA, mRNA sequence		
EL743648_3 LV0260036 Taenia solium UNAM-cd2_larva Taenia solium cDNA, mRNA sequence		
EL743067_3 LV0251032 Taenia solium UNAM-cd2_larva Taenia solium cDNA, mRNA sequence		
EL742126_2 LV0237014 Taenia solium UNAM-cd2_larva Taenia solium cDNA, mRNA sequence		
EL742094_3 LV0236023 Taenia solium UNAM-cd2_larva Taenia solium cDNA, mRNA sequence		
EL742046_2 LV0235035 Taenia solium UNAM-cd2_larva Taenia solium cDNA, mRNA sequence		
EL741814_3 LV0232019 Taenia solium UNAM-cd2_larva Taenia solium cDNA, mRNA sequence		
EL741449_2 LV0227005 Taenia solium UNAM-cd2_larva Taenia solium cDNA, mRNA sequence		
EL741410_1 LV0226029 Taenia solium UNAM-cd2_larva Taenia solium cDNA, mRNA sequence		
EL741373_3 LV0225024 Taenia solium UNAM-cd2_larva Taenia solium cDNA, mRNA sequence		
EL741066_3 LV0220039 Taenia solium UNAM-cd2_larva Taenia solium cDNA, mRNA sequence		
EL740931_3 LV0218042 Taenia solium UNAM-cd2_larva Taenia solium cDNA, mRNA sequence		
EL740877_1 LV0217060 Taenia solium UNAM-cd2_larva Taenia solium cDNA, mRNA sequence		
EL740472_2 LV0209050 Taenia solium UNAM-cd2_larva Taenia solium cDNA, mRNA sequence		
EL740364_1 LV0207048 Taenia solium UNAM-cd2_larva Taenia solium cDNA, mRNA sequence		
EL740290_3 LV0206022 Taenia solium UNAM-cd2_larva Taenia solium cDNA, mRNA sequence		
EL740239_1 LV0205019 Taenia solium UNAM-cd2_larva Taenia solium cDNA, mRNA sequence		
**Peroxiredoxin**		
>gi|2352262|gb|AAB68798.1| peroxidoxin-1 [Dirofilaria immitis]	found 1 time	528
EL749304_1 LV0358015 Taenia solium UNAM-cd2_larva Taenia solium cDNA, mRNA sequence		
>gi|285803079|gb|ADC35419.1| peroxiredoxin [Pinctada fucata]	found 3 times	531
EL748006_4 LV0332028 Taenia solium UNAM-cd2_larva Taenia solium cDNA, mRNA sequence		
EL744872_3 LV0281032 Taenia solium UNAM-cd2_larva Taenia solium cDNA, mRNA sequence		
EL742509_4 LV0243007 Taenia solium UNAM-cd2_larva Taenia solium cDNA, mRNA sequence		
>gi|306451460|gb|ADM88874.1| thioredoxin peroxidase [Cristaria plicata]	found 1 time	534
EL761462_6 AD0164018 Taenia solium UNAM-cd1_adult Taenia solium cDNA, mRNA sequence		
>gi|341616326|gb|AEK86200.1| peroxiredoxin 3 [Clonorchis sinensis]	found 6 times	502
EL762851_3 AD0194022 Taenia solium UNAM-cd1_adult Taenia solium cDNA, mRNA sequence		
EL755161_1 AD0024178 Taenia solium UNAM-cd1_adult Taenia solium cDNA, mRNA sequence		

**Protein representing protein group**<br>>Protein identified by ESTs<br>EST	number of ESTs linked to protein	TBLASTN score
EL752077_3 AD0012105 Taenia solium UNAM-cd1_adult Taenia solium cDNA, mRNA sequence		
EL750776_6 AD0007012 Taenia solium UNAM-cd1_adult Taenia solium cDNA, mRNA sequence		
EL744433_3 LV0272048 Taenia solium UNAM-cd2_larva Taenia solium cDNA, mRNA sequence		
EL743224_3 LV0254005 Taenia solium UNAM-cd2_larva Taenia solium cDNA, mRNA sequence		
>gi|223403612|gb|ACM89281.1| 2-cys peroxiredoxin [Taenia solium]	found 16 times	742
EL761530_4 AD0165028 Taenia solium UNAM-cd1_adult Taenia solium cDNA, mRNA sequence		
EL760164_3 AD0136055 Taenia solium UNAM-cd1_adult Taenia solium cDNA, mRNA sequence		
EL759142_3 AD0117045 Taenia solium UNAM-cd1_adult Taenia solium cDNA, mRNA sequence		
EL756278_1 AD0029073 Taenia solium UNAM-cd1_adult Taenia solium cDNA, mRNA sequence		
EL755296_3 AD0025066 Taenia solium UNAM-cd1_adult Taenia solium cDNA, mRNA sequence		
EL754941_1 AD0023197 Taenia solium UNAM-cd1_adult Taenia solium cDNA, mRNA sequence		
EL752791_4 AD0015050 Taenia solium UNAM-cd1_adult Taenia solium cDNA, mRNA sequence		
EL752524_1 AD0014065 Taenia solium UNAM-cd1_adult Taenia solium cDNA, mRNA sequence		
EL752281_6 AD0013048 Taenia solium UNAM-cd1_adult Taenia solium cDNA, mRNA sequence		
EL752027_3 AD0012055 Taenia solium UNAM-cd1_adult Taenia solium cDNA, mRNA sequence		
EL749234_1 LV0356012 Taenia solium UNAM-cd2_larva Taenia solium cDNA, mRNA sequence		
EL748917_1 LV0348028 Taenia solium UNAM-cd2_larva Taenia solium cDNA, mRNA sequence		
EL747402_3 LV0323011 Taenia solium UNAM-cd2_larva Taenia solium cDNA, mRNA sequence		
EL746637_1 LV0311066 Taenia solium UNAM-cd2_larva Taenia solium cDNA, mRNA sequence		
EL745423_1 LV0291015 Taenia solium UNAM-cd2_larva Taenia solium cDNA, mRNA sequence		
EL741813_1 LV0232018 Taenia solium UNAM-cd2_larva Taenia solium cDNA, mRNA sequence		
>gi|321172106|gb|ADW77118.1| thioredoxin peroxidase [Taenia multiceps]	found 4 times	751
EL762228_6 AD0181025 Taenia solium UNAM-cd1_adult Taenia solium cDNA, mRNA sequence		
EL761887_6 AD0173008 Taenia solium UNAM-cd1_adult Taenia solium cDNA, mRNA sequence		
EL752861_4 AD0015120 Taenia solium UNAM-cd1_adult Taenia solium cDNA, mRNA sequence		
EL752610_1 AD0014151 Taenia solium UNAM-cd1_adult Taenia solium cDNA, mRNA sequence		
**Heat shock protein 90**		
>gi|124783236|gb|ABN14913.1| heat shock protein 90 [Taenia asiatica]	found 3 times	1139
GT227670_3 tscaa0_001923.z1.scf Taenia solium adult full-length cDNA library Taenia solium cDNA clone tscaa0_001923.z1.scf 5', mRNA sequence		
EL754494_1 AD0022019 Taenia solium UNAM-cd1_adult Taenia solium cDNA, mRNA sequence		
EL742305_1 LV0239057 Taenia solium UNAM-cd2_larva Taenia solium cDNA, mRNA sequence		
>gi|300390615|gb|ADK11101.1| heat shock protein 90, partial [Ruditapes philippinarum]	found 1 time	843
EL752024_1 AD0012052 Taenia solium UNAM-cd1_adult Taenia solium cDNA, mRNA sequence		
>gi|398925693|ref|ZP_10662040.1| molecular chaperone of HSP90 family [Pseudomonas sp. GM48]	found 1 time	748
EX150837_3 TSEDTS1022D11 Cysti Taenia solium cDNA, mRNA sequence		
>gi|124783119|gb|ABN14906.1| heat shock protein 90 alpha [Taenia asiatica]	found 2 times	763
GT227126_2 tscaa0_000940.z1.scf Taenia solium adult full-length cDNA library Taenia solium cDNA clone tscaa0_000940.z1.scf 5', mRNA sequence		
EL758995_3 AD0115020 Taenia solium UNAM-cd1_adult Taenia solium cDNA, mRNA sequence		
**Actin**		
>gi|543766|sp|P35432.1|ACT1_ECHGR RecName: Full=Actin-1	found 1 time	1944
GT227121_1 tscaa0_000189.z1.scf Taenia solium adult full-length cDNA library Taenia solium cDNA clone tscaa0_000189.z1.scf 5', mRNA sequence		
>gi|60832848|gb|AAX37027.1| actin alpha 1 [synthetic construct]	found 1 time	1781
sp|P68137|ACTS_PIG Actin, alpha skeletal muscle OS=Sus scrofa GN=ACTA1 PE=3 SV=1		
>gi|89574414|gb|ABD77423.1| cytoplasmic beta-actin [Alligator mississippiensis]	found 3 times	766
EL758802_3 AD0112001 Taenia solium UNAM-cd1_adult Taenia solium cDNA, mRNA sequence		
EL758456_2 AD0105075 Taenia solium UNAM-cd1_adult Taenia solium cDNA, mRNA sequence		
EL758044_2 AD0099056 Taenia solium UNAM-cd1_adult Taenia solium cDNA, mRNA sequence		
>gi|89276723|gb|ABD66582.1| beta-actin [Homo sapiens]	found 3 times	758
EL757580_1 AD0035173 Taenia solium UNAM-cd1_adult Taenia solium cDNA, mRNA sequence		
EL754670_2 AD0022195 Taenia solium UNAM-cd1_adult Taenia solium cDNA, mRNA sequence		
EL753071_3 AD0016109 Taenia solium UNAM-cd1_adult Taenia solium cDNA, mRNA sequence		
>gi|195143563|ref|XP_002012767.1| GL23756 [Drosophila persimilis]	found 1 time	1503
GT890557_1 TSAD.R87.esd Taenia solium cysticerca cDNA library Taenia solium cDNA, mRNA sequence		
>gi|374719223|gb|AEZ67297.1| ACTB, partial [Hipposideros caffer]	found 1 time	572
GT891563_3 TSBQ.R29.esd Taenia solium cysticerca cDNA library Taenia solium cDNA, mRNA sequence		
**Ubiquitin**		
>gi|89891990|gb|ABD78847.1| ubiquitin C II [Anser anser]	found 1 time	1339
EL753486_2 AD0018024 Taenia solium UNAM-cd1_adult Taenia solium cDNA, mRNA sequence		
>gi|194944118|ref|XP_001983129.1| GG19855 [Drosophila erecta]	found 1 time	1426
EL752569_3 AD0014110 Taenia solium UNAM-cd1_adult Taenia solium cDNA, mRNA sequence		
>gi|313237647|emb|CBY12791.1| unnamed protein product [Oikopleura dioica]	found 1 time	527
EL743690_2 LV0260078 Taenia solium UNAM-cd2_larva Taenia solium cDNA, mRNA sequence		
>gi|149063232|gb|EDM13555.1| rCG21222, isoform CRA_a [Rattus norvegicus]	found 2 times	1236
EL763354_2 AD0202054 Taenia solium UNAM-cd1_adult Taenia solium cDNA, mRNA sequence		
EL741512_1 LV0227068 Taenia solium UNAM-cd2_larva Taenia solium cDNA, mRNA sequence		
>gi|332863200|ref|XP_003318055.1| PREDICTED: polyubiquitin-like [Pan troglodytes]	found 1 time	1073
EL744423_2 LV0272038 Taenia solium UNAM-cd2_larva Taenia solium cDNA, mRNA sequence		
>gi|325302604|tpg|DAA34466.1| TPA_exp: ubiquitin [Amblyomma variegatum]	found 4 times	891
GT227138_2 tscaa0_002708.z1.scf Taenia solium adult full-length cDNA library Taenia solium cDNA clone tscaa0_002708.z1.scf 5', mRNA sequence		
EL745475_2 LV0292009 Taenia solium UNAM-cd2_larva Taenia solium cDNA, mRNA sequence		
EL743675_3 LV0260063 Taenia solium UNAM-cd2_larva Taenia solium cDNA, mRNA sequence		
EL740690_2 LV0214015 Taenia solium UNAM-cd2_larva Taenia solium cDNA, mRNA sequence		
**Adenylate kinase**		
>gi|350579686|ref|XP_003122225.3| PREDICTED: adenylate kinase isoenzyme 1 isoform 1 [Sus scrofa]	found 1 time	225

Table 1: Continued.

**Protein representing protein group**<br>>Protein identified by ESTs<br>EST	number of ESTs linked to protein	TBLASTN score
sp|P00571|KAD1_PIG Adenylate kinase isoenzyme 1 OS=Sus scrofa GN=AK1 PE=1 SV=1		
>gi|319136965|ref|NP_001187771.1| adenylate kinase [Ictalurus punctatus]	found 3 times	284
GT227798_1 tscaa0_002440.z1.scf Taenia solium adult full-length cDNA library Taenia solium cDNA clone tscaa0_002440.z1.scf 5', mRNA sequence		
EL757531_6 AD0035124 Taenia solium UNAM-cd1_adult Taenia solium cDNA, mRNA sequence		
EL749389_4 AD0001012 Taenia solium UNAM-cd1_adult Taenia solium cDNA, mRNA sequence		
**Proteasome subunits**		
>gi|76156596|gb|AAX27775.2| SJCHGC06458 protein [Schistosoma japonicum]	found 1 time	237
EL746919_1 LV0315061 Taenia solium UNAM-cd2_larva Taenia solium cDNA, mRNA sequence		
>gi|226471366|emb|CAX70764.1| 20S proteasome subunit alpha 7 [Schistosoma japonicum]	found 1 time	237
EL743486_2 LV0258026 Taenia solium UNAM-cd2_larva Taenia solium cDNA, mRNA sequence		
>gi|256085175|ref|XP_002578798.1| proteasome subunit alpha 7 (T01 family) [Schistosoma mansoni]	found 3 times	589
EL756090_5 AD0028148 Taenia solium UNAM-cd1_adult Taenia solium cDNA, mRNA sequence		
EL751568_5 AD0010061 Taenia solium UNAM-cd1_adult Taenia solium cDNA, mRNA sequence		
EL751135_5 AD0008146 Taenia solium UNAM-cd1_adult Taenia solium cDNA, mRNA sequence		
>gi|167472829|gb|ABZ81034.1| proteasome alpha 2 subunit [Clonorchis sinensis]	found 1 time	456
EL752021_3 AD0012049 Taenia solium UNAM-cd1_adult Taenia solium cDNA, mRNA sequence		
>gi|89213678|gb|ABD64146.1| proteasome subunit alpha type 5 [Opisthorchis viverrini]	found 1 time	344
EL748296_1 LV0337017 Taenia solium UNAM-cd2_larva Taenia solium cDNA, mRNA sequence		
>gi|29841035|gb|AAP06048.1| similar to NM_017284 proteasome (prosome, macropain) subunit, beta type, 2 in Rattus norvegicus [Schistosoma japonicum]	found 2 times	645
EL761880_5 AD0173001 Taenia solium UNAM-cd1_adult Taenia solium cDNA, mRNA sequence		
EL757801_4 AD0096004 Taenia solium UNAM-cd1_adult Taenia solium cDNA, mRNA sequence		
>gi|56755093|gb|AAW25726.1| SJCHGC01130 protein [Schistosoma japonicum]	found 1 time	234
EL747722_1 LV0328010 Taenia solium UNAM-cd2_larva Taenia solium cDNA, mRNA sequence		
>gi|226478588|emb|CAX72789.1| proteasome (prosome, macropain) subunit, beta type 3 [Schistosoma japonicum]	found 7 times	237
EL760333_1 AD0140014 Taenia solium UNAM-cd1_adult Taenia solium cDNA, mRNA sequence		
EL759461_3 AD0123040 Taenia solium UNAM-cd1_adult Taenia solium cDNA, mRNA sequence		
EL756918_3 AD0031191 Taenia solium UNAM-cd1_adult Taenia solium cDNA, mRNA sequence		
EL750439_5 AD0005102 Taenia solium UNAM-cd1_adult Taenia solium cDNA, mRNA sequence		
EL750359_5 AD0005022 Taenia solium UNAM-cd1_adult Taenia solium cDNA, mRNA sequence		
EL750192_1 AD0004107 Taenia solium UNAM-cd1_adult Taenia solium cDNA, mRNA sequence		
EL750107_1 AD0004022 Taenia solium UNAM-cd1_adult Taenia solium cDNA, mRNA sequence		
>gi|167538768|ref|XP_001751043.1| hypothetical protein [Monosiga brevicollis MX1]	found 1 time	211
GT890564_1 TSAB.R74.esd Taenia solium cysticerca cDNA library Taenia solium cDNA, mRNA sequence		
>gi|61612096|gb|AAX47290.1| proteasome beta 1 subunit [Clonorchis sinensis]	found 1 time	254
EL753470_2 AD0018008 Taenia solium UNAM-cd1_adult Taenia solium cDNA, mRNA sequence		
**Heat shock protein 70**		
>gi|29336623|sp|Q24789.1|HSP70_ECHGR RecName: Full=Heat shock cognate 70 kDa protein; Short=HSP70	found 2 times	2882
GT227137_1 tscaa0_000639.z1.scf Taenia solium adult full-length cDNA library Taenia solium cDNA clone tscaa0_000639.z1.scf 5', mRNA sequence		
EL759703_2 AD0127044 Taenia solium UNAM-cd1_adult Taenia solium cDNA, mRNA sequence		
>gi|256033142|gb|ACU57188.1| HSP 70 [Echinococcus granulosus]	found 1 time	950
EL749310_1 LV0358021 Taenia solium UNAM-cd2_larva Taenia solium cDNA, mRNA sequence		
>gi|1661112|gb|AAB18390.1| heat shock 70kDa protein, partial [Mesocestoides corti]	found 5 times	2753
EL747588_1 LV0326004 Taenia solium UNAM-cd2_larva Taenia solium cDNA, mRNA sequence		
EL747068_3 LV0317065 Taenia solium UNAM-cd2_larva Taenia solium cDNA, mRNA sequence		
EL744968_2 LV0283033 Taenia solium UNAM-cd2_larva Taenia solium cDNA, mRNA sequence		
EL744008_3 LV0266008 Taenia solium UNAM-cd2_larva Taenia solium cDNA, mRNA sequence		
EL740495_2 LV0210019 Taenia solium UNAM-cd2_larva Taenia solium cDNA, mRNA sequence		
>gi|289186801|gb|ADC91992.1| heat shock protein 70 [Perna indica]	found 1 time	1001
EL745376_1 LV0290031 Taenia solium UNAM-cd2_larva Taenia solium cDNA, mRNA sequence		
>gi|178056512|ref|NP_001116600.1| heat shock 70 kDa protein 1-like [Sus scrofa]	found 1 time	2543
sp|A5A8V7|HS71L_PIG Heat shock 70 kDa protein 1-like OS=Sus scrofa GN=HSPA1L PE=2 SV=1		
>gi|62178034|gb|AAX73177.1| putative heat shock 70 kDa protein [Echinococcus granulosus]	found 1 time	1166
EL745660_2 LV0295018 Taenia solium UNAM-cd2_larva Taenia solium cDNA, mRNA sequence		
>gi|124783175|gb|ABN14909.1| heat shock protein 70 [Taenia asiatica]	found 2 times	936
GT227562_2 tscaa0_001631.z1.scf Taenia solium adult full-length cDNA library Taenia solium cDNA clone tscaa0_001631.z1.scf 5', mRNA sequence		
EL760000_1 AD0133045 Taenia solium UNAM-cd1_adult Taenia solium cDNA, mRNA sequence		
**Glyceraldehyde-3-phosphate dehydrogenase**		
>gi|329744642|ref|NP_001193288.1| glyceraldehyde-3-phosphate dehydrogenase [Sus scrofa]	found 1 time	1211
sp|P00355|G3P_PIG Glyceraldehyde-3-phosphate dehydrogenase OS=Sus scrofa GN=GAPDH PE=1 SV=4		
>gi|6016079|sp|Q27652.1|G3P_ECHMU RecName: Full=Glyceraldehyde-3-phosphate dehydrogenase; Short=GAPDH	found 1 time	1230
EL762910_5 AD0195030 Taenia solium UNAM-cd1_adult Taenia solium cDNA, mRNA sequence		
>gi|149364041|gb|ABR24229.1| gyceraldehyde-3-phosphate dehydrogenase [Taenia solium]	found 12 times	1323
GT892371_3 TSBS.R94.esd Taenia solium cysticerca cDNA library Taenia solium cDNA, mRNA sequence		
GT892221_2 TSCC.R59.esd Taenia solium cysticerca cDNA library Taenia solium cDNA, mRNA sequence		
GT891471_3 TSCB.R9.esd Taenia solium cysticerca cDNA library Taenia solium cDNA, mRNA sequence		
GT892641_3 TSBZ.R8.esd Taenia solium cysticerca cDNA library Taenia solium cDNA, mRNA sequence		
EX150392_3 TSEDTS1009G01 Cysti Taenia solium cDNA, mRNA sequence		
FD661653_3 TSEDTS1052G01 Cysti Taenia solium cDNA, mRNA sequence		
FD661459_3 TSEDTS1017G01 Cysti Taenia solium cDNA, mRNA sequence		
EL758807_3 AD0112006 Taenia solium UNAM-cd1_adult Taenia solium cDNA, mRNA sequence		
EL758545_3 AD0107038 Taenia solium UNAM-cd1_adult Taenia solium cDNA, mRNA sequence		
EL756270_3 AD0029065 Taenia solium UNAM-cd1_adult Taenia solium cDNA, mRNA sequence		
EL748320_3 LV0338009 Taenia solium UNAM-cd2_larva Taenia solium cDNA, mRNA sequence		
EL743807_2 LV0262048 Taenia solium UNAM-cd2_larva Taenia solium cDNA, mRNA sequence		

Table 1: Continued.

<table>
<tr><th>Protein representing protein group
>Protein identified by ESTs
EST</th><th>number of ESTs linked to protein</th><th>TBLASTN score</th></tr>
<tr><td>ES1 protein homolog</td><td></td><td></td></tr>
<tr><td>>gi|291290953|ref|NP_001167509.1| uncharacterized protein LOC100381155 [Xenopus laevis]</td><td>found 2 times</td><td>257</td></tr>
<tr><td>EL753679_3 AD0018217 Taenia solium UNAM-cd1_adult Taenia solium cDNA, mRNA sequence</td><td></td><td></td></tr>
<tr><td>EL744659_3 LV0277036 Taenia solium UNAM-cd2_larva Taenia solium cDNA, mRNA sequence</td><td></td><td></td></tr>
<tr><td>>gi|301613742|ref|XP_002936364.1| PREDICTED: ES1 protein homolog, mitochondrial [Xenopus (Silurana) tropicalis]</td><td>found 1 time</td><td>276</td></tr>
<tr><td>EL758380_1 AD0104065 Taenia solium UNAM-cd1_adult Taenia solium cDNA, mRNA sequence</td><td></td><td></td></tr>
<tr><td>>gi|390331662|ref|XP_003723328.1| PREDICTED: ES1 protein homolog, mitochondrial-like [Strongylocentrotus purpuratus]</td><td>found 2 times</td><td>267</td></tr>
<tr><td>EL744804_2 LV0280030 Taenia solium UNAM-cd2_larva Taenia solium cDNA, mRNA sequence</td><td></td><td></td></tr>
<tr><td>EL741907_1 LV0233040 Taenia solium UNAM-cd2_larva Taenia solium cDNA, mRNA sequence</td><td></td><td></td></tr>
<tr><td>Protein DJ-1-like</td><td></td><td></td></tr>
<tr><td>>gi|328782818|ref|XP_624271.2| PREDICTED: protein DJ-1-like [Apis mellifera]</td><td>found 3 times</td><td>273</td></tr>
<tr><td>EL760441_3 AD0142039 Taenia solium UNAM-cd1_adult Taenia solium cDNA, mRNA sequence</td><td></td><td></td></tr>
<tr><td>EL743223_2 LV0254004 Taenia solium UNAM-cd2_larva Taenia solium cDNA, mRNA sequence</td><td></td><td></td></tr>
<tr><td>EL743078_1 LV0251043 Taenia solium UNAM-cd2_larva Taenia solium cDNA, mRNA sequence</td><td></td><td></td></tr>
<tr><td>>gi|340724750|ref|XP_003400744.1| PREDICTED: LOW QUALITY PROTEIN: protein DJ-1-like [Bombus terrestris]</td><td>found 1 time</td><td>276</td></tr>
<tr><td>GT892892_1 TSBO.R32.esd Taenia solium cysticerca cDNA library Taenia solium cDNA, mRNA sequence</td><td></td><td></td></tr>
<tr><td>Phosphoglyceride transfer protein</td><td></td><td></td></tr>
<tr><td>>gi|124782980|gb|ABN14895.1| phosphoglyceride transfer protein, partial [Taenia asiatica]</td><td>found 7 times</td><td>290</td></tr>
<tr><td>EL758452_1 AD0105071 Taenia solium UNAM-cd1_adult Taenia solium cDNA, mRNA sequence</td><td></td><td></td></tr>
<tr><td>EL756935_1 AD0031208 Taenia solium UNAM-cd1_adult Taenia solium cDNA, mRNA sequence</td><td></td><td></td></tr>
<tr><td>EL755404_2 AD0025174 Taenia solium UNAM-cd1_adult Taenia solium cDNA, mRNA sequence</td><td></td><td></td></tr>
<tr><td>EL754519_2 AD0022044 Taenia solium UNAM-cd1_adult Taenia solium cDNA, mRNA sequence</td><td></td><td></td></tr>
<tr><td>EL752653_1 AD0014194 Taenia solium UNAM-cd1_adult Taenia solium cDNA, mRNA sequence</td><td></td><td></td></tr>
<tr><td>EL752495_2 AD0014036 Taenia solium UNAM-cd1_adult Taenia solium cDNA, mRNA sequence</td><td></td><td></td></tr>
<tr><td>EL751128_3 AD0008139 Taenia solium UNAM-cd1_adult Taenia solium cDNA, mRNA sequence</td><td></td><td></td></tr>
<tr><td>Tubulin</td><td></td><td></td></tr>
<tr><td>>gi|29337143|sp|Q9NFZ5.1|TBB3_ECHMU RecName: Full=Tubulin beta-3 chain; AltName: Full=Beta-3-tubulin</td><td>found 5 times</td><td>1741</td></tr>
<tr><td>GT890338_1 TSAP.R92.esd Taenia solium cysticerca cDNA library Taenia solium cDNA, mRNA sequence</td><td></td><td></td></tr>
<tr><td>EL757724_2 AD0094064 Taenia solium UNAM-cd1_adult Taenia solium cDNA, mRNA sequence</td><td></td><td></td></tr>
<tr><td>EL745395_2 LV0290050 Taenia solium UNAM-cd2_larva Taenia solium cDNA, mRNA sequence</td><td></td><td></td></tr>
<tr><td>EL741700_3 LV0230047 Taenia solium UNAM-cd2_larva Taenia solium cDNA, mRNA sequence</td><td></td><td></td></tr>
<tr><td>EL741594_1 LV0229005 Taenia solium UNAM-cd2_larva Taenia solium cDNA, mRNA sequence</td><td></td><td></td></tr>
<tr><td>>gi|135490|sp|P02554.1|TBB_PIG RecName: Full=Tubulin beta chain; AltName: Full=Beta-tubulin</td><td>found 1 time</td><td>1989</td></tr>
<tr><td>sp|P02554|TBB_PIG Tubulin beta chain OS=Sus scrofa PE=1 SV=1</td><td></td><td></td></tr>
<tr><td>>gi|148691289|gb|EDL23236.1| tubulin, beta 5 [Mus musculus]</td><td>found 1 time</td><td>1981</td></tr>
<tr><td>sp|Q767L7|TBB5_PIG Tubulin beta chain OS=Sus scrofa GN=TUBB PE=2 SV=1</td><td></td><td></td></tr>
<tr><td>>gi|156389348|ref|XP_001634953.1| predicted protein [Nematostella vectensis]</td><td>found 1 time</td><td>1954</td></tr>
<tr><td>EL750595_3 AD0006034 Taenia solium UNAM-cd1_adult Taenia solium cDNA, mRNA sequence</td><td></td><td></td></tr>
<tr><td>>gi|52626600|emb|CAG30543.1| beta tubulin [Glomus sp. BEG19]</td><td>found 1 time</td><td>869</td></tr>
<tr><td>EL748209_2 LV0335024 Taenia solium UNAM-cd2_larva Taenia solium cDNA, mRNA sequence</td><td></td><td></td></tr>
<tr><td>>gi|226475808|emb|CAX71994.1| Tubulin beta-2C chain [Schistosoma japonicum]</td><td>found 1 time</td><td>775</td></tr>
<tr><td>GT227072_1 tscaa0_001766.z1.scf Taenia solium adult full-length cDNA library Taenia solium cDNA clone tscaa0_001766.z1.scf 5', mRNA sequence</td><td></td><td></td></tr>
<tr><td>>gi|135425|sp|P09645.1|TBA8_CHICK RecName: Full=Tubulin alpha-8 chain</td><td>found 4 times</td><td>1465</td></tr>
<tr><td>EL761920_4 AD0173041 Taenia solium UNAM-cd1_adult Taenia solium cDNA, mRNA sequence</td><td></td><td></td></tr>
<tr><td>EL760898_4 AD0152023 Taenia solium UNAM-cd1_adult Taenia solium cDNA, mRNA sequence</td><td></td><td></td></tr>
<tr><td>EL759114_6 AD0117017 Taenia solium UNAM-cd1_adult Taenia solium cDNA, mRNA sequence</td><td></td><td></td></tr>
<tr><td>EL750486_4 AD0005149 Taenia solium UNAM-cd1_adult Taenia solium cDNA, mRNA sequence</td><td></td><td></td></tr>
<tr><td>>gi|1527170|gb|AAB07727.1| alpha-1 tubulin [Hirudo medicinalis]</td><td>found 2 times</td><td>1703</td></tr>
<tr><td>GT890399_3 TSAO.R56.esd Taenia solium cysticerca cDNA library Taenia solium cDNA, mRNA sequence</td><td></td><td></td></tr>
<tr><td>EL749770_1 AD0002134 Taenia solium UNAM-cd1_adult Taenia solium cDNA, mRNA sequence</td><td></td><td></td></tr>
<tr><td>>gi|405965637|gb|EKC30999.1| Tubulin alpha-1C chain [Crassostrea gigas]</td><td>found 3 times</td><td>1702</td></tr>
<tr><td>EL763478_2 AD0204041 Taenia solium UNAM-cd1_adult Taenia solium cDNA, mRNA sequence</td><td></td><td></td></tr>
<tr><td>EL763425_2 AD0203070 Taenia solium UNAM-cd1_adult Taenia solium cDNA, mRNA sequence</td><td></td><td></td></tr>
<tr><td>EL750993_2 AD0008004 Taenia solium UNAM-cd1_adult Taenia solium cDNA, mRNA sequence</td><td></td><td></td></tr>
<tr><td>>gi|148667971|gb|EDL00388.1| tubulin, alpha 4, isoform CRA_b [Mus musculus]</td><td>found 1 time</td><td>1192</td></tr>
<tr><td>EL761378_4 AD0162039 Taenia solium UNAM-cd1_adult Taenia solium cDNA, mRNA sequence</td><td></td><td></td></tr>
<tr><td>>gi|154091298|gb|ABS57456.1| alpha-tubulin [Heliconius melpomene]</td><td>found 2 times</td><td>1038</td></tr>
<tr><td>EL759137_2 AD0117040 Taenia solium UNAM-cd1_adult Taenia solium cDNA, mRNA sequence</td><td></td><td></td></tr>
<tr><td>EL758875_2 AD0113016 Taenia solium UNAM-cd1_adult Taenia solium cDNA, mRNA sequence</td><td></td><td></td></tr>
<tr><td>>gi|295901419|dbj|BAJ07366.1| alpha-tubulin [Taenia taeniaeformis]</td><td>found 3 times</td><td>795</td></tr>
<tr><td>GT227667_3 tscaa0_001917.z1.scf Taenia solium adult full-length cDNA library Taenia solium cDNA clone tscaa0_001917.z1.scf 5', mRNA sequence</td><td></td><td></td></tr>
<tr><td>EL760348_3 AD0140029 Taenia solium UNAM-cd1_adult Taenia solium cDNA, mRNA sequence</td><td></td><td></td></tr>
<tr><td>EL758491_2 AD0106034 Taenia solium UNAM-cd1_adult Taenia solium cDNA, mRNA sequence</td><td></td><td></td></tr>
<tr><td>>gi|124783791|gb|ABN14943.1| neural class-II beta tubulin [Taenia asiatica]</td><td>found 2 times</td><td>505</td></tr>
<tr><td>EL748713_2 LV0344036 Taenia solium UNAM-cd2_larva Taenia solium cDNA, mRNA sequence</td><td></td><td></td></tr>
<tr><td>EL748613_1 LV0342064 Taenia solium UNAM-cd2_larva Taenia solium cDNA, mRNA sequence</td><td></td><td></td></tr>
<tr><td>Thioredoxin</td><td></td><td></td></tr>
<tr><td>>gi|29337032|sp|O17486.2|THIO_ECHGR RecName: Full=Thioredoxin; Short=Trx</td><td>found 18 times</td><td>281</td></tr>
<tr><td>EL757027_3 AD0032039 Taenia solium UNAM-cd1_adult Taenia solium cDNA, mRNA sequence</td><td></td><td></td></tr>
<tr><td>EL755857_3 AD0027153 Taenia solium UNAM-cd1_adult Taenia solium cDNA, mRNA sequence</td><td></td><td></td></tr>
<tr><td>EL755792_2 AD0027088 Taenia solium UNAM-cd1_adult Taenia solium cDNA, mRNA sequence</td><td></td><td></td></tr>
<tr><td>EL753853_1 AD0019140 Taenia solium UNAM-cd1_adult Taenia solium cDNA, mRNA sequence</td><td></td><td></td></tr>
<tr><td>EL753413_4 AD0017171 Taenia solium UNAM-cd1_adult Taenia solium cDNA, mRNA sequence</td><td></td><td></td></tr>
<tr><td>EL753183_1 AD0016221 Taenia solium UNAM-cd1_adult Taenia solium cDNA, mRNA sequence</td><td></td><td></td></tr>
<tr><td>EL752302_4 AD0013069 Taenia solium UNAM-cd1_adult Taenia solium cDNA, mRNA sequence</td><td></td><td></td></tr>
<tr><td>EL752052_3 AD0012080 Taenia solium UNAM-cd1_adult Taenia solium cDNA, mRNA sequence</td><td></td><td></td></tr>
</table>

Table 1: Continued.

<table>
<tr><th>Protein representing protein group
>Protein identified by ESTs
EST</th><th>number of ESTs linked to protein</th><th>TBLASTN score</th></tr>
<tr><td>EL746355_3 LV0308005 Taenia solium UNAM-cd2_larva Taenia solium cDNA, mRNA sequence</td><td></td><td></td></tr>
<tr><td>EL746336_3 LV0307050 Taenia solium UNAM-cd2_larva Taenia solium cDNA, mRNA sequence</td><td></td><td></td></tr>
<tr><td>EL745993_2 LV0300024 Taenia solium UNAM-cd2_larva Taenia solium cDNA, mRNA sequence</td><td></td><td></td></tr>
<tr><td>EL745052_2 LV0285024 Taenia solium UNAM-cd2_larva Taenia solium cDNA, mRNA sequence</td><td></td><td></td></tr>
<tr><td>EL744830_1 LV0280056 Taenia solium UNAM-cd2_larva Taenia solium cDNA, mRNA sequence</td><td></td><td></td></tr>
<tr><td>EL744735_2 LV0279004 Taenia solium UNAM-cd2_larva Taenia solium cDNA, mRNA sequence</td><td></td><td></td></tr>
<tr><td>EL742821_1 LV0247039 Taenia solium UNAM-cd2_larva Taenia solium cDNA, mRNA sequence</td><td></td><td></td></tr>
<tr><td>EL742618_1 LV0244049 Taenia solium UNAM-cd2_larva Taenia solium cDNA, mRNA sequence</td><td></td><td></td></tr>
<tr><td>EL741429_2 LV0226048 Taenia solium UNAM-cd2_larva Taenia solium cDNA, mRNA sequence</td><td></td><td></td></tr>
<tr><td>EL741133_3 LV0221034 Taenia solium UNAM-cd2_larva Taenia solium cDNA, mRNA sequence</td><td></td><td></td></tr>
<tr><td>>gi|340372669|ref|XP_003384866.1| PREDICTED: protein disulfide-isomerase A3-like [Amphimedon queenslandica]</td><td>found 1 time</td><td>536</td></tr>
<tr><td>EL747314_1 LV0321045 Taenia solium UNAM-cd2_larva Taenia solium cDNA, mRNA sequence</td><td></td><td></td></tr>
<tr><td>>gi|393905984|gb|EJD74130.1| disulfide-isomerase A4 [Loa loa]</td><td>found 1 time</td><td>529</td></tr>
<tr><td>EL758247_3 AD0102058 Taenia solium UNAM-cd1_adult Taenia solium cDNA, mRNA sequence</td><td></td><td></td></tr>
<tr><td>>gi|402587826|gb|EJW81760.1| protein disulfide isomerase associated 4 [Wuchereria bancrofti]</td><td>found 2 times</td><td>414</td></tr>
<tr><td>EL749154_3 LV0354013 Taenia solium UNAM-cd2_larva Taenia solium cDNA, mRNA sequence</td><td></td><td></td></tr>
<tr><td>EL741962_3 LV0234023 Taenia solium UNAM-cd2_larva Taenia solium cDNA, mRNA sequence</td><td></td><td></td></tr>
<tr><td>>gi|393910969|gb|EFO28237.2| disulfide isomerase [Loa loa]</td><td>found 1 time</td><td>599</td></tr>
<tr><td>EL747020_3 LV0317017 Taenia solium UNAM-cd2_larva Taenia solium cDNA, mRNA sequence</td><td></td><td></td></tr>
<tr><td>SJCHGC06082 protein</td><td></td><td></td></tr>
<tr><td>>gi|29841130|gb|AAP06143.1| SJCHGC06082 protein [Schistosoma japonicum]</td><td>found 7 times</td><td>263</td></tr>
<tr><td>GT891853_1 TSAC.R22.esd Taenia solium cysticerca cDNA library Taenia solium cDNA, mRNA sequence</td><td></td><td></td></tr>
<tr><td>GT891531_2 TSAV.R91.esd Taenia solium cysticerca cDNA library Taenia solium cDNA, mRNA sequence</td><td></td><td></td></tr>
<tr><td>GT891148_1 TSAC.R34.esd Taenia solium cysticerca cDNA library Taenia solium cDNA, mRNA sequence</td><td></td><td></td></tr>
<tr><td>EL760751_1 AD0148029 Taenia solium UNAM-cd1_adult Taenia solium cDNA, mRNA sequence</td><td></td><td></td></tr>
<tr><td>EL756435_2 AD0029230 Taenia solium UNAM-cd1_adult Taenia solium cDNA, mRNA sequence</td><td></td><td></td></tr>
<tr><td>EL754674_2 AD0022199 Taenia solium UNAM-cd1_adult Taenia solium cDNA, mRNA sequence</td><td></td><td></td></tr>
<tr><td>EL749594_3 AD0001217 Taenia solium UNAM-cd1_adult Taenia solium cDNA, mRNA sequence</td><td></td><td></td></tr>
<tr><td>>gi|358339016|dbj|GAA47156.1| cytochrome c [Clonorchis sinensis]</td><td>found 1 time</td><td>269</td></tr>
<tr><td>GT891706_2 TSBJ.R68.esd Taenia solium cysticerca cDNA library Taenia solium cDNA, mRNA sequence</td><td></td><td></td></tr>
<tr><td>Small GTPase RhoA</td><td></td><td></td></tr>
<tr><td>>gi|74096011|ref|NP_001027690.1| RhoA protein [Ciona intestinalis]</td><td>found 1 time</td><td>496</td></tr>
<tr><td>EL754335_2 AD0021125 Taenia solium UNAM-cd1_adult Taenia solium cDNA, mRNA sequence</td><td></td><td></td></tr>
<tr><td>>gi|291243430|ref|XP_002741600.1| PREDICTED: ras homolog gene family, member A-like isoform 1 [Saccoglossus kowalevskii]</td><td>found 3 times</td><td>497</td></tr>
<tr><td>EL752414_4 AD0013181 Taenia solium UNAM-cd1_adult Taenia solium cDNA, mRNA sequence</td><td></td><td></td></tr>
<tr><td>EL743795_1 LV0262036 Taenia solium UNAM-cd2_larva Taenia solium cDNA, mRNA sequence</td><td></td><td></td></tr>
<tr><td>EL742396_2 LV0241022 Taenia solium UNAM-cd2_larva Taenia solium cDNA, mRNA sequence</td><td></td><td></td></tr>
<tr><td>>gi|124784148|gb|ABN14965.1| small GTPase RhoA [Taenia asiatica]</td><td>found 5 times</td><td>549</td></tr>
<tr><td>GT891188_1 TSAQ.R75.esd Taenia solium cysticerca cDNA library Taenia solium cDNA, mRNA sequence</td><td></td><td></td></tr>
<tr><td>EL758705_1 AD0110014 Taenia solium UNAM-cd1_adult Taenia solium cDNA, mRNA sequence</td><td></td><td></td></tr>
<tr><td>EL747606_2 LV0326022 Taenia solium UNAM-cd2_larva Taenia solium cDNA, mRNA sequence</td><td></td><td></td></tr>
<tr><td>EL740802_3 LV0216036 Taenia solium UNAM-cd2_larva Taenia solium cDNA, mRNA sequence</td><td></td><td></td></tr>
<tr><td>EL740590_2 LV0212026 Taenia solium UNAM-cd2_larva Taenia solium cDNA, mRNA sequence</td><td></td><td></td></tr>
<tr><td>>gi|194762716|ref|XP_001963480.1| GF20423 [Drosophila ananassae]</td><td>found 1 time</td><td>428</td></tr>
<tr><td>GT889968_2 TSBC.R71.esd Taenia solium cysticerca cDNA library Taenia solium cDNA, mRNA sequence</td><td></td><td></td></tr>
<tr><td>>gi|333944141|pdb|2YIN|C Chain C, Structure Of The Complex Between Dock2 And Rac1.</td><td>found 1 time</td><td>398</td></tr>
<tr><td>GT890168_1 TSAG.R62.esd Taenia solium cysticerca cDNA library Taenia solium cDNA, mRNA sequence</td><td></td><td></td></tr>
<tr><td>>gi|56753633|gb|AAW25019.1| SJCHGC09359 protein [Schistosoma japonicum]</td><td>found 1 time</td><td>355</td></tr>
<tr><td>EL745793_1 LV0297022 Taenia solium UNAM-cd2_larva Taenia solium cDNA, mRNA sequence</td><td></td><td></td></tr>
<tr><td>>gi|178056558|ref|NP_001116652.1| ras-related protein Rab-5A [Sus scrofa]</td><td>found 1 time</td><td>311</td></tr>
<tr><td>sp|Q06AU6|RAB5A_PIG Ras-related protein Rab-5A OS=Sus scrofa GN=RAB5A PE=2 SV=1</td><td></td><td></td></tr>
<tr><td>>gi|357119478|ref|XP_003561466.1| PREDICTED: ras-related protein RABF2b-like [Brachypodium distachyon]</td><td>found 3 times</td><td>306</td></tr>
<tr><td>EL761207_6 AD0159004 Taenia solium UNAM-cd1_adult Taenia solium cDNA, mRNA sequence</td><td></td><td></td></tr>
<tr><td>EL752325_6 AD0013092 Taenia solium UNAM-cd1_adult Taenia solium cDNA, mRNA sequence</td><td></td><td></td></tr>
<tr><td>EL752079_2 AD0012107 Taenia solium UNAM-cd1_adult Taenia solium cDNA, mRNA sequence</td><td></td><td></td></tr>
<tr><td>>gi|341883094|gb|EGT39029.1| hypothetical protein CAEBREN_12928 [Caenorhabditis brenneri]</td><td>found 1 time</td><td>369</td></tr>
<tr><td>EL750680_3 AD0006119 Taenia solium UNAM-cd1_adult Taenia solium cDNA, mRNA sequence</td><td></td><td></td></tr>
<tr><td>>gi|297262255|ref|XP_001104802.2| PREDICTED: ADP-ribosylation factor 3-like isoform 3 [Macaca mulatta]</td><td>found 1 time</td><td>577</td></tr>
<tr><td>GT890039_3 TSBB.R27.esd Taenia solium cysticerca cDNA library Taenia solium cDNA, mRNA sequence</td><td></td><td></td></tr>
<tr><td>>gi|385304347|gb|EIF48368.1| adp-ribosylation factor [Dekkera bruxellensis AWRI1499]</td><td>found 1 time</td><td>569</td></tr>
<tr><td>GT889521_3 TSBE.R81.esd Taenia solium cysticerca cDNA library Taenia solium cDNA, mRNA sequence</td><td></td><td></td></tr>
<tr><td>Calpain</td><td></td><td></td></tr>
<tr><td>>gi|146741270|dbj|BAF62290.1| calpain [Schistosoma haematobium]</td><td>found 1 time</td><td>328</td></tr>
<tr><td>EL747664_3 LV0327007 Taenia solium UNAM-cd2_larva Taenia solium cDNA, mRNA sequence</td><td></td><td></td></tr>
<tr><td>>gi|56757862|gb|AAW27071.1| unknown [Schistosoma japonicum]</td><td>found 1 time</td><td>251</td></tr>
<tr><td>EL748263_1 LV0336039 Taenia solium UNAM-cd2_larva Taenia solium cDNA, mRNA sequence</td><td></td><td></td></tr>
<tr><td>>gi|56756901|gb|AAW26622.1| SJCHGC01809 protein [Schistosoma japonicum]</td><td>found 1 time</td><td>314</td></tr>
<tr><td>EL759695_1 AD0127036 Taenia solium UNAM-cd1_adult Taenia solium cDNA, mRNA sequence</td><td></td><td></td></tr>
<tr><td>>gi|256087627|ref|XP_002579967.1| family C2 unassigned peptidase (C02 family) [Schistosoma mansoni]</td><td>found 1 time</td><td>311</td></tr>
<tr><td>GT891552_1 TSAZ.R59.esd Taenia solium cysticerca cDNA library Taenia solium cDNA, mRNA sequence</td><td></td><td></td></tr>
<tr><td>>gi|256087629|ref|XP_002579968.1| family C2 unassigned peptidase (C02 family) [Schistosoma mansoni]</td><td>found 1 time</td><td>311</td></tr>
<tr><td>EL740626_2 LV0213008 Taenia solium UNAM-cd2_larva Taenia solium cDNA, mRNA sequence</td><td></td><td></td></tr>
<tr><td>Calumenin</td><td></td><td></td></tr>
<tr><td>>gi|124784033|gb|ABN14961.1| calumenin [Taenia asiatica]</td><td>found 2 times</td><td>348</td></tr>
<tr><td>GT889809_4 TSCB.R33.esd Taenia solium cysticerca cDNA library Taenia solium cDNA, mRNA sequence</td><td></td><td></td></tr>
<tr><td>EL742874_1 LV0248036 Taenia solium UNAM-cd2_larva Taenia solium cDNA, mRNA sequence</td><td></td><td></td></tr>
<tr><td>Calcium-binding protein</td><td></td><td></td></tr>
</table>

Table 1: Continued.

**Protein representing protein group**<br>>Protein identified by ESTs<br>EST	number of ESTs linked to protein	TBLASTN score
>gi|256071353|ref|XP_002572005.1| calcium-binding protein [Schistosoma mansoni]	found 2 times	355
GT227350_1 tscaa0_000959.z1.scf Taenia solium adult full-length cDNA library Taenia solium cDNA clone tscaa0_000959.z1.scf 5', mRNA sequence		
EL762844_2 AD0194015 Taenia solium UNAM-cd1_adult Taenia solium cDNA, mRNA sequence		
**Adenosylhomocysteinase**		
>gi|58801555|ref|NP_001011727.1| adenosylhomocysteinase [Sus scrofa]	found 1 time	1544
sp|Q710C4|SAHH_PIG Adenosylhomocysteinase OS=Sus scrofa GN=AHCY PE=3 SV=3		
>gi|40363541|ref|NP_954688.1| adenosylhomocysteinase [Danio rerio]	found 2 times	1705
EL749811_3 AD0002175 Taenia solium UNAM-cd1_adult Taenia solium cDNA, mRNA sequence		
EL742665_2 LV0245020 Taenia solium UNAM-cd2_larva Taenia solium cDNA, mRNA sequence		
>gi|256075976|ref|XP_002574291.1| adenosylhomocysteinase [Schistosoma mansoni]	found 1 time	1201
EL752451_4 AD0013218 Taenia solium UNAM-cd1_adult Taenia solium cDNA, mRNA sequence		
**Aldo-keto reductase**		
>gi|148231135|ref|NP_001079568.1| aldo-keto reductase family 1, member C2 (dihydrodiol dehydrogenase 2; bile acid binding protein; 3-alpha hydroxysteroid dehydrogenase, type III) [Xenopus laevis]	found 1 time	269
EL747815_2 LV0329053 Taenia solium UNAM-cd2_larva Taenia solium cDNA, mRNA sequence		
>gi|195435013|ref|XP_002065496.1| GK14636 [Drosophila willistoni]	found 1 time	317
EL742064_1 LV0235053 Taenia solium UNAM-cd2_larva Taenia solium cDNA, mRNA sequence		
>gi|256080714|ref|XP_002576623.1| pol-related [Schistosoma mansoni]	found 22 times	273
EL763228_1 AD0200043 Taenia solium UNAM-cd1_adult Taenia solium cDNA, mRNA sequence		
EL762869_3 AD0194040 Taenia solium UNAM-cd1_adult Taenia solium cDNA, mRNA sequence		
EL762690_3 AD0191051 Taenia solium UNAM-cd1_adult Taenia solium cDNA, mRNA sequence		
EL762033_5 AD0177004 Taenia solium UNAM-cd1_adult Taenia solium cDNA, mRNA sequence		
EL761760_6 AD0170031 Taenia solium UNAM-cd1_adult Taenia solium cDNA, mRNA sequence		
EL761245_6 AD0159042 Taenia solium UNAM-cd1_adult Taenia solium cDNA, mRNA sequence		
EL760167_2 AD0136058 Taenia solium UNAM-cd1_adult Taenia solium cDNA, mRNA sequence		
EL757453_3 AD0035046 Taenia solium UNAM-cd1_adult Taenia solium cDNA, mRNA sequence		
EL757325_3 AD0034141 Taenia solium UNAM-cd1_adult Taenia solium cDNA, mRNA sequence		
EL756981_1 AD0031254 Taenia solium UNAM-cd1_adult Taenia solium cDNA, mRNA sequence		
EL756164_1 AD0028222 Taenia solium UNAM-cd1_adult Taenia solium cDNA, mRNA sequence		
EL755932_2 AD0027228 Taenia solium UNAM-cd1_adult Taenia solium cDNA, mRNA sequence		
EL755860_3 AD0027156 Taenia solium UNAM-cd1_adult Taenia solium cDNA, mRNA sequence		
EL755366_2 AD0025136 Taenia solium UNAM-cd1_adult Taenia solium cDNA, mRNA sequence		
EL753608_2 AD0018146 Taenia solium UNAM-cd1_adult Taenia solium cDNA, mRNA sequence		
EL749436_2 AD0001059 Taenia solium UNAM-cd1_adult Taenia solium cDNA, mRNA sequence		
EL747976_1 LV0331069 Taenia solium UNAM-cd2_larva Taenia solium cDNA, mRNA sequence		
EL746815_3 LV0314028 Taenia solium UNAM-cd2_larva Taenia solium cDNA, mRNA sequence		
EL744538_1 LV0275013 Taenia solium UNAM-cd2_larva Taenia solium cDNA, mRNA sequence		
EL743106_2 LV0252007 Taenia solium UNAM-cd2_larva Taenia solium cDNA, mRNA sequence		
EL741599_1 LV0229010 Taenia solium UNAM-cd2_larva Taenia solium cDNA, mRNA sequence		
EL741054_1 LV0220027 Taenia solium UNAM-cd2_larva Taenia solium cDNA, mRNA sequence		
>gi|226472500|emb|CAX77286.1| aldo-keto reductase family 1, member B4 (aldose reductase) [Schistosoma japonicum]	found 1 time	218
EL743423_3 LV0257040 Taenia solium UNAM-cd2_larva Taenia solium cDNA, mRNA sequence		
**Glutathione S-transferase**		
>gi|225355240|gb|ACN88552.1| sigma-type glutathione S-transferase [Taenia solium]	found 2 times	440
EL761333_3 AD0161050 Taenia solium UNAM-cd1_adult Taenia solium cDNA, mRNA sequence		
EL752891_2 AD0015150 Taenia solium UNAM-cd1_adult Taenia solium cDNA, mRNA sequence		
>gi|60418504|gb|AAX19694.1| 26.5 kDa glutathione S-transferase [Taenia solium]	found 2 times	97
EL745513_1 LV0292047 Taenia solium UNAM-cd2_larva Taenia solium cDNA, mRNA sequence		
EL744606_2 LV0276024 Taenia solium UNAM-cd2_larva Taenia solium cDNA, mRNA sequence		
>gi|21591409|gb|AAM64045.1|AF403222_1 glutathione S-transferase [Taenia solium]	found 22 times	181
EL762417_6 AD0185022 Taenia solium UNAM-cd1_adult Taenia solium cDNA, mRNA sequence		
EL762411_4 AD0185016 Taenia solium UNAM-cd1_adult Taenia solium cDNA, mRNA sequence		
EL762064_6 AD0177035 Taenia solium UNAM-cd1_adult Taenia solium cDNA, mRNA sequence		
EL762042_4 AD0177013 Taenia solium UNAM-cd1_adult Taenia solium cDNA, mRNA sequence		
EL761791_4 AD0171008 Taenia solium UNAM-cd1_adult Taenia solium cDNA, mRNA sequence		
EL761741_6 AD0170012 Taenia solium UNAM-cd1_adult Taenia solium cDNA, mRNA sequence		
EL761647_6 AD0168007 Taenia solium UNAM-cd1_adult Taenia solium cDNA, mRNA sequence		
EL761516_5 AD0165014 Taenia solium UNAM-cd1_adult Taenia solium cDNA, mRNA sequence		
EL759115_1 AD0117018 Taenia solium UNAM-cd1_adult Taenia solium cDNA, mRNA sequence		
EL757625_2 AD0035218 Taenia solium UNAM-cd1_adult Taenia solium cDNA, mRNA sequence		
EL757561_1 AD0035154 Taenia solium UNAM-cd1_adult Taenia solium cDNA, mRNA sequence		
EL757409_6 AD0035002 Taenia solium UNAM-cd1_adult Taenia solium cDNA, mRNA sequence		
EL755802_2 AD0027098 Taenia solium UNAM-cd1_adult Taenia solium cDNA, mRNA sequence		
EL753737_2 AD0019024 Taenia solium UNAM-cd1_adult Taenia solium cDNA, mRNA sequence		
EL751869_5 AD0011112 Taenia solium UNAM-cd1_adult Taenia solium cDNA, mRNA sequence		
EL750428_5 AD0005091 Taenia solium UNAM-cd1_adult Taenia solium cDNA, mRNA sequence		
EL750426_4 AD0005089 Taenia solium UNAM-cd1_adult Taenia solium cDNA, mRNA sequence		
EL750179_2 AD0004094 Taenia solium UNAM-cd1_adult Taenia solium cDNA, mRNA sequence		
EL749913_4 AD0003055 Taenia solium UNAM-cd1_adult Taenia solium cDNA, mRNA sequence		
EL749692_3 AD0002056 Taenia solium UNAM-cd1_adult Taenia solium cDNA, mRNA sequence		
EL745843_3 LV0297072 Taenia solium UNAM-cd2_larva Taenia solium cDNA, mRNA sequence		
EL742539_3 LV0243037 Taenia solium UNAM-cd2_larva Taenia solium cDNA, mRNA sequence		
**Glycolipid transfer protein-like protein**		
>gi|124782916|gb|ABN14889.1| glycolipid transfer protein-like protein [Taenia asiatica]	found 2 times	361
EL759685_3 AD0127026 Taenia solium UNAM-cd1_adult Taenia solium cDNA, mRNA sequence		
EL759597_2 AD0125055 Taenia solium UNAM-cd1_adult Taenia solium cDNA, mRNA sequence		

Table 1: Continued.

**Protein representing protein group**<br>>Protein identified by ESTs<br>EST	number of ESTs linked to protein	TBLASTN score
**UDP-glucose 4-epimerase**		
>gi|170044588|ref|XP_001849924.1| UDP-glucose 4-epimerase [Culex quinquefasciatus]	found 1 time	271
EL762658_1 AD0191019 Taenia solium UNAM-cd1_adult Taenia solium cDNA, mRNA sequence		
>gi|260799292|ref|XP_002594631.1| hypothetical protein BRAFLDRAFT_217512 [Branchiostoma floridae]	found 1 time	284
EL749373_2 LV0359051 Taenia solium UNAM-cd2_larva Taenia solium cDNA, mRNA sequence		
**Alpha-2-macroglobulin-like protein 1**		
>gi|358333571|dbj|GAA52061.1| alpha-2-macroglobulin-like protein 1 [Clonorchis sinensis]	found 5 times	654
GT891124_1 TSCE.R81.esd Taenia solium cysticerca cDNA library Taenia solium cDNA, mRNA sequence		
EL759400_2 AD0122044 Taenia solium UNAM-cd1_adult Taenia solium cDNA, mRNA sequence		
EL758275_1 AD0103019 Taenia solium UNAM-cd1_adult Taenia solium cDNA, mRNA sequence		
EL758164_3 AD0101044 Taenia solium UNAM-cd1_adult Taenia solium cDNA, mRNA sequence		
EL745463_3 LV0291055 Taenia solium UNAM-cd2_larva Taenia solium cDNA, mRNA sequence		
**Carbonyl reductase**		
>gi|256077000|ref|XP_002574796.1| carbonyl reductase [Schistosoma mansoni]	found 1 time	295
GT227102_2 tscaa0_000437.z1.scf Taenia solium adult full-length cDNA library Taenia solium cDNA clone tscaa0_000437.z1.scf 5', mRNA sequence		
>gi|226472434|emb|CAX77253.1| carbonyl reductase 1 [Schistosoma japonicum]	found 1 time	377
EL745798_3 LV0297027 Taenia solium UNAM-cd2_larva Taenia solium cDNA, mRNA sequence		
>gi|226472450|emb|CAX77261.1| carbonyl reductase 1 [Schistosoma japonicum]	found 1 time	377
GT892548_2 TSAA.R85.esd Taenia solium cysticerca cDNA library Taenia solium cDNA, mRNA sequence		
**Gamma-soluble NSF attachment protein**		
>gi|29841166|gb|AAP06179.1| similar to GenBank Accession Number AE003462 gamma Snap gene product in Drosophila melanogaster [Schistosoma japonicum]	found 4 times	265
EL762147_6 AD0179040 Taenia solium UNAM-cd1_adult Taenia solium cDNA, mRNA sequence		
EL757205_1 AD0034021 Taenia solium UNAM-cd1_adult Taenia solium cDNA, mRNA sequence		
EL754991_3 AD0024008 Taenia solium UNAM-cd1_adult Taenia solium cDNA, mRNA sequence		
EL742171_3 LV0237059 Taenia solium UNAM-cd2_larva Taenia solium cDNA, mRNA sequence		
>gi|189502834|gb|ACE06798.1| unknown [Schistosoma japonicum]	found 2 times	265
EL763339_1 AD0202039 Taenia solium UNAM-cd1_adult Taenia solium cDNA, mRNA sequence		
EL755899_2 AD0027195 Taenia solium UNAM-cd1_adult Taenia solium cDNA, mRNA sequence		
>gi|256080614|ref|XP_002576574.1| gamma-soluble nsf attachment protein (snap) [Schistosoma mansoni]	found 1 time	263
EL761742_6 AD0170013 Taenia solium UNAM-cd1_adult Taenia solium cDNA, mRNA sequence		
>gi|358339484|dbj|GAA47540.1| gamma-soluble NSF attachment protein, partial [Clonorchis sinensis]	found 3 times	278
EL755901_3 AD0027197 Taenia solium UNAM-cd1_adult Taenia solium cDNA, mRNA sequence		
EL752355_5 AD0013122 Taenia solium UNAM-cd1_adult Taenia solium cDNA, mRNA sequence		
EL752112_1 AD0012140 Taenia solium UNAM-cd1_adult Taenia solium cDNA, mRNA sequence		
**UDP-glucose pyrophosphorylase 2**		
>gi|56758962|gb|AAW27621.1| SJCHGC01041 protein [Schistosoma japonicum]	found 1 time	248
GT226931_2 tscaa0_000936.z1.scf Taenia solium adult full-length cDNA library Taenia solium cDNA clone tscaa0_000936.z1.scf 5', mRNA sequence		
>gi|256074471|ref|XP_002573548.1| utp-glucose-1-phosphate uridylyltransferase 2 (udp-glucose pyrophosphorylase 2) [Schistosoma mansoni]	found 1 time	280
EL748674_3 LV0343059 Taenia solium UNAM-cd2_larva Taenia solium cDNA, mRNA sequence		
Hypothetical protein SINV_09109		
>gi|322793762|gb|EFZ17146.1| hypothetical protein SINV_09109 [Solenopsis invicta]	found 2 times	289
GT890718_2 TSAP.R80.esd Taenia solium cysticerca cDNA library Taenia solium cDNA, mRNA sequence		
EL742820_2 LV0247038 Taenia solium UNAM-cd2_larva Taenia solium cDNA, mRNA sequence		
**Phosphoglycerate kinase**		
>gi|47523276|ref|NP_998947.1| phosphoglycerate kinase 2 [Sus scrofa]	found 1 time	543
sp|Q6RI85|PGK2_PIG Phosphoglycerate kinase 2 OS=Sus scrofa GN=PGK2 PE=2 SV=3		
>gi|153792027|ref|NP_001093402.1| phosphoglycerate kinase 1 [Sus scrofa]	found 1 time	578
sp|Q7SIB7|PGK1_PIG Phosphoglycerate kinase 1 OS=Sus scrofa GN=PGK1 PE=1 SV=3		
>gi|62858027|ref|NP_001016545.1| phosphoglycerate kinase 1 [Xenopus (Silurana) tropicalis]	found 1 time	519
EL740229_2 LV0205009 Taenia solium UNAM-cd2_larva Taenia solium cDNA, mRNA sequence		
>gi|346470313|gb|AEO35001.1| hypothetical protein [Amblyomma maculatum]	found 1 time	498
EL757927_1 AD0098012 Taenia solium UNAM-cd1_adult Taenia solium cDNA, mRNA sequence		
>gi|358333945|dbj|GAA52400.1| phosphoglycerate kinase [Clonorchis sinensis]	found 1 time	562
EL741199_1 LV0222032 Taenia solium UNAM-cd2_larva Taenia solium cDNA, mRNA sequence		
**Enolase**		
>gi|113205498|ref|NP_001037992.1| beta-enolase [Sus scrofa]	found 1 time	1504
sp|Q1KYT0|ENOB_PIG Beta-enolase OS=Sus scrofa GN=ENO3 PE=2 SV=1		
>gi|261266611|gb|ACX56268.1| enolase [Taenia asiatica]	found 1 time	1674
EL744539_3 LV0275014 Taenia solium UNAM-cd2_larva Taenia solium cDNA, mRNA sequence		
>gi|386873714|gb|AFJ44747.1| enolase [Taenia multiceps]	found 3 times	1683
EL762810_2 AD0193057 Taenia solium UNAM-cd1_adult Taenia solium cDNA, mRNA sequence		
EL744862_3 LV0281022 Taenia solium UNAM-cd2_larva Taenia solium cDNA, mRNA sequence		
EL741406_3 LV0226025 Taenia solium UNAM-cd2_larva Taenia solium cDNA, mRNA sequence		
**Elongation factors**		
>gi|257215780|emb|CAX83042.1| eukaryotic translation elongation factor 1 gamma [Schistosoma japonicum]	found 1 time	235
EL763151_3 AD0199035 Taenia solium UNAM-cd1_adult Taenia solium cDNA, mRNA sequence		
>gi|358336196|dbj|GAA38919.2| elongation factor 1-gamma, partial [Clonorchis sinensis]	found 1 time	246
EL744849_3 LV0281009 Taenia solium UNAM-cd2_larva Taenia solium cDNA, mRNA sequence		
>gi|226480744|emb|CAX73469.1| Elongation FacTor [Schistosoma japonicum]	found 1 time	1394
EL741012_3 LV0219062 Taenia solium UNAM-cd2_larva Taenia solium cDNA, mRNA sequence		

Table 1: Continued.

**Protein representing protein group**<br>>Protein identified by ESTs<br>EST	number of ESTs linked to protein	TBLASTN score
>gi|148717331|dbj|BAF63679.1| elongation factor 1 alpha [Echinococcus vogeli]	found 2 times	1205
EL757550_1 AD0035143 Taenia solium UNAM-cd1_adult Taenia solium cDNA, mRNA sequence		
EL752223_2 AD0012251 Taenia solium UNAM-cd1_adult Taenia solium cDNA, mRNA sequence		
>gi|156076352|gb|ABU46387.1| nuclear elongation factor 1 alpha [Echinococcus felidis]	found 1 time	860
EL757664_2 AD0094004 Taenia solium UNAM-cd1_adult Taenia solium cDNA, mRNA sequence		
>gi|269935947|dbj|BAI49991.1| nuclear elongation factor 1 alpha [Taenia solium]	found 1 time	984
EL751391_3 AD0009129 Taenia solium UNAM-cd1_adult Taenia solium cDNA, mRNA sequence		
**Myophilin**		
>gi|29336625|sp|Q24799.1|MYPH_ECHGR RecName: Full=Myophilin	found 16 times	351
EL762960_3 AD0196013 Taenia solium UNAM-cd1_adult Taenia solium cDNA, mRNA sequence		
EL761553_6 AD0165051 Taenia solium UNAM-cd1_adult Taenia solium cDNA, mRNA sequence		
EL759128_1 AD0117031 Taenia solium UNAM-cd1_adult Taenia solium cDNA, mRNA sequence		
EL758385_2 AD0105004 Taenia solium UNAM-cd1_adult Taenia solium cDNA, mRNA sequence		
EL757444_2 AD0035037 Taenia solium UNAM-cd1_adult Taenia solium cDNA, mRNA sequence		
EL754822_3 AD0023078 Taenia solium UNAM-cd1_adult Taenia solium cDNA, mRNA sequence		
EL751493_5 AD0009231 Taenia solium UNAM-cd1_adult Taenia solium cDNA, mRNA sequence		
EL751248_3 AD0008259 Taenia solium UNAM-cd1_adult Taenia solium cDNA, mRNA sequence		
EL748093_2 LV0333044 Taenia solium UNAM-cd2_larva Taenia solium cDNA, mRNA sequence		
EL746920_3 LV0315062 Taenia solium UNAM-cd2_larva Taenia solium cDNA, mRNA sequence		
EL746009_2 LV0300040 Taenia solium UNAM-cd2_larva Taenia solium cDNA, mRNA sequence		
EL744070_1 LV0267009 Taenia solium UNAM-cd2_larva Taenia solium cDNA, mRNA sequence		
EL743810_3 LV0262051 Taenia solium UNAM-cd2_larva Taenia solium cDNA, mRNA sequence		
EL742827_2 LV0247045 Taenia solium UNAM-cd2_larva Taenia solium cDNA, mRNA sequence		
EL741086_3 LV0220059 Taenia solium UNAM-cd2_larva Taenia solium cDNA, mRNA sequence		
EL741008_2 LV0219058 Taenia solium UNAM-cd2_larva Taenia solium cDNA, mRNA sequence		
>gi|226487430|emb|CAX74585.1| Myophilin [Schistosoma japonicum]	found 3 times	665
EL746109_2 LV0302004 Taenia solium UNAM-cd2_larva Taenia solium cDNA, mRNA sequence		
EL743613_3 LV0260001 Taenia solium UNAM-cd2_larva Taenia solium cDNA, mRNA sequence		
EL743167_1 LV0253014 Taenia solium UNAM-cd2_larva Taenia solium cDNA, mRNA sequence		
**Peptidyl-prolyl cis-trans isomerase**		
>gi|31077167|sp|P14088.2|PPIA_ECHGR RecName: Full=Peptidyl-prolyl cis-trans isomerase;	found 20 times	670
EL763362_3 AD0203007 Taenia solium UNAM-cd1_adult Taenia solium cDNA, mRNA sequence		
EL763184_6 AD0199068 Taenia solium UNAM-cd1_adult Taenia solium cDNA, mRNA sequence		
EL762958_3 AD0196011 Taenia solium UNAM-cd1_adult Taenia solium cDNA, mRNA sequence		
EL759221_2 AD0119021 Taenia solium UNAM-cd1_adult Taenia solium cDNA, mRNA sequence		
EL758571_3 AD0108002 Taenia solium UNAM-cd1_adult Taenia solium cDNA, mRNA sequence		
EL756879_2 AD0031152 Taenia solium UNAM-cd1_adult Taenia solium cDNA, mRNA sequence		
EL756403_2 AD0029198 Taenia solium UNAM-cd1_adult Taenia solium cDNA, mRNA sequence		
EL755438_1 AD0025208 Taenia solium UNAM-cd1_adult Taenia solium cDNA, mRNA sequence		
EL755397_1 AD0025167 Taenia solium UNAM-cd1_adult Taenia solium cDNA, mRNA sequence		
EL753599_2 AD0018137 Taenia solium UNAM-cd1_adult Taenia solium cDNA, mRNA sequence		
EL753353_4 AD0017111 Taenia solium UNAM-cd1_adult Taenia solium cDNA, mRNA sequence		
EL753107_1 AD0016145 Taenia solium UNAM-cd1_adult Taenia solium cDNA, mRNA sequence		
EL752279_6 AD0013046 Taenia solium UNAM-cd1_adult Taenia solium cDNA, mRNA sequence		
EL751661_5 AD0010154 Taenia solium UNAM-cd1_adult Taenia solium cDNA, mRNA sequence		
EL750346_5 AD0005009 Taenia solium UNAM-cd1_adult Taenia solium cDNA, mRNA sequence		
EL750093_1 AD0004008 Taenia solium UNAM-cd1_adult Taenia solium cDNA, mRNA sequence		
EL748497_2 LV0341013 Taenia solium UNAM-cd2_larva Taenia solium cDNA, mRNA sequence		
EL744640_2 LV0277017 Taenia solium UNAM-cd2_larva Taenia solium cDNA, mRNA sequence		
EL743871_2 LV0263040 Taenia solium UNAM-cd2_larva Taenia solium cDNA, mRNA sequence		
EL740903_1 LV0218014 Taenia solium UNAM-cd2_larva Taenia solium cDNA, mRNA sequence		
>gi|295901404|dbj|BAJ07359.1| cyclophilin [Taenia taeniaeformis]	found 2 times	664
EL755318_1 AD0025088 Taenia solium UNAM-cd1_adult Taenia solium cDNA, mRNA sequence		
EL751889_2 AD0011132 Taenia solium UNAM-cd1_adult Taenia solium cDNA, mRNA sequence		
>gi|156365561|ref|XP_001626713.1| predicted protein [Nematostella vectensis]	found 1 time	373
EL745512_2 LV0292046 Taenia solium UNAM-cd2_larva Taenia solium cDNA, mRNA sequence		
>gi|336371514|gb|EGN99853.1| hypothetical protein SERLA73DRAFT_53209 [Serpula lacrymans var. lacrymans S7.3]	found 1 time	373
GT893213_3 TSBM.R27.esd Taenia solium cysticerca cDNA library Taenia solium cDNA, mRNA sequence		
>gi|327292088|ref|XP_003230752.1| PREDICTED: peptidyl-prolyl cis-trans isomerase NIMA-interacting 4-like, partial [Anolis carolinensis]	found 1 time	246
EL745527_1 LV0293005 Taenia solium UNAM-cd2_larva Taenia solium cDNA, mRNA sequence		
**Cu/Zn Superoxide dismutase**		
>gi|347948498|pdb|3MND|A Chain A, Crystallographic Analysis Of The Cytosolic CuZN SUPEROXIDE DISMUTASE From Taenia Solium	found 2 times	483
EL762847_4 AD0194018 Taenia solium UNAM-cd1_adult Taenia solium cDNA, mRNA sequence		
EL758484_3 AD0106027 Taenia solium UNAM-cd1_adult Taenia solium cDNA, mRNA sequence		
**Cytosolic fatty acid binding protein**		
>gi|82412213|gb|ABB76135.1| cytosolic fatty acid binding protein [Taenia solium]	found 352 times	520
EL763189_4 AD0200004 Taenia solium UNAM-cd1_adult Taenia solium cDNA, mRNA sequence		
EL763167_2 AD0199051 Taenia solium UNAM-cd1_adult Taenia solium cDNA, mRNA sequence		
EL762913_1 AD0195033 Taenia solium UNAM-cd1_adult Taenia solium cDNA, mRNA sequence		
EL762718_1 AD0192016 Taenia solium UNAM-cd1_adult Taenia solium cDNA, mRNA sequence		
EL762683_1 AD0191044 Taenia solium UNAM-cd1_adult Taenia solium cDNA, mRNA sequence		
EL762674_2 AD0191035 Taenia solium UNAM-cd1_adult Taenia solium cDNA, mRNA sequence		
EL762661_3 AD0191022 Taenia solium UNAM-cd1_adult Taenia solium cDNA, mRNA sequence		
EL762488_1 AD0187007 Taenia solium UNAM-cd1_adult Taenia solium cDNA, mRNA sequence		
EL762403_6 AD0185008 Taenia solium UNAM-cd1_adult Taenia solium cDNA, mRNA sequence		
EL762344_1 AD0183045 Taenia solium UNAM-cd1_adult Taenia solium cDNA, mRNA sequence		

Table 1: Continued.

**Protein representing protein group**<br>>Protein identified by ESTs<br>EST	number of ESTs linked to protein	TBLASTN score
EL762080_5 AD0178014 Taenia solium UNAM-cd1_adult Taenia solium cDNA, mRNA sequence		
EL761995_4 AD0176001 Taenia solium UNAM-cd1_adult Taenia solium cDNA, mRNA sequence		
EL761822_4 AD0171039 Taenia solium UNAM-cd1_adult Taenia solium cDNA, mRNA sequence		
EL761722_4 AD0169039 Taenia solium UNAM-cd1_adult Taenia solium cDNA, mRNA sequence		
EL761674_4 AD0168034 Taenia solium UNAM-cd1_adult Taenia solium cDNA, mRNA sequence		
EL761513_4 AD0165011 Taenia solium UNAM-cd1_adult Taenia solium cDNA, mRNA sequence		
EL761394_4 AD0163003 Taenia solium UNAM-cd1_adult Taenia solium cDNA, mRNA sequence		
EL761039_6 AD0155027 Taenia solium UNAM-cd1_adult Taenia solium cDNA, mRNA sequence		
EL760799_2 AD0149015 Taenia solium UNAM-cd1_adult Taenia solium cDNA, mRNA sequence		
EL760749_2 AD0148027 Taenia solium UNAM-cd1_adult Taenia solium cDNA, mRNA sequence		
EL760714_2 AD0147046 Taenia solium UNAM-cd1_adult Taenia solium cDNA, mRNA sequence		
EL760708_1 AD0147040 Taenia solium UNAM-cd1_adult Taenia solium cDNA, mRNA sequence		
EL760698_2 AD0147030 Taenia solium UNAM-cd1_adult Taenia solium cDNA, mRNA sequence		
EL760634_2 AD0146019 Taenia solium UNAM-cd1_adult Taenia solium cDNA, mRNA sequence		
EL760553_1 AD0144040 Taenia solium UNAM-cd1_adult Taenia solium cDNA, mRNA sequence		
EL760535_2 AD0144022 Taenia solium UNAM-cd1_adult Taenia solium cDNA, mRNA sequence		
EL760522_2 AD0144009 Taenia solium UNAM-cd1_adult Taenia solium cDNA, mRNA sequence		
EL760502_3 AD0143044 Taenia solium UNAM-cd1_adult Taenia solium cDNA, mRNA sequence		
EL760469_2 AD0143011 Taenia solium UNAM-cd1_adult Taenia solium cDNA, mRNA sequence		
EL760439_1 AD0142037 Taenia solium UNAM-cd1_adult Taenia solium cDNA, mRNA sequence		
EL760426_1 AD0142024 Taenia solium UNAM-cd1_adult Taenia solium cDNA, mRNA sequence		
EL760425_1 AD0142023 Taenia solium UNAM-cd1_adult Taenia solium cDNA, mRNA sequence		
EL760372_2 AD0141009 Taenia solium UNAM-cd1_adult Taenia solium cDNA, mRNA sequence		
EL760342_1 AD0140023 Taenia solium UNAM-cd1_adult Taenia solium cDNA, mRNA sequence		
EL760296_2 AD0139032 Taenia solium UNAM-cd1_adult Taenia solium cDNA, mRNA sequence		
EL760274_2 AD0139010 Taenia solium UNAM-cd1_adult Taenia solium cDNA, mRNA sequence		
EL760189_2 AD0137022 Taenia solium UNAM-cd1_adult Taenia solium cDNA, mRNA sequence		
EL760183_3 AD0137016 Taenia solium UNAM-cd1_adult Taenia solium cDNA, mRNA sequence		
EL760101_1 AD0135047 Taenia solium UNAM-cd1_adult Taenia solium cDNA, mRNA sequence		
EL760090_1 AD0135036 Taenia solium UNAM-cd1_adult Taenia solium cDNA, mRNA sequence		
EL760056_1 AD0135002 Taenia solium UNAM-cd1_adult Taenia solium cDNA, mRNA sequence		
EL760027_3 AD0134020 Taenia solium UNAM-cd1_adult Taenia solium cDNA, mRNA sequence		
EL760007_2 AD0133052 Taenia solium UNAM-cd1_adult Taenia solium cDNA, mRNA sequence		
EL759995_2 AD0133040 Taenia solium UNAM-cd1_adult Taenia solium cDNA, mRNA sequence		
EL759990_2 AD0133035 Taenia solium UNAM-cd1_adult Taenia solium cDNA, mRNA sequence		
EL759980_2 AD0133025 Taenia solium UNAM-cd1_adult Taenia solium cDNA, mRNA sequence		
EL759974_2 AD0133019 Taenia solium UNAM-cd1_adult Taenia solium cDNA, mRNA sequence		
EL759842_2 AD0130032 Taenia solium UNAM-cd1_adult Taenia solium cDNA, mRNA sequence		
EL759824_2 AD0130014 Taenia solium UNAM-cd1_adult Taenia solium cDNA, mRNA sequence		
EL759810_3 AD0129050 Taenia solium UNAM-cd1_adult Taenia solium cDNA, mRNA sequence		
EL759770_1 AD0129010 Taenia solium UNAM-cd1_adult Taenia solium cDNA, mRNA sequence		
EL759713_2 AD0128002 Taenia solium UNAM-cd1_adult Taenia solium cDNA, mRNA sequence		
EL759707_3 AD0127048 Taenia solium UNAM-cd1_adult Taenia solium cDNA, mRNA sequence		
EL759681_2 AD0127022 Taenia solium UNAM-cd1_adult Taenia solium cDNA, mRNA sequence		
EL759649_1 AD0126046 Taenia solium UNAM-cd1_adult Taenia solium cDNA, mRNA sequence		
EL759635_2 AD0126032 Taenia solium UNAM-cd1_adult Taenia solium cDNA, mRNA sequence		
EL759624_3 AD0126021 Taenia solium UNAM-cd1_adult Taenia solium cDNA, mRNA sequence		
EL759621_3 AD0126018 Taenia solium UNAM-cd1_adult Taenia solium cDNA, mRNA sequence		
EL759611_3 AD0126008 Taenia solium UNAM-cd1_adult Taenia solium cDNA, mRNA sequence		
EL759560_3 AD0125018 Taenia solium UNAM-cd1_adult Taenia solium cDNA, mRNA sequence		
EL759518_3 AD0124032 Taenia solium UNAM-cd1_adult Taenia solium cDNA, mRNA sequence		
EL759444_2 AD0123023 Taenia solium UNAM-cd1_adult Taenia solium cDNA, mRNA sequence		
EL759411_3 AD0122055 Taenia solium UNAM-cd1_adult Taenia solium cDNA, mRNA sequence		
EL759408_3 AD0122052 Taenia solium UNAM-cd1_adult Taenia solium cDNA, mRNA sequence		
EL759354_1 AD0121050 Taenia solium UNAM-cd1_adult Taenia solium cDNA, mRNA sequence		
EL758522_2 AD0107015 Taenia solium UNAM-cd1_adult Taenia solium cDNA, mRNA sequence		
EL758260_3 AD0103004 Taenia solium UNAM-cd1_adult Taenia solium cDNA, mRNA sequence		
EL758211_2 AD0102022 Taenia solium UNAM-cd1_adult Taenia solium cDNA, mRNA sequence		
EL758082_1 AD0100027 Taenia solium UNAM-cd1_adult Taenia solium cDNA, mRNA sequence		
EL757815_1 AD0096018 Taenia solium UNAM-cd1_adult Taenia solium cDNA, mRNA sequence		
EL757645_3 AD0035238 Taenia solium UNAM-cd1_adult Taenia solium cDNA, mRNA sequence		
EL757633_2 AD0035226 Taenia solium UNAM-cd1_adult Taenia solium cDNA, mRNA sequence		
EL757520_3 AD0035113 Taenia solium UNAM-cd1_adult Taenia solium cDNA, mRNA sequence		
EL757517_2 AD0035110 Taenia solium UNAM-cd1_adult Taenia solium cDNA, mRNA sequence		
EL757502_3 AD0035095 Taenia solium UNAM-cd1_adult Taenia solium cDNA, mRNA sequence		
EL757450_1 AD0035043 Taenia solium UNAM-cd1_adult Taenia solium cDNA, mRNA sequence		
EL757447_3 AD0035040 Taenia solium UNAM-cd1_adult Taenia solium cDNA, mRNA sequence		
EL757407_3 AD0034223 Taenia solium UNAM-cd1_adult Taenia solium cDNA, mRNA sequence		
EL757401_2 AD0034217 Taenia solium UNAM-cd1_adult Taenia solium cDNA, mRNA sequence		
EL757220_2 AD0034036 Taenia solium UNAM-cd1_adult Taenia solium cDNA, mRNA sequence		
EL757215_2 AD0034031 Taenia solium UNAM-cd1_adult Taenia solium cDNA, mRNA sequence		
EL757195_2 AD0034011 Taenia solium UNAM-cd1_adult Taenia solium cDNA, mRNA sequence		
EL757139_2 AD0033001 Taenia solium UNAM-cd1_adult Taenia solium cDNA, mRNA sequence		
EL757036_2 AD0032048 Taenia solium UNAM-cd1_adult Taenia solium cDNA, mRNA sequence		
EL757015_2 AD0032027 Taenia solium UNAM-cd1_adult Taenia solium cDNA, mRNA sequence		
EL756883_2 AD0031156 Taenia solium UNAM-cd1_adult Taenia solium cDNA, mRNA sequence		
EL756863_3 AD0031136 Taenia solium UNAM-cd1_adult Taenia solium cDNA, mRNA sequence		
EL756841_3 AD0031114 Taenia solium UNAM-cd1_adult Taenia solium cDNA, mRNA sequence		
EL756759_2 AD0031032 Taenia solium UNAM-cd1_adult Taenia solium cDNA, mRNA sequence		
EL756715_2 AD0030247 Taenia solium UNAM-cd1_adult Taenia solium cDNA, mRNA sequence		
EL756699_3 AD0030231 Taenia solium UNAM-cd1_adult Taenia solium cDNA, mRNA sequence		
EL756662_2 AD0030194 Taenia solium UNAM-cd1_adult Taenia solium cDNA, mRNA sequence		

Table 1: Continued.

**Protein representing protein group**<br>>Protein identified by ESTs<br>EST	number of ESTs linked to protein	TBLASTN score
EL756656_2 AD0030188 Taenia solium UNAM-cd1_adult Taenia solium cDNA, mRNA sequence		
EL756641_3 AD0030173 Taenia solium UNAM-cd1_adult Taenia solium cDNA, mRNA sequence		
EL756628_3 AD0030160 Taenia solium UNAM-cd1_adult Taenia solium cDNA, mRNA sequence		
EL756598_2 AD0030130 Taenia solium UNAM-cd1_adult Taenia solium cDNA, mRNA sequence		
EL756530_3 AD0030062 Taenia solium UNAM-cd1_adult Taenia solium cDNA, mRNA sequence		
EL756514_2 AD0030046 Taenia solium UNAM-cd1_adult Taenia solium cDNA, mRNA sequence		
EL756489_2 AD0030021 Taenia solium UNAM-cd1_adult Taenia solium cDNA, mRNA sequence		
EL756450_1 AD0029245 Taenia solium UNAM-cd1_adult Taenia solium cDNA, mRNA sequence		
EL756425_2 AD0029220 Taenia solium UNAM-cd1_adult Taenia solium cDNA, mRNA sequence		
EL756336_1 AD0029131 Taenia solium UNAM-cd1_adult Taenia solium cDNA, mRNA sequence		
EL756305_3 AD0029100 Taenia solium UNAM-cd1_adult Taenia solium cDNA, mRNA sequence		
EL756295_3 AD0029090 Taenia solium UNAM-cd1_adult Taenia solium cDNA, mRNA sequence		
EL756244_3 AD0029039 Taenia solium UNAM-cd1_adult Taenia solium cDNA, mRNA sequence		
EL756201_3 AD0028259 Taenia solium UNAM-cd1_adult Taenia solium cDNA, mRNA sequence		
EL756138_3 AD0028196 Taenia solium UNAM-cd1_adult Taenia solium cDNA, mRNA sequence		
EL756102_2 AD0028160 Taenia solium UNAM-cd1_adult Taenia solium cDNA, mRNA sequence		
EL756078_1 AD0028136 Taenia solium UNAM-cd1_adult Taenia solium cDNA, mRNA sequence		
EL756058_2 AD0028116 Taenia solium UNAM-cd1_adult Taenia solium cDNA, mRNA sequence		
EL756048_3 AD0028106 Taenia solium UNAM-cd1_adult Taenia solium cDNA, mRNA sequence		
EL756044_3 AD0028102 Taenia solium UNAM-cd1_adult Taenia solium cDNA, mRNA sequence		
EL756011_3 AD0028069 Taenia solium UNAM-cd1_adult Taenia solium cDNA, mRNA sequence		
EL756008_2 AD0028066 Taenia solium UNAM-cd1_adult Taenia solium cDNA, mRNA sequence		
EL755972_3 AD0028030 Taenia solium UNAM-cd1_adult Taenia solium cDNA, mRNA sequence		
EL755955_2 AD0028013 Taenia solium UNAM-cd1_adult Taenia solium cDNA, mRNA sequence		
EL755881_3 AD0027177 Taenia solium UNAM-cd1_adult Taenia solium cDNA, mRNA sequence		
EL755878_3 AD0027174 Taenia solium UNAM-cd1_adult Taenia solium cDNA, mRNA sequence		
EL755821_2 AD0027117 Taenia solium UNAM-cd1_adult Taenia solium cDNA, mRNA sequence		
EL755754_1 AD0027050 Taenia solium UNAM-cd1_adult Taenia solium cDNA, mRNA sequence		
EL755720_2 AD0027016 Taenia solium UNAM-cd1_adult Taenia solium cDNA, mRNA sequence		
EL755666_2 AD0026189 Taenia solium UNAM-cd1_adult Taenia solium cDNA, mRNA sequence		
EL755603_2 AD0026126 Taenia solium UNAM-cd1_adult Taenia solium cDNA, mRNA sequence		
EL755602_2 AD0026125 Taenia solium UNAM-cd1_adult Taenia solium cDNA, mRNA sequence		
EL755488_1 AD0026011 Taenia solium UNAM-cd1_adult Taenia solium cDNA, mRNA sequence		
EL755475_3 AD0025245 Taenia solium UNAM-cd1_adult Taenia solium cDNA, mRNA sequence		
EL755449_2 AD0025219 Taenia solium UNAM-cd1_adult Taenia solium cDNA, mRNA sequence		
EL755437_2 AD0025207 Taenia solium UNAM-cd1_adult Taenia solium cDNA, mRNA sequence		
EL755416_2 AD0025186 Taenia solium UNAM-cd1_adult Taenia solium cDNA, mRNA sequence		
EL755380_2 AD0025150 Taenia solium UNAM-cd1_adult Taenia solium cDNA, mRNA sequence		
EL755368_2 AD0025138 Taenia solium UNAM-cd1_adult Taenia solium cDNA, mRNA sequence		
EL755330_2 AD0025100 Taenia solium UNAM-cd1_adult Taenia solium cDNA, mRNA sequence		
EL755312_3 AD0025082 Taenia solium UNAM-cd1_adult Taenia solium cDNA, mRNA sequence		
EL755309_3 AD0025079 Taenia solium UNAM-cd1_adult Taenia solium cDNA, mRNA sequence		
EL755268_1 AD0025038 Taenia solium UNAM-cd1_adult Taenia solium cDNA, mRNA sequence		
EL755224_2 AD0024241 Taenia solium UNAM-cd1_adult Taenia solium cDNA, mRNA sequence		
EL755157_2 AD0024174 Taenia solium UNAM-cd1_adult Taenia solium cDNA, mRNA sequence		
EL755139_2 AD0024156 Taenia solium UNAM-cd1_adult Taenia solium cDNA, mRNA sequence		
EL755089_1 AD0024106 Taenia solium UNAM-cd1_adult Taenia solium cDNA, mRNA sequence		
EL755049_3 AD0024066 Taenia solium UNAM-cd1_adult Taenia solium cDNA, mRNA sequence		
EL755044_1 AD0024061 Taenia solium UNAM-cd1_adult Taenia solium cDNA, mRNA sequence		
EL755033_2 AD0024050 Taenia solium UNAM-cd1_adult Taenia solium cDNA, mRNA sequence		
EL755016_2 AD0024033 Taenia solium UNAM-cd1_adult Taenia solium cDNA, mRNA sequence		
EL754959_2 AD0023215 Taenia solium UNAM-cd1_adult Taenia solium cDNA, mRNA sequence		
EL754952_2 AD0023208 Taenia solium UNAM-cd1_adult Taenia solium cDNA, mRNA sequence		
EL754925_2 AD0023181 Taenia solium UNAM-cd1_adult Taenia solium cDNA, mRNA sequence		
EL754883_2 AD0023139 Taenia solium UNAM-cd1_adult Taenia solium cDNA, mRNA sequence		
EL754881_2 AD0023137 Taenia solium UNAM-cd1_adult Taenia solium cDNA, mRNA sequence		
EL754859_3 AD0023115 Taenia solium UNAM-cd1_adult Taenia solium cDNA, mRNA sequence		
EL754828_2 AD0023084 Taenia solium UNAM-cd1_adult Taenia solium cDNA, mRNA sequence		
EL754782_2 AD0023038 Taenia solium UNAM-cd1_adult Taenia solium cDNA, mRNA sequence		
EL754773_2 AD0023029 Taenia solium UNAM-cd1_adult Taenia solium cDNA, mRNA sequence		
EL754746_1 AD0023002 Taenia solium UNAM-cd1_adult Taenia solium cDNA, mRNA sequence		
EL754738_2 AD0022263 Taenia solium UNAM-cd1_adult Taenia solium cDNA, mRNA sequence		
EL754703_2 AD0022228 Taenia solium UNAM-cd1_adult Taenia solium cDNA, mRNA sequence		
EL754645_2 AD0022170 Taenia solium UNAM-cd1_adult Taenia solium cDNA, mRNA sequence		
EL754629_2 AD0022154 Taenia solium UNAM-cd1_adult Taenia solium cDNA, mRNA sequence		
EL754571_2 AD0022096 Taenia solium UNAM-cd1_adult Taenia solium cDNA, mRNA sequence		
EL754562_2 AD0022087 Taenia solium UNAM-cd1_adult Taenia solium cDNA, mRNA sequence		
EL754549_2 AD0022074 Taenia solium UNAM-cd1_adult Taenia solium cDNA, mRNA sequence		
EL754541_2 AD0022066 Taenia solium UNAM-cd1_adult Taenia solium cDNA, mRNA sequence		
EL754508_1 AD0022033 Taenia solium UNAM-cd1_adult Taenia solium cDNA, mRNA sequence		
EL754444_2 AD0021234 Taenia solium UNAM-cd1_adult Taenia solium cDNA, mRNA sequence		
EL754371_2 AD0021161 Taenia solium UNAM-cd1_adult Taenia solium cDNA, mRNA sequence		
EL754354_2 AD0021144 Taenia solium UNAM-cd1_adult Taenia solium cDNA, mRNA sequence		
EL754340_2 AD0021130 Taenia solium UNAM-cd1_adult Taenia solium cDNA, mRNA sequence		
EL754302_2 AD0021092 Taenia solium UNAM-cd1_adult Taenia solium cDNA, mRNA sequence		
EL754256_2 AD0021046 Taenia solium UNAM-cd1_adult Taenia solium cDNA, mRNA sequence		
EL754248_2 AD0021038 Taenia solium UNAM-cd1_adult Taenia solium cDNA, mRNA sequence		
EL754247_2 AD0021037 Taenia solium UNAM-cd1_adult Taenia solium cDNA, mRNA sequence		
EL754212_2 AD0021002 Taenia solium UNAM-cd1_adult Taenia solium cDNA, mRNA sequence		
EL754117_2 AD0020162 Taenia solium UNAM-cd1_adult Taenia solium cDNA, mRNA sequence		
EL754105_2 AD0020150 Taenia solium UNAM-cd1_adult Taenia solium cDNA, mRNA sequence		
EL754104_2 AD0020149 Taenia solium UNAM-cd1_adult Taenia solium cDNA, mRNA sequence		

Table 1: Continued.

**Protein representing protein group**<br>>Protein identified by ESTs<br>EST	number of ESTs linked to protein	TBLASTN score
EL754083_2 AD0020128 Taenia solium UNAM-cd1_adult Taenia solium cDNA, mRNA sequence		
EL754002_2 AD0020047 Taenia solium UNAM-cd1_adult Taenia solium cDNA, mRNA sequence		
EL753993_2 AD0020038 Taenia solium UNAM-cd1_adult Taenia solium cDNA, mRNA sequence		
EL753944_2 AD0019231 Taenia solium UNAM-cd1_adult Taenia solium cDNA, mRNA sequence		
EL753934_2 AD0019221 Taenia solium UNAM-cd1_adult Taenia solium cDNA, mRNA sequence		
EL753869_1 AD0019156 Taenia solium UNAM-cd1_adult Taenia solium cDNA, mRNA sequence		
EL753857_2 AD0019144 Taenia solium UNAM-cd1_adult Taenia solium cDNA, mRNA sequence		
EL753824_2 AD0019111 Taenia solium UNAM-cd1_adult Taenia solium cDNA, mRNA sequence		
EL753808_2 AD0019095 Taenia solium UNAM-cd1_adult Taenia solium cDNA, mRNA sequence		
EL753770_2 AD0019057 Taenia solium UNAM-cd1_adult Taenia solium cDNA, mRNA sequence		
EL753764_2 AD0019051 Taenia solium UNAM-cd1_adult Taenia solium cDNA, mRNA sequence		
EL753756_2 AD0019043 Taenia solium UNAM-cd1_adult Taenia solium cDNA, mRNA sequence		
EL753736_3 AD0019023 Taenia solium UNAM-cd1_adult Taenia solium cDNA, mRNA sequence		
EL753684_2 AD0018222 Taenia solium UNAM-cd1_adult Taenia solium cDNA, mRNA sequence		
EL753674_2 AD0018212 Taenia solium UNAM-cd1_adult Taenia solium cDNA, mRNA sequence		
EL753657_2 AD0018195 Taenia solium UNAM-cd1_adult Taenia solium cDNA, mRNA sequence		
EL753645_2 AD0018183 Taenia solium UNAM-cd1_adult Taenia solium cDNA, mRNA sequence		
EL753602_2 AD0018140 Taenia solium UNAM-cd1_adult Taenia solium cDNA, mRNA sequence		
EL753557_2 AD0018095 Taenia solium UNAM-cd1_adult Taenia solium cDNA, mRNA sequence		
EL753538_2 AD0018076 Taenia solium UNAM-cd1_adult Taenia solium cDNA, mRNA sequence		
EL753505_2 AD0018043 Taenia solium UNAM-cd1_adult Taenia solium cDNA, mRNA sequence		
EL753468_2 AD0018006 Taenia solium UNAM-cd1_adult Taenia solium cDNA, mRNA sequence		
EL753453_6 AD0017211 Taenia solium UNAM-cd1_adult Taenia solium cDNA, mRNA sequence		
EL753380_4 AD0017138 Taenia solium UNAM-cd1_adult Taenia solium cDNA, mRNA sequence		
EL753308_5 AD0017066 Taenia solium UNAM-cd1_adult Taenia solium cDNA, mRNA sequence		
EL753273_6 AD0017031 Taenia solium UNAM-cd1_adult Taenia solium cDNA, mRNA sequence		
EL753266_6 AD0017024 Taenia solium UNAM-cd1_adult Taenia solium cDNA, mRNA sequence		
EL753257_5 AD0017015 Taenia solium UNAM-cd1_adult Taenia solium cDNA, mRNA sequence		
EL753247_4 AD0017005 Taenia solium UNAM-cd1_adult Taenia solium cDNA, mRNA sequence		
EL753244_4 AD0017002 Taenia solium UNAM-cd1_adult Taenia solium cDNA, mRNA sequence		
EL753230_2 AD0016268 Taenia solium UNAM-cd1_adult Taenia solium cDNA, mRNA sequence		
EL753140_2 AD0016178 Taenia solium UNAM-cd1_adult Taenia solium cDNA, mRNA sequence		
EL753120_3 AD0016158 Taenia solium UNAM-cd1_adult Taenia solium cDNA, mRNA sequence		
EL753049_3 AD0016087 Taenia solium UNAM-cd1_adult Taenia solium cDNA, mRNA sequence		
EL753035_2 AD0016073 Taenia solium UNAM-cd1_adult Taenia solium cDNA, mRNA sequence		
EL753003_3 AD0016041 Taenia solium UNAM-cd1_adult Taenia solium cDNA, mRNA sequence		
EL752995_3 AD0016033 Taenia solium UNAM-cd1_adult Taenia solium cDNA, mRNA sequence		
EL752965_2 AD0016003 Taenia solium UNAM-cd1_adult Taenia solium cDNA, mRNA sequence		
EL752836_4 AD0015095 Taenia solium UNAM-cd1_adult Taenia solium cDNA, mRNA sequence		
EL752826_4 AD0015085 Taenia solium UNAM-cd1_adult Taenia solium cDNA, mRNA sequence		
EL752786_5 AD0015045 Taenia solium UNAM-cd1_adult Taenia solium cDNA, mRNA sequence		
EL752751_5 AD0015010 Taenia solium UNAM-cd1_adult Taenia solium cDNA, mRNA sequence		
EL752743_5 AD0015002 Taenia solium UNAM-cd1_adult Taenia solium cDNA, mRNA sequence		
EL752701_3 AD0014242 Taenia solium UNAM-cd1_adult Taenia solium cDNA, mRNA sequence		
EL752602_2 AD0014143 Taenia solium UNAM-cd1_adult Taenia solium cDNA, mRNA sequence		
EL752570_2 AD0014111 Taenia solium UNAM-cd1_adult Taenia solium cDNA, mRNA sequence		
EL752518_2 AD0014059 Taenia solium UNAM-cd1_adult Taenia solium cDNA, mRNA sequence		
EL752462_2 AD0014003 Taenia solium UNAM-cd1_adult Taenia solium cDNA, mRNA sequence		
EL752378_6 AD0013145 Taenia solium UNAM-cd1_adult Taenia solium cDNA, mRNA sequence		
EL752260_4 AD0013027 Taenia solium UNAM-cd1_adult Taenia solium cDNA, mRNA sequence		
EL752259_4 AD0013026 Taenia solium UNAM-cd1_adult Taenia solium cDNA, mRNA sequence		
EL752240_6 AD0013007 Taenia solium UNAM-cd1_adult Taenia solium cDNA, mRNA sequence		
EL752139_2 AD0012167 Taenia solium UNAM-cd1_adult Taenia solium cDNA, mRNA sequence		
EL752004_2 AD0012032 Taenia solium UNAM-cd1_adult Taenia solium cDNA, mRNA sequence		
EL751983_3 AD0012011 Taenia solium UNAM-cd1_adult Taenia solium cDNA, mRNA sequence		
EL751977_3 AD0012005 Taenia solium UNAM-cd1_adult Taenia solium cDNA, mRNA sequence		
EL751955_5 AD0011198 Taenia solium UNAM-cd1_adult Taenia solium cDNA, mRNA sequence		
EL751923_6 AD0011166 Taenia solium UNAM-cd1_adult Taenia solium cDNA, mRNA sequence		
EL751911_6 AD0011154 Taenia solium UNAM-cd1_adult Taenia solium cDNA, mRNA sequence		
EL751907_6 AD0011150 Taenia solium UNAM-cd1_adult Taenia solium cDNA, mRNA sequence		
EL751884_6 AD0011127 Taenia solium UNAM-cd1_adult Taenia solium cDNA, mRNA sequence		
EL751819_4 AD0011062 Taenia solium UNAM-cd1_adult Taenia solium cDNA, mRNA sequence		
EL751813_6 AD0011056 Taenia solium UNAM-cd1_adult Taenia solium cDNA, mRNA sequence		
EL751774_4 AD0011017 Taenia solium UNAM-cd1_adult Taenia solium cDNA, mRNA sequence		
EL751728_3 AD0010221 Taenia solium UNAM-cd1_adult Taenia solium cDNA, mRNA sequence		
EL751696_2 AD0010189 Taenia solium UNAM-cd1_adult Taenia solium cDNA, mRNA sequence		
EL751684_3 AD0010177 Taenia solium UNAM-cd1_adult Taenia solium cDNA, mRNA sequence		
EL751681_3 AD0010174 Taenia solium UNAM-cd1_adult Taenia solium cDNA, mRNA sequence		
EL751680_2 AD0010173 Taenia solium UNAM-cd1_adult Taenia solium cDNA, mRNA sequence		
EL751657_1 AD0010150 Taenia solium UNAM-cd1_adult Taenia solium cDNA, mRNA sequence		
EL751647_1 AD0010140 Taenia solium UNAM-cd1_adult Taenia solium cDNA, mRNA sequence		
EL751581_1 AD0010074 Taenia solium UNAM-cd1_adult Taenia solium cDNA, mRNA sequence		
EL751573_2 AD0010066 Taenia solium UNAM-cd1_adult Taenia solium cDNA, mRNA sequence		
EL751477_6 AD0009215 Taenia solium UNAM-cd1_adult Taenia solium cDNA, mRNA sequence		
EL751460_5 AD0009198 Taenia solium UNAM-cd1_adult Taenia solium cDNA, mRNA sequence		
EL751442_5 AD0009180 Taenia solium UNAM-cd1_adult Taenia solium cDNA, mRNA sequence		
EL751413_5 AD0009151 Taenia solium UNAM-cd1_adult Taenia solium cDNA, mRNA sequence		
EL751411_4 AD0009149 Taenia solium UNAM-cd1_adult Taenia solium cDNA, mRNA sequence		
EL751409_6 AD0009147 Taenia solium UNAM-cd1_adult Taenia solium cDNA, mRNA sequence		
EL751342_4 AD0009080 Taenia solium UNAM-cd1_adult Taenia solium cDNA, mRNA sequence		
EL751286_6 AD0009024 Taenia solium UNAM-cd1_adult Taenia solium cDNA, mRNA sequence		
EL751276_4 AD0009014 Taenia solium UNAM-cd1_adult Taenia solium cDNA, mRNA sequence		

Table 1: Continued.

**Protein representing protein group**<br>>Protein identified by ESTs<br>EST	number of ESTs linked to protein	TBLASTN score
EL751230_2 AD0008241 Taenia solium UNAM-cd1_adult Taenia solium cDNA, mRNA sequence		
EL751211_3 AD0008222 Taenia solium UNAM-cd1_adult Taenia solium cDNA, mRNA sequence		
EL751160_3 AD0008171 Taenia solium UNAM-cd1_adult Taenia solium cDNA, mRNA sequence		
EL751159_2 AD0008170 Taenia solium UNAM-cd1_adult Taenia solium cDNA, mRNA sequence		
EL751157_2 AD0008168 Taenia solium UNAM-cd1_adult Taenia solium cDNA, mRNA sequence		
EL751087_2 AD0008098 Taenia solium UNAM-cd1_adult Taenia solium cDNA, mRNA sequence		
EL751018_2 AD0008029 Taenia solium UNAM-cd1_adult Taenia solium cDNA, mRNA sequence		
EL751002_3 AD0008013 Taenia solium UNAM-cd1_adult Taenia solium cDNA, mRNA sequence		
EL750977_5 AD0007213 Taenia solium UNAM-cd1_adult Taenia solium cDNA, mRNA sequence		
EL750963_4 AD0007199 Taenia solium UNAM-cd1_adult Taenia solium cDNA, mRNA sequence		
EL750946_6 AD0007182 Taenia solium UNAM-cd1_adult Taenia solium cDNA, mRNA sequence		
EL750931_6 AD0007167 Taenia solium UNAM-cd1_adult Taenia solium cDNA, mRNA sequence		
EL750907_1 AD0007143 Taenia solium UNAM-cd1_adult Taenia solium cDNA, mRNA sequence		
EL750836_5 AD0007072 Taenia solium UNAM-cd1_adult Taenia solium cDNA, mRNA sequence		
EL750828_4 AD0007064 Taenia solium UNAM-cd1_adult Taenia solium cDNA, mRNA sequence		
EL750811_5 AD0007047 Taenia solium UNAM-cd1_adult Taenia solium cDNA, mRNA sequence		
EL750770_5 AD0007006 Taenia solium UNAM-cd1_adult Taenia solium cDNA, mRNA sequence		
EL750752_2 AD0006191 Taenia solium UNAM-cd1_adult Taenia solium cDNA, mRNA sequence		
EL750735_2 AD0006174 Taenia solium UNAM-cd1_adult Taenia solium cDNA, mRNA sequence		
EL750716_2 AD0006155 Taenia solium UNAM-cd1_adult Taenia solium cDNA, mRNA sequence		
EL750605_1 AD0006044 Taenia solium UNAM-cd1_adult Taenia solium cDNA, mRNA sequence		
EL750601_1 AD0006040 Taenia solium UNAM-cd1_adult Taenia solium cDNA, mRNA sequence		
EL750567_2 AD0006006 Taenia solium UNAM-cd1_adult Taenia solium cDNA, mRNA sequence		
EL750490_5 AD0005153 Taenia solium UNAM-cd1_adult Taenia solium cDNA, mRNA sequence		
EL750478_4 AD0005141 Taenia solium UNAM-cd1_adult Taenia solium cDNA, mRNA sequence		
EL750443_4 AD0005106 Taenia solium UNAM-cd1_adult Taenia solium cDNA, mRNA sequence		
EL750433_4 AD0005096 Taenia solium UNAM-cd1_adult Taenia solium cDNA, mRNA sequence		
EL750422_4 AD0005085 Taenia solium UNAM-cd1_adult Taenia solium cDNA, mRNA sequence		
EL750414_6 AD0005077 Taenia solium UNAM-cd1_adult Taenia solium cDNA, mRNA sequence		
EL750412_5 AD0005075 Taenia solium UNAM-cd1_adult Taenia solium cDNA, mRNA sequence		
EL750383_6 AD0005046 Taenia solium UNAM-cd1_adult Taenia solium cDNA, mRNA sequence		
EL750357_5 AD0005020 Taenia solium UNAM-cd1_adult Taenia solium cDNA, mRNA sequence		
EL750345_5 AD0005008 Taenia solium UNAM-cd1_adult Taenia solium cDNA, mRNA sequence		
EL750342_5 AD0005005 Taenia solium UNAM-cd1_adult Taenia solium cDNA, mRNA sequence		
EL750341_6 AD0005004 Taenia solium UNAM-cd1_adult Taenia solium cDNA, mRNA sequence		
EL750339_5 AD0005002 Taenia solium UNAM-cd1_adult Taenia solium cDNA, mRNA sequence		
EL750237_3 AD0004152 Taenia solium UNAM-cd1_adult Taenia solium cDNA, mRNA sequence		
EL750198_3 AD0004113 Taenia solium UNAM-cd1_adult Taenia solium cDNA, mRNA sequence		
EL750184_3 AD0004099 Taenia solium UNAM-cd1_adult Taenia solium cDNA, mRNA sequence		
EL750175_3 AD0004090 Taenia solium UNAM-cd1_adult Taenia solium cDNA, mRNA sequence		
EL750169_1 AD0004084 Taenia solium UNAM-cd1_adult Taenia solium cDNA, mRNA sequence		
EL750167_2 AD0004082 Taenia solium UNAM-cd1_adult Taenia solium cDNA, mRNA sequence		
EL750137_2 AD0004052 Taenia solium UNAM-cd1_adult Taenia solium cDNA, mRNA sequence		
EL750105_2 AD0004020 Taenia solium UNAM-cd1_adult Taenia solium cDNA, mRNA sequence		
EL750092_1 AD0004007 Taenia solium UNAM-cd1_adult Taenia solium cDNA, mRNA sequence		
EL750089_1 AD0004004 Taenia solium UNAM-cd1_adult Taenia solium cDNA, mRNA sequence		
EL750087_3 AD0004002 Taenia solium UNAM-cd1_adult Taenia solium cDNA, mRNA sequence		
EL750049_4 AD0003191 Taenia solium UNAM-cd1_adult Taenia solium cDNA, mRNA sequence		
EL750037_4 AD0003179 Taenia solium UNAM-cd1_adult Taenia solium cDNA, mRNA sequence		
EL750025_4 AD0003167 Taenia solium UNAM-cd1_adult Taenia solium cDNA, mRNA sequence		
EL750021_4 AD0003163 Taenia solium UNAM-cd1_adult Taenia solium cDNA, mRNA sequence		
EL750015_5 AD0003157 Taenia solium UNAM-cd1_adult Taenia solium cDNA, mRNA sequence		
EL749978_4 AD0003120 Taenia solium UNAM-cd1_adult Taenia solium cDNA, mRNA sequence		
EL749954_6 AD0003096 Taenia solium UNAM-cd1_adult Taenia solium cDNA, mRNA sequence		
EL749933_6 AD0003075 Taenia solium UNAM-cd1_adult Taenia solium cDNA, mRNA sequence		
EL749889_4 AD0003031 Taenia solium UNAM-cd1_adult Taenia solium cDNA, mRNA sequence		
EL749884_4 AD0003026 Taenia solium UNAM-cd1_adult Taenia solium cDNA, mRNA sequence		
EL749882_6 AD0003024 Taenia solium UNAM-cd1_adult Taenia solium cDNA, mRNA sequence		
EL749829_2 AD0002193 Taenia solium UNAM-cd1_adult Taenia solium cDNA, mRNA sequence		
EL749799_2 AD0002163 Taenia solium UNAM-cd1_adult Taenia solium cDNA, mRNA sequence		
EL749795_1 AD0002159 Taenia solium UNAM-cd1_adult Taenia solium cDNA, mRNA sequence		
EL749790_3 AD0002154 Taenia solium UNAM-cd1_adult Taenia solium cDNA, mRNA sequence		
EL749755_3 AD0002119 Taenia solium UNAM-cd1_adult Taenia solium cDNA, mRNA sequence		
EL749733_3 AD0002097 Taenia solium UNAM-cd1_adult Taenia solium cDNA, mRNA sequence		
EL749710_3 AD0002074 Taenia solium UNAM-cd1_adult Taenia solium cDNA, mRNA sequence		
EL749665_2 AD0002029 Taenia solium UNAM-cd1_adult Taenia solium cDNA, mRNA sequence		
EL749662_2 AD0002026 Taenia solium UNAM-cd1_adult Taenia solium cDNA, mRNA sequence		
EL749622_3 AD0001245 Taenia solium UNAM-cd1_adult Taenia solium cDNA, mRNA sequence		
EL749621_1 AD0001244 Taenia solium UNAM-cd1_adult Taenia solium cDNA, mRNA sequence		
EL749613_2 AD0001236 Taenia solium UNAM-cd1_adult Taenia solium cDNA, mRNA sequence		
EL749590_2 AD0001213 Taenia solium UNAM-cd1_adult Taenia solium cDNA, mRNA sequence		
EL749589_2 AD0001212 Taenia solium UNAM-cd1_adult Taenia solium cDNA, mRNA sequence		
EL749583_3 AD0001206 Taenia solium UNAM-cd1_adult Taenia solium cDNA, mRNA sequence		
EL749579_2 AD0001202 Taenia solium UNAM-cd1_adult Taenia solium cDNA, mRNA sequence		
EL749569_3 AD0001192 Taenia solium UNAM-cd1_adult Taenia solium cDNA, mRNA sequence		
EL749560_2 AD0001183 Taenia solium UNAM-cd1_adult Taenia solium cDNA, mRNA sequence		
EL749545_3 AD0001168 Taenia solium UNAM-cd1_adult Taenia solium cDNA, mRNA sequence		
EL749461_2 AD0001084 Taenia solium UNAM-cd1_adult Taenia solium cDNA, mRNA sequence		
EL749439_2 AD0001062 Taenia solium UNAM-cd1_adult Taenia solium cDNA, mRNA sequence		
EL749405_2 AD0001028 Taenia solium UNAM-cd1_adult Taenia solium cDNA, mRNA sequence		
EL749404_2 AD0001027 Taenia solium UNAM-cd1_adult Taenia solium cDNA, mRNA sequence		
EL749402_2 AD0001025 Taenia solium UNAM-cd1_adult Taenia solium cDNA, mRNA sequence		

Table 1: Continued.

**Protein representing protein group**<br>>Protein identified by ESTs<br>EST	number of ESTs linked to protein	TBLASTN score
EL748354_1 LV0338043 Taenia solium UNAM-cd2_larva Taenia solium cDNA, mRNA sequence		
EL747809_2 LV0329047 Taenia solium UNAM-cd2_larva Taenia solium cDNA, mRNA sequence		
EL747744_3 LV0328032 Taenia solium UNAM-cd2_larva Taenia solium cDNA, mRNA sequence		
EL747641_1 LV0326057 Taenia solium UNAM-cd2_larva Taenia solium cDNA, mRNA sequence		
EL747510_2 LV0324046 Taenia solium UNAM-cd2_larva Taenia solium cDNA, mRNA sequence		
EL747167_3 LV0319026 Taenia solium UNAM-cd2_larva Taenia solium cDNA, mRNA sequence		
EL747004_2 LV0317001 Taenia solium UNAM-cd2_larva Taenia solium cDNA, mRNA sequence		
EL746416_6 LV0308066 Taenia solium UNAM-cd2_larva Taenia solium cDNA, mRNA sequence		
EL745403_3 LV0290058 Taenia solium UNAM-cd2_larva Taenia solium cDNA, mRNA sequence		
EL745316_1 LV0289028 Taenia solium UNAM-cd2_larva Taenia solium cDNA, mRNA sequence		
EL744541_3 LV0275016 Taenia solium UNAM-cd2_larva Taenia solium cDNA, mRNA sequence		
EL744298_2 LV0270045 Taenia solium UNAM-cd2_larva Taenia solium cDNA, mRNA sequence		
EL742621_3 LV0244052 Taenia solium UNAM-cd2_larva Taenia solium cDNA, mRNA sequence		
EL740526_1 LV0211003 Taenia solium UNAM-cd2_larva Taenia solium cDNA, mRNA sequence		
**Translation initiation factor 5A**		
>gi|6752917|gb|AAF27938.1|AF225297_1 translation initiation factor 5A [Euphorbia esula]	found 1 time	339
EL750632_3 AD0006071 Taenia solium UNAM-cd1_adult Taenia solium cDNA, mRNA sequence		
>gi|384494827|gb|EIE85318.1| eukaryotic translation initiation factor 5A [Rhizopus delemar RA 99-880]	found 3 times	328
EL763408_2 AD0203053 Taenia solium UNAM-cd1_adult Taenia solium cDNA, mRNA sequence		
EL754012_2 AD0020057 Taenia solium UNAM-cd1_adult Taenia solium cDNA, mRNA sequence		
EL747485_3 LV0324021 Taenia solium UNAM-cd2_larva Taenia solium cDNA, mRNA sequence		
**Phosphoglycerate mutase**		
>gi|60594751|gb|AAX29976.1| phosphoglycerate mutase [Clonorchis sinensis]	found 1 time	318
EL748405_1 LV0339043 Taenia solium UNAM-cd2_larva Taenia solium cDNA, mRNA sequence		
>gi|167541050|gb|ABZ82035.1| phosphoglycerate mutase [Clonorchis sinensis]	found 5 times	318
EL758632_1 AD0109007 Taenia solium UNAM-cd1_adult Taenia solium cDNA, mRNA sequence		
EL757636_1 AD0035229 Taenia solium UNAM-cd1_adult Taenia solium cDNA, mRNA sequence		
EL752270_6 AD0013037 Taenia solium UNAM-cd1_adult Taenia solium cDNA, mRNA sequence		
EL752016_3 AD0012044 Taenia solium UNAM-cd1_adult Taenia solium cDNA, mRNA sequence		
EL749216_3 LV0355030 Taenia solium UNAM-cd2_larva Taenia solium cDNA, mRNA sequence		
>gi|256088898|ref|XP_002580559.1| phosphoglycerate mutase [Schistosoma mansoni]	found 1 time	304
GT893698_2 TSAP.R46.esd Taenia solium cysticerca cDNA library Taenia solium cDNA, mRNA sequence		
**Aspartate aminotransferase**		
>gi|91084123|ref|XP_967224.1| PREDICTED: similar to aspartate aminotransferase [Tribolium castaneum]	found 3 times	731
EL759714_2 AD0128003 Taenia solium UNAM-cd1_adult Taenia solium cDNA, mRNA sequence		
EL753611_2 AD0018149 Taenia solium UNAM-cd1_adult Taenia solium cDNA, mRNA sequence		
EL742110_1 LV0236039 Taenia solium UNAM-cd2_larva Taenia solium cDNA, mRNA sequence		
>gi|332267417|ref|XP_003282681.1| PREDICTED: aspartate aminotransferase, mitochondrial, partial [Nomascus leucogenys]	found 1 time	743
EL759324_2 AD0121020 Taenia solium UNAM-cd1_adult Taenia solium cDNA, mRNA sequence		
>gi|226466868|emb|CAX69569.1| glutamate oxaloacetate transaminase 2 [Schistosoma japonicum]	found 1 time	797
EL741119_1 LV0221020 Taenia solium UNAM-cd2_larva Taenia solium cDNA, mRNA sequence		
**Hypothetical protein**		
>gi|21726976|emb|CAD38263.1| hypothetical protein [Taenia solium]	found 2 times	286
EL745461_3 LV0291053 Taenia solium UNAM-cd2_larva Taenia solium cDNA, mRNA sequence		
EL743846_1 LV0263015 Taenia solium UNAM-cd2_larva Taenia solium cDNA, mRNA sequence		
**Calcium binding protein 39**		
>gi|56757336|gb|AAW26839.1| SJCHGC00677 protein [Schistosoma japonicum]	found 1 time	305
EL757675_1 AD0094015 Taenia solium UNAM-cd1_adult Taenia solium cDNA, mRNA sequence		
>gi|358338553|dbj|GAA56968.1| calcium binding protein 39 [Clonorchis sinensis]	found 1 time	317
EL749362_2 LV0359040 Taenia solium UNAM-cd2_larva Taenia solium cDNA, mRNA sequence		
**Diagnostic antigen GP50**		
>gi|37787347|gb|AAP49287.1| diagnostic antigen GP50b precursor [Taenia solium]	found 8 times	188
EL761999_5 AD0176005 Taenia solium UNAM-cd1_adult Taenia solium cDNA, mRNA sequence		
EL753711_1 AD0018249 Taenia solium UNAM-cd1_adult Taenia solium cDNA, mRNA sequence		
EL746442_1 LV0309012 Taenia solium UNAM-cd2_larva Taenia solium cDNA, mRNA sequence		
EL745557_1 LV0293035 Taenia solium UNAM-cd2_larva Taenia solium cDNA, mRNA sequence		
EL744829_3 LV0280055 Taenia solium UNAM-cd2_larva Taenia solium cDNA, mRNA sequence		
EL743677_2 LV0260065 Taenia solium UNAM-cd2_larva Taenia solium cDNA, mRNA sequence		
EL743055_2 LV0251020 Taenia solium UNAM-cd2_larva Taenia solium cDNA, mRNA sequence		
EL741584_3 LV0228066 Taenia solium UNAM-cd2_larva Taenia solium cDNA, mRNA sequence		
>gi|37787345|gb|AAP49286.1| diagnostic antigen GP50a precursor [Taenia solium]	found 3 times	188
EL745300_3 LV0289012 Taenia solium UNAM-cd2_larva Taenia solium cDNA, mRNA sequence		
EL745159_1 LV0287013 Taenia solium UNAM-cd2_larva Taenia solium cDNA, mRNA sequence		
EL745150_3 LV0287004 Taenia solium UNAM-cd2_larva Taenia solium cDNA, mRNA sequence		
**Aldose 1-epimerase**		
>gi|358334888|dbj|GAA53306.1| aldose 1-epimerase [Clonorchis sinensis]	found 2 times	457
EL757516_3 AD0035109 Taenia solium UNAM-cd1_adult Taenia solium cDNA, mRNA sequence		
EL745722_2 LV0296012 Taenia solium UNAM-cd2_larva Taenia solium cDNA, mRNA sequence		
**Lactate dehydrogenase A**		
>gi|318054471|gb|ADV35656.1| lactate dehydrogenase A [Taenia solium]	found 6 times	290
GT892121_2 TSAB.R50.esd Taenia solium cysticerca cDNA library Taenia solium cDNA, mRNA sequence		
EL763026_1 AD0197035 Taenia solium UNAM-cd1_adult Taenia solium cDNA, mRNA sequence		
EL758825_1 AD0112024 Taenia solium UNAM-cd1_adult Taenia solium cDNA, mRNA sequence		
EL742881_3 LV0248043 Taenia solium UNAM-cd2_larva Taenia solium cDNA, mRNA sequence		

Table 1: Continued.

**Protein representing protein group**<br>>Protein identified by ESTs<br>EST	number of ESTs linked to protein	TBLASTN score
EL742752_1 LV0246035 Taenia solium UNAM-cd2_larva Taenia solium cDNA, mRNA sequence		
EL741105_3 LV0221006 Taenia solium UNAM-cd2_larva Taenia solium cDNA, mRNA sequence		
3'(2'), 5'-bisphosphate nucleotidase		
>gi|91087101|ref|XP_975068.1| PREDICTED: similar to AGAP004654-PA [Tribolium castaneum]	found 1 time	256
EL757616_2 AD0035209 Taenia solium UNAM-cd1_adult Taenia solium cDNA, mRNA sequence		
>gi|226481635|emb|CAX73715.1| 3'(2'), 5'-bisphosphate nucleotidase [Schistosoma japonicum]	found 1 time	253
EL754879_2 AD0023135 Taenia solium UNAM-cd1_adult Taenia solium cDNA, mRNA sequence		
**SJCHGC02626 protein**		
>gi|29841124|gb|AAP06137.1| similar to NM_078233 ZK1073 [Schistosoma japonicum]	found 4 times	222
EL754352_2 AD0021142 Taenia solium UNAM-cd1_adult Taenia solium cDNA, mRNA sequence		
EL749315_1 LV0358026 Taenia solium UNAM-cd2_larva Taenia solium cDNA, mRNA sequence		
EL749157_1 LV0354016 Taenia solium UNAM-cd2_larva Taenia solium cDNA, mRNA sequence		
EL749109_2 LV0353021 Taenia solium UNAM-cd2_larva Taenia solium cDNA, mRNA sequence		
>gi|56754829|gb|AAW25597.1| SJCHGC02626 protein [Schistosoma japonicum]	found 2 times	222
GT893627_1 TSBE.R21.esd Taenia solium cysticerca cDNA library Taenia solium cDNA, mRNA sequence		
EL747047_3 LV0317044 Taenia solium UNAM-cd2_larva Taenia solium cDNA, mRNA sequence		
>gi|226467558|emb|CAX69655.1| hypothetical protein [Schistosoma japonicum]	found 1 time	222
EL751584_1 AD0010077 Taenia solium UNAM-cd1_adult Taenia solium cDNA, mRNA sequence		
**Lysyl oxidase-like**		
>gi|256072781|ref|XP_002572712.1| lysyl oxidase-like [Schistosoma mansoni]	found 2 times	224
GT227831_1 tscaa0_002511.z1.scf Taenia solium adult full-length cDNA library Taenia solium cDNA clone tscaa0_002511.z1.scf 5', mRNA sequence		
EL763272_2 AD0201035 Taenia solium UNAM-cd1_adult Taenia solium cDNA, mRNA sequence		
**Nucleoside diphosphate kinase B-like**		
>gi|391344390|ref|XP_003746484.1| PREDICTED: nucleoside diphosphate kinase B-like [Metaseiulus occidentalis]	found 3 times	320
EL762526_2 AD0188016 Taenia solium UNAM-cd1_adult Taenia solium cDNA, mRNA sequence		
EL752970_6 AD0016008 Taenia solium UNAM-cd1_adult Taenia solium cDNA, mRNA sequence		
EL748758_2 LV0345010 Taenia solium UNAM-cd2_larva Taenia solium cDNA, mRNA sequence		
**Leukocyte elastase inhibitor**		
>gi|417185|sp|P80229.1|ILEU_PIG RecName: Full=Leukocyte elastase inhibitor; sp|P80229|ILEU_PIG Leukocyte elastase inhibitor OS=Sus scrofa GN=SERPINB1 PE=1 SV=1	found 1 time	235
>gi|395528214|ref|XP_003766226.1| PREDICTED: neuroserpin [Sarcophilus harrisii]	found 1 time	200
EL740855_2 LV0217038 Taenia solium UNAM-cd2_larva Taenia solium cDNA, mRNA sequence		
**SJCHGC05968 protein**		
>gi|268578813|ref|XP_002644389.1| C. briggsae CBR-UVT-5 protein [Caenorhabditis briggsae]	found 1 time	258
GT890735_1 TSAV.R40.esd Taenia solium cysticerca cDNA library Taenia solium cDNA, mRNA sequence		
>gi|76154347|gb|AAX25834.2| SJCHGC05968 protein [Schistosoma japonicum]	found 1 time	269
GT227533_3 tscaa0_001553.z1.scf Taenia solium adult full-length cDNA library Taenia solium cDNA clone tscaa0_001553.z1.scf 5', mRNA sequence		
**Ubiquitin-conjugating enzyme**		
>gi|91092198|ref|XP_969360.1| PREDICTED: similar to ubiquitin conjugating enzyme E2 [Tribolium castaneum]	found 1 time	265
EL754468_3 AD0021258 Taenia solium UNAM-cd1_adult Taenia solium cDNA, mRNA sequence		
>gi|332030895|gb|EGI70531.1| Ubiquitin-conjugating enzyme E2 N [Acromyrmex echinatior]	found 5 times	262
EL757546_1 AD0035139 Taenia solium UNAM-cd1_adult Taenia solium cDNA, mRNA sequence		
EL756485_2 AD0030017 Taenia solium UNAM-cd1_adult Taenia solium cDNA, mRNA sequence		
EL755494_3 AD0026017 Taenia solium UNAM-cd1_adult Taenia solium cDNA, mRNA sequence		
EL755239_2 AD0025009 Taenia solium UNAM-cd1_adult Taenia solium cDNA, mRNA sequence		
EL743194_2 LV0253041 Taenia solium UNAM-cd2_larva Taenia solium cDNA, mRNA sequence		
>gi|226470036|emb|CAX70299.1| ubiquitin-conjugating enzyme E2G 2 [Schistosoma japonicum]	found 3 times	445
GT891100_2 TSAV.R48.esd Taenia solium cysticerca cDNA library Taenia solium cDNA, mRNA sequence		
GT893296_2 TSBY.R51.esd Taenia solium cysticerca cDNA library Taenia solium cDNA, mRNA sequence		
EL743128_2 LV0252029 Taenia solium UNAM-cd2_larva Taenia solium cDNA, mRNA sequence		
>gi|332374456|gb|AEE62369.1| unknown [Dendroctonus ponderosae]	found 1 time	270
EL745086_2 LV0286002 Taenia solium UNAM-cd2_larva Taenia solium cDNA, mRNA sequence		
**Methylthioadenosine phosphorylase**		
>gi|346466419|gb|AEO33054.1| hypothetical protein [Amblyomma maculatum]	found 1 time	314
GT227185_2 tscaa0_000192.z1.scf Taenia solium adult full-length cDNA library Taenia solium cDNA clone tscaa0_000192.z1.scf 5', mRNA sequence		
>gi|360043716|emb|CCD81262.1| putative methylthioadenosine phosphorylase [Schistosoma mansoni]	found 1 time	317
EL740533_4 LV0211010 Taenia solium UNAM-cd2_larva Taenia solium cDNA, mRNA sequence		
**Fructose-bisphosphate aldolase**		
>gi|29336561|sp|Q9GP32.1|ALF_ECHMU RecName: Full=Fructose-bisphosphate aldolase	found 9 times	916
EL763235_3 AD0200050 Taenia solium UNAM-cd1_adult Taenia solium cDNA, mRNA sequence		
EL762777_1 AD0193024 Taenia solium UNAM-cd1_adult Taenia solium cDNA, mRNA sequence		
EL756022_2 AD0028080 Taenia solium UNAM-cd1_adult Taenia solium cDNA, mRNA sequence		
EL753514_2 AD0018052 Taenia solium UNAM-cd1_adult Taenia solium cDNA, mRNA sequence		
EL752192_1 AD0012220 Taenia solium UNAM-cd1_adult Taenia solium cDNA, mRNA sequence		
EL746034_1 LV0300065 Taenia solium UNAM-cd2_larva Taenia solium cDNA, mRNA sequence		
EL745133_1 LV0286050 Taenia solium UNAM-cd2_larva Taenia solium cDNA, mRNA sequence		
EL743540_1 LV0259003 Taenia solium UNAM-cd2_larva Taenia solium cDNA, mRNA sequence		
EL741540_2 LV0228022 Taenia solium UNAM-cd2_larva Taenia solium cDNA, mRNA sequence		
**Dynein light chain**		

Table 1: Continued.

<table>
<tr><th>Protein representing protein group
>Protein identified by ESTs
EST</th><th>number of ESTs linked to protein</th><th>TBLASTN score</th></tr>
<tr><td>>gi|72151106|ref|XP_797363.1| PREDICTED: dynein light chain LC6, flagellar outer arm-like [Strongylocentrotus purpuratus]</td><td>found 5 times</td><td>291</td></tr>
<tr><td>EL747991_3 LV0332013 Taenia solium UNAM-cd2_larva Taenia solium cDNA, mRNA sequence</td><td></td><td></td></tr>
<tr><td>EL746707_3 LV0312068 Taenia solium UNAM-cd2_larva Taenia solium cDNA, mRNA sequence</td><td></td><td></td></tr>
<tr><td>EL744208_1 LV0269019 Taenia solium UNAM-cd2_larva Taenia solium cDNA, mRNA sequence</td><td></td><td></td></tr>
<tr><td>EL744163_3 LV0268030 Taenia solium UNAM-cd2_larva Taenia solium cDNA, mRNA sequence</td><td></td><td></td></tr>
<tr><td>EL741694_1 LV0230041 Taenia solium UNAM-cd2_larva Taenia solium cDNA, mRNA sequence</td><td></td><td></td></tr>
<tr><td>>gi|119614889|gb|EAW94483.1| dynein, light chain, LC8-type 2, isoform CRA_a [Homo sapiens]</td><td>found 3 times</td><td>287</td></tr>
<tr><td>EL745515_2 LV0292049 Taenia solium UNAM-cd2_larva Taenia solium cDNA, mRNA sequence</td><td></td><td></td></tr>
<tr><td>EL744233_2 LV0269044 Taenia solium UNAM-cd2_larva Taenia solium cDNA, mRNA sequence</td><td></td><td></td></tr>
<tr><td>EL743768_3 LV0262009 Taenia solium UNAM-cd2_larva Taenia solium cDNA, mRNA sequence</td><td></td><td></td></tr>
<tr><td>>gi|159162926|pdb|1RE6|A Chain A, Localisation Of Dynein Light Chains 1 And 2 And Their Pro- Apoptotic Ligands</td><td>found 1 time</td><td>289</td></tr>
<tr><td>EL747743_1 LV0328031 Taenia solium UNAM-cd2_larva Taenia solium cDNA, mRNA sequence</td><td></td><td></td></tr>
<tr><td>>gi|112180611|gb|AAH56312.2| Dnl2 protein, partial [Danio rerio]</td><td>found 2 times</td><td>284</td></tr>
<tr><td>EL746667_2 LV0312028 Taenia solium UNAM-cd2_larva Taenia solium cDNA, mRNA sequence</td><td></td><td></td></tr>
<tr><td>EL744809_1 LV0280035 Taenia solium UNAM-cd2_larva Taenia solium cDNA, mRNA sequence</td><td></td><td></td></tr>
<tr><td>>gi|346470215|gb|AEO34952.1| hypothetical protein [Amblyomma maculatum]</td><td>found 2 times</td><td>299</td></tr>
<tr><td>EL758264_6 AD0103008 Taenia solium UNAM-cd1_adult Taenia solium cDNA, mRNA sequence</td><td></td><td></td></tr>
<tr><td>EL748083_1 LV0333034 Taenia solium UNAM-cd2_larva Taenia solium cDNA, mRNA sequence</td><td></td><td></td></tr>
<tr><td>>gi|403279715|ref|XP_003931391.1| PREDICTED: dynein light chain 2, cytoplasmic [Saimiri boliviensis boliviensis]</td><td>found 17 times</td><td>298</td></tr>
<tr><td>EL748177_1 LV0334060 Taenia solium UNAM-cd2_larva Taenia solium cDNA, mRNA sequence</td><td></td><td></td></tr>
<tr><td>EL746880_3 LV0315022 Taenia solium UNAM-cd2_larva Taenia solium cDNA, mRNA sequence</td><td></td><td></td></tr>
<tr><td>EL746825_2 LV0314038 Taenia solium UNAM-cd2_larva Taenia solium cDNA, mRNA sequence</td><td></td><td></td></tr>
<tr><td>EL746779_3 LV0313062 Taenia solium UNAM-cd2_larva Taenia solium cDNA, mRNA sequence</td><td></td><td></td></tr>
<tr><td>EL746591_1 LV0311020 Taenia solium UNAM-cd2_larva Taenia solium cDNA, mRNA sequence</td><td></td><td></td></tr>
<tr><td>EL746481_3 LV0309051 Taenia solium UNAM-cd2_larva Taenia solium cDNA, mRNA sequence</td><td></td><td></td></tr>
<tr><td>EL743939_1 LV0265011 Taenia solium UNAM-cd2_larva Taenia solium cDNA, mRNA sequence</td><td></td><td></td></tr>
<tr><td>EL743885_1 LV0264014 Taenia solium UNAM-cd2_larva Taenia solium cDNA, mRNA sequence</td><td></td><td></td></tr>
<tr><td>EL743554_2 LV0259017 Taenia solium UNAM-cd2_larva Taenia solium cDNA, mRNA sequence</td><td></td><td></td></tr>
<tr><td>EL743121_1 LV0252022 Taenia solium UNAM-cd2_larva Taenia solium cDNA, mRNA sequence</td><td></td><td></td></tr>
<tr><td>EL742969_2 LV0250010 Taenia solium UNAM-cd2_larva Taenia solium cDNA, mRNA sequence</td><td></td><td></td></tr>
<tr><td>EL742591_1 LV0244022 Taenia solium UNAM-cd2_larva Taenia solium cDNA, mRNA sequence</td><td></td><td></td></tr>
<tr><td>EL742357_3 LV0240052 Taenia solium UNAM-cd2_larva Taenia solium cDNA, mRNA sequence</td><td></td><td></td></tr>
<tr><td>EL742122_2 LV0237010 Taenia solium UNAM-cd2_larva Taenia solium cDNA, mRNA sequence</td><td></td><td></td></tr>
<tr><td>EL741998_3 LV0234059 Taenia solium UNAM-cd2_larva Taenia solium cDNA, mRNA sequence</td><td></td><td></td></tr>
<tr><td>EL741357_3 LV0225008 Taenia solium UNAM-cd2_larva Taenia solium cDNA, mRNA sequence</td><td></td><td></td></tr>
<tr><td>EL740256_1 LV0205036 Taenia solium UNAM-cd2_larva Taenia solium cDNA, mRNA sequence</td><td></td><td></td></tr>
<tr><td>>gi|145505966|ref|XP_001438949.1| hypothetical protein [Paramecium tetraurelia strain d4-2]</td><td>found 4 times</td><td>283</td></tr>
<tr><td>EL746949_3 LV0316026 Taenia solium UNAM-cd2_larva Taenia solium cDNA, mRNA sequence</td><td></td><td></td></tr>
<tr><td>EL746095_2 LV0301050 Taenia solium UNAM-cd2_larva Taenia solium cDNA, mRNA sequence</td><td></td><td></td></tr>
<tr><td>EL744369_1 LV0271051 Taenia solium UNAM-cd2_larva Taenia solium cDNA, mRNA sequence</td><td></td><td></td></tr>
<tr><td>EL741925_3 LV0233058 Taenia solium UNAM-cd2_larva Taenia solium cDNA, mRNA sequence</td><td></td><td></td></tr>
<tr><td>>gi|291001365|ref|XP_002683249.1| predicted protein [Naegleria gruberi]</td><td>found 1 time</td><td>268</td></tr>
<tr><td>EL762870_2 AD0194041 Taenia solium UNAM-cd1_adult Taenia solium cDNA, mRNA sequence</td><td></td><td></td></tr>
<tr><td>>gi|124784009|gb|ABN14960.1| calcium-binding protein [Taenia asiatica]</td><td>found 7 times</td><td>562</td></tr>
<tr><td>EL761739_6 AD0170010 Taenia solium UNAM-cd1_adult Taenia solium cDNA, mRNA sequence</td><td></td><td></td></tr>
<tr><td>EL761630_6 AD0167020 Taenia solium UNAM-cd1_adult Taenia solium cDNA, mRNA sequence</td><td></td><td></td></tr>
<tr><td>EL754281_1 AD0021071 Taenia solium UNAM-cd1_adult Taenia solium cDNA, mRNA sequence</td><td></td><td></td></tr>
<tr><td>EL747257_3 LV0320054 Taenia solium UNAM-cd2_larva Taenia solium cDNA, mRNA sequence</td><td></td><td></td></tr>
<tr><td>EL746252_2 LV0306003 Taenia solium UNAM-cd2_larva Taenia solium cDNA, mRNA sequence</td><td></td><td></td></tr>
<tr><td>EL744759_2 LV0279028 Taenia solium UNAM-cd2_larva Taenia solium cDNA, mRNA sequence</td><td></td><td></td></tr>
<tr><td>EL744483_2 LV0273041 Taenia solium UNAM-cd2_larva Taenia solium cDNA, mRNA sequence</td><td></td><td></td></tr>
<tr><td>Tegumental protein</td><td></td><td></td></tr>
<tr><td>>gi|60459970|gb|AAX20156.1| tegumental protein [Echinococcus granulosus]</td><td>found 37 times</td><td>538</td></tr>
<tr><td>EL761848_4 AD0172015 Taenia solium UNAM-cd1_adult Taenia solium cDNA, mRNA sequence</td><td></td><td></td></tr>
<tr><td>EL761051_4 AD0155039 Taenia solium UNAM-cd1_adult Taenia solium cDNA, mRNA sequence</td><td></td><td></td></tr>
<tr><td>EL759340_2 AD0121036 Taenia solium UNAM-cd1_adult Taenia solium cDNA, mRNA sequence</td><td></td><td></td></tr>
<tr><td>EL758769_3 AD0111034 Taenia solium UNAM-cd1_adult Taenia solium cDNA, mRNA sequence</td><td></td><td></td></tr>
<tr><td>EL757429_2 AD0035022 Taenia solium UNAM-cd1_adult Taenia solium cDNA, mRNA sequence</td><td></td><td></td></tr>
<tr><td>EL757202_1 AD0034018 Taenia solium UNAM-cd1_adult Taenia solium cDNA, mRNA sequence</td><td></td><td></td></tr>
<tr><td>EL756120_2 AD0028178 Taenia solium UNAM-cd1_adult Taenia solium cDNA, mRNA sequence</td><td></td><td></td></tr>
<tr><td>EL755670_3 AD0026193 Taenia solium UNAM-cd1_adult Taenia solium cDNA, mRNA sequence</td><td></td><td></td></tr>
<tr><td>EL755314_2 AD0025084 Taenia solium UNAM-cd1_adult Taenia solium cDNA, mRNA sequence</td><td></td><td></td></tr>
<tr><td>EL755020_1 AD0024037 Taenia solium UNAM-cd1_adult Taenia solium cDNA, mRNA sequence</td><td></td><td></td></tr>
<tr><td>EL754938_1 AD0023194 Taenia solium UNAM-cd1_adult Taenia solium cDNA, mRNA sequence</td><td></td><td></td></tr>
<tr><td>EL754722_2 AD0022247 Taenia solium UNAM-cd1_adult Taenia solium cDNA, mRNA sequence</td><td></td><td></td></tr>
<tr><td>EL752851_6 AD0015110 Taenia solium UNAM-cd1_adult Taenia solium cDNA, mRNA sequence</td><td></td><td></td></tr>
<tr><td>EL752595_1 AD0014136 Taenia solium UNAM-cd1_adult Taenia solium cDNA, mRNA sequence</td><td></td><td></td></tr>
<tr><td>EL752553_2 AD0014094 Taenia solium UNAM-cd1_adult Taenia solium cDNA, mRNA sequence</td><td></td><td></td></tr>
<tr><td>EL751828_6 AD0011071 Taenia solium UNAM-cd1_adult Taenia solium cDNA, mRNA sequence</td><td></td><td></td></tr>
<tr><td>EL751801_6 AD0011044 Taenia solium UNAM-cd1_adult Taenia solium cDNA, mRNA sequence</td><td></td><td></td></tr>
<tr><td>EL751591_3 AD0010084 Taenia solium UNAM-cd1_adult Taenia solium cDNA, mRNA sequence</td><td></td><td></td></tr>
<tr><td>EL751561_1 AD0010054 Taenia solium UNAM-cd1_adult Taenia solium cDNA, mRNA sequence</td><td></td><td></td></tr>
<tr><td>EL751467_5 AD0009205 Taenia solium UNAM-cd1_adult Taenia solium cDNA, mRNA sequence</td><td></td><td></td></tr>
<tr><td>EL751389_5 AD0009127 Taenia solium UNAM-cd1_adult Taenia solium cDNA, mRNA sequence</td><td></td><td></td></tr>
<tr><td>EL751283_5 AD0009021 Taenia solium UNAM-cd1_adult Taenia solium cDNA, mRNA sequence</td><td></td><td></td></tr>
<tr><td>EL751219_1 AD0008230 Taenia solium UNAM-cd1_adult Taenia solium cDNA, mRNA sequence</td><td></td><td></td></tr>
<tr><td>EL751136_1 AD0008147 Taenia solium UNAM-cd1_adult Taenia solium cDNA, mRNA sequence</td><td></td><td></td></tr>
<tr><td>EL751015_1 AD0008026 Taenia solium UNAM-cd1_adult Taenia solium cDNA, mRNA sequence</td><td></td><td></td></tr>
<tr><td>EL750853_6 AD0007089 Taenia solium UNAM-cd1_adult Taenia solium cDNA, mRNA sequence</td><td></td><td></td></tr>
<tr><td>EL750488_6 AD0005151 Taenia solium UNAM-cd1_adult Taenia solium cDNA, mRNA sequence</td><td></td><td></td></tr>
<tr><td>EL750360_5 AD0005023 Taenia solium UNAM-cd1_adult Taenia solium cDNA, mRNA sequence</td><td></td><td></td></tr>
</table>

Table 1: Continued.

**Protein representing protein group**<br>>Protein identified by ESTs<br>EST	number of ESTs linked to protein	TBLASTN score
EL750250_2 AD0004165 Taenia solium UNAM-cd1_adult Taenia solium cDNA, mRNA sequence		
EL750108_1 AD0004023 Taenia solium UNAM-cd1_adult Taenia solium cDNA, mRNA sequence		
EL749606_1 AD0001229 Taenia solium UNAM-cd1_adult Taenia solium cDNA, mRNA sequence		
EL749553_3 AD0001176 Taenia solium UNAM-cd1_adult Taenia solium cDNA, mRNA sequence		
EL745905_1 LV0298062 Taenia solium UNAM-cd2_larva Taenia solium cDNA, mRNA sequence		
EL744223_1 LV0269034 Taenia solium UNAM-cd2_larva Taenia solium cDNA, mRNA sequence		
EL742979_1 LV0250020 Taenia solium UNAM-cd2_larva Taenia solium cDNA, mRNA sequence		
EL742139_1 LV0237027 Taenia solium UNAM-cd2_larva Taenia solium cDNA, mRNA sequence		
EL740252_3 LV0205032 Taenia solium UNAM-cd2_larva Taenia solium cDNA, mRNA sequence		
**Methionine sulfoxide reductase**		
>gi|130492918|ref|NP_001076362.1| peptide methionine sulfoxide reductase [Danio rerio]	found 1 time	329
EL740517_3 LV0210041 Taenia solium UNAM-cd2_larva Taenia solium cDNA, mRNA sequence		
>gi|338999577|ref|ZP_08638219.1| methionine sulfoxide reductase A [Halomonas sp. TD01]	found 3 times	343
EL758473_3 AD0106016 Taenia solium UNAM-cd1_adult Taenia solium cDNA, mRNA sequence		
EL756599_1 AD0030131 Taenia solium UNAM-cd1_adult Taenia solium cDNA, mRNA sequence		
EL755598_1 AD0026121 Taenia solium UNAM-cd1_adult Taenia solium cDNA, mRNA sequence		
**Glycogen phosphorylase muscle form**		
>gi|76156528|gb|AAX27719.2| SJCHGC00991 protein [Schistosoma japonicum]	found 3 times	389
EL750163_3 AD0004078 Taenia solium UNAM-cd1_adult Taenia solium cDNA, mRNA sequence		
EL747076_3 LV0318008 Taenia solium UNAM-cd2_larva Taenia solium cDNA, mRNA sequence		
EL741014_2 LV0219064 Taenia solium UNAM-cd2_larva Taenia solium cDNA, mRNA sequence		
>gi|358337175|dbj|GAA55581.1| glycogen phosphorylase muscle form [Clonorchis sinensis]	found 4 times	220
GT889492_1 TSBU.R60.esd Taenia solium cysticerca cDNA library Taenia solium cDNA, mRNA sequence		
GT892076_3 TSBO.R39.esd Taenia solium cysticerca cDNA library Taenia solium cDNA, mRNA sequence		
GT889847_3 TSBQ.R94.esd Taenia solium cysticerca cDNA library Taenia solium cDNA, mRNA sequence		
EL740644_2 LV0213026 Taenia solium UNAM-cd2_larva Taenia solium cDNA, mRNA sequence		
**Ornithine aminotransferase**		
>gi|158297847|ref|XP_001689079.1| AGAP004793-PB [Anopheles gambiae str. PEST]	found 1 time	559
GT227016_2 tscaa0_002505.z1.scf Taenia solium adult full-length cDNA library Taenia solium cDNA clone tscaa0_002505.z1.scf 5', mRNA sequence		
>gi|317419517|emb|CBN81554.1| Ornithine aminotransferase, mitochondrial [Dicentrarchus labrax]	found 1 time	579
EL759925_2 AD0132016 Taenia solium UNAM-cd1_adult Taenia solium cDNA, mRNA sequence		
>gi|226471034|emb|CAX70598.1| ornithine aminotransferase [Schistosoma japonicum]	found 1 time	590
EL748622_1 LV0343007 Taenia solium UNAM-cd2_larva Taenia solium cDNA, mRNA sequence		
**Endoglycoceramidase**		
>gi|260788730|ref|XP_002589402.1| hypothetical protein BRAFLDRAFT_77844 [Branchiostoma floridae]	found 1 time	259
EL744169_2 LV0268036 Taenia solium UNAM-cd2_larva Taenia solium cDNA, mRNA sequence		
>gi|350644390|emb|CCD60877.1| hypothetical protein Smp_187410 [Schistosoma mansoni]	found 1 time	227
EL746757_3 LV0313040 Taenia solium UNAM-cd2_larva Taenia solium cDNA, mRNA sequence		
>gi|358337987|dbj|GAA34745.2| endoglycoceramidase [Clonorchis sinensis]	found 1 time	538
EL744170_2 LV0268037 Taenia solium UNAM-cd2_larva Taenia solium cDNA, mRNA sequence		
**Carbonic anhydrase**		
>gi|56711366|ref|NP_001008688.1| carbonic anhydrase 3 [Sus scrofa]	found 1 time	210
sp|Q5S1S4|CAH3_PIG Carbonic anhydrase 3 OS=Sus scrofa GN=CA3 PE=2 SV=3		
>gi|256076010|ref|XP_002574308.1| carbonic anhydrase II (carbonate dehydratase II) [Schistosoma mansoni]	found 2 times	209
EL762410_4 AD0185015 Taenia solium UNAM-cd1_adult Taenia solium cDNA, mRNA sequence		
EL761032_6 AD0155020 Taenia solium UNAM-cd1_adult Taenia solium cDNA, mRNA sequence		
>gi|256076012|ref|XP_002574309.1| carbonic anhydrase II (carbonate dehydratase II) [Schistosoma mansoni]	found 2 times	209
EL758749_1 AD0111014 Taenia solium UNAM-cd1_adult Taenia solium cDNA, mRNA sequence		
EL755200_3 AD0024217 Taenia solium UNAM-cd1_adult Taenia solium cDNA, mRNA sequence		
**Dipeptidyl-peptidase**		
>gi|256074069|ref|XP_002573349.1| dipeptidyl-peptidase III (M49 family) [Schistosoma mansoni]	found 1 time	1043
EL740267_2 LV0205047 Taenia solium UNAM-cd2_larva Taenia solium cDNA, mRNA sequence		
>gi|358339394|dbj|GAA47464.1| dipeptidyl-peptidase III [Clonorchis sinensis]	found 2 times	1065
EL748899_3 LV0348010 Taenia solium UNAM-cd2_larva Taenia solium cDNA, mRNA sequence		
EL742343_1 LV0240038 Taenia solium UNAM-cd2_larva Taenia solium cDNA, mRNA sequence		
**Transketolase**		
>gi|27526313|emb|CAD45181.1| transketolase [Echinococcus multilocularis]	found 5 times	581
GT227009_3 tscaa0_000809.z1.scf Taenia solium adult full-length cDNA library Taenia solium cDNA clone tscaa0_000809.z1.scf 5', mRNA sequence		
EL763321_1 AD0202021 Taenia solium UNAM-cd1_adult Taenia solium cDNA, mRNA sequence		
EL761843_6 AD0172010 Taenia solium UNAM-cd1_adult Taenia solium cDNA, mRNA sequence		
EL759084_3 AD0116047 Taenia solium UNAM-cd1_adult Taenia solium cDNA, mRNA sequence		
EL749337_1 LV0359015 Taenia solium UNAM-cd2_larva Taenia solium cDNA, mRNA sequence		
**Porphobilinogen synthase**		
>gi|157127939|ref|XP_001661237.1| porphobilinogen synthase [Aedes aegypti]	found 1 time	234
EL749705_2 AD0002069 Taenia solium UNAM-cd1_adult Taenia solium cDNA, mRNA sequence		
>gi|358335858|dbj|GAA54460.1| porphobilinogen synthase [Clonorchis sinensis]	found 1 time	216
EL749927_6 AD0003069 Taenia solium UNAM-cd1_adult Taenia solium cDNA, mRNA sequence		
>gi|358254118|dbj|GAA54148.1| ubiquitin thioesterase protein OTUB1 [Clonorchis sinensis]	found 1 time	247
EL743389_1 LV0257006 Taenia solium UNAM-cd2_larva Taenia solium cDNA, mRNA sequence		
**procollagen-lysine, 2-oxoglutarate 5-dioxygenase 3**		
>gi|256079279|ref|XP_002575916.1| procollagen-lysine2-oxoglutarate 5-dioxygenase [Schistosoma mansoni]	found 1 time	480

Table 1: Continued.

<table>
<tr><th>Protein representing protein group
>Protein identified by ESTs
EST</th><th>number of ESTs linked to protein</th><th>TBLASTN score</th></tr>
<tr><td>EL758053_1 AD0099065 Taenia solium UNAM-cd1_adult Taenia solium cDNA, mRNA sequence</td><td></td><td></td></tr>
<tr><td>>gi|226490282|emb|CAX69383.1| procollagen-lysine, 2-oxoglutarate 5-dioxygenase 3 [Schistosoma japonicum]</td><td>found 4 times</td><td>469</td></tr>
<tr><td>EL763319_2 AD0202019 Taenia solium UNAM-cd1_adult Taenia solium cDNA, mRNA sequence</td><td></td><td></td></tr>
<tr><td>EL756640_2 AD0030172 Taenia solium UNAM-cd1_adult Taenia solium cDNA, mRNA sequence</td><td></td><td></td></tr>
<tr><td>EL754559_2 AD0022084 Taenia solium UNAM-cd1_adult Taenia solium cDNA, mRNA sequence</td><td></td><td></td></tr>
<tr><td>EL754190_2 AD0020235 Taenia solium UNAM-cd1_adult Taenia solium cDNA, mRNA sequence</td><td></td><td></td></tr>
<tr><td>>gi|358254467|dbj|GAA55391.1| lysyl hydroxylase/galactosyltransferase/ glucosyltransferase [Clonorchis sinensis]</td><td>found 1 time</td><td>439</td></tr>
<tr><td>EL758621_3 AD0108052 Taenia solium UNAM-cd1_adult Taenia solium cDNA, mRNA sequence</td><td></td><td></td></tr>
<tr><td>Actin-binding and severin family group-like protein</td><td></td><td></td></tr>
<tr><td>>gi|121118|sp|P20305.1|GELS_PIG RecName: Full=Gelsolin; AltName: Full=Actin-depolymerizing factor; sp|P20305|GELS_PIG Gelsolin (Fragment) OS=Sus scrofa GN=GSN PE=1 SV=1</td><td>found 1 time</td><td>219</td></tr>
<tr><td>>gi|26399708|sp|Q24800.3|SEVE_ECHGR RecName: Full=Severin</td><td>found 1 time</td><td>388</td></tr>
<tr><td>EL762290_6 AD0182046 Taenia solium UNAM-cd1_adult Taenia solium cDNA, mRNA sequence</td><td></td><td></td></tr>
<tr><td>>gi|12641925|gb|AAK00053.1| actin-filament fragmenting protein [Echinococcus granulosus]</td><td>found 7 times</td><td>388</td></tr>
<tr><td>GT890678_2 TSAU.R85.esd Taenia solium cysticerca cDNA library Taenia solium cDNA, mRNA sequence</td><td></td><td></td></tr>
<tr><td>EL758978_1 AD0115003 Taenia solium UNAM-cd1_adult Taenia solium cDNA, mRNA sequence</td><td></td><td></td></tr>
<tr><td>EL749094_2 LV0353006 Taenia solium UNAM-cd2_larva Taenia solium cDNA, mRNA sequence</td><td></td><td></td></tr>
<tr><td>EL746773_3 LV0313056 Taenia solium UNAM-cd2_larva Taenia solium cDNA, mRNA sequence</td><td></td><td></td></tr>
<tr><td>EL746088_2 LV0301043 Taenia solium UNAM-cd2_larva Taenia solium cDNA, mRNA sequence</td><td></td><td></td></tr>
<tr><td>EL745542_2 LV0293020 Taenia solium UNAM-cd2_larva Taenia solium cDNA, mRNA sequence</td><td></td><td></td></tr>
<tr><td>EL741057_2 LV0220030 Taenia solium UNAM-cd2_larva Taenia solium cDNA, mRNA sequence</td><td></td><td></td></tr>
<tr><td>>gi|13195551|gb|AAK15753.1| actin-binding and severin family group-like protein [Echinococcus granulosus]</td><td>found 51 times</td><td>388</td></tr>
<tr><td>EL763139_3 AD0199023 Taenia solium UNAM-cd1_adult Taenia solium cDNA, mRNA sequence</td><td></td><td></td></tr>
<tr><td>EL762236_6 AD0181033 Taenia solium UNAM-cd1_adult Taenia solium cDNA, mRNA sequence</td><td></td><td></td></tr>
<tr><td>EL762154_4 AD0179047 Taenia solium UNAM-cd1_adult Taenia solium cDNA, mRNA sequence</td><td></td><td></td></tr>
<tr><td>EL761493_4 AD0164049 Taenia solium UNAM-cd1_adult Taenia solium cDNA, mRNA sequence</td><td></td><td></td></tr>
<tr><td>EL761436_5 AD0163045 Taenia solium UNAM-cd1_adult Taenia solium cDNA, mRNA sequence</td><td></td><td></td></tr>
<tr><td>EL761012_4 AD0154044 Taenia solium UNAM-cd1_adult Taenia solium cDNA, mRNA sequence</td><td></td><td></td></tr>
<tr><td>EL759972_2 AD0133017 Taenia solium UNAM-cd1_adult Taenia solium cDNA, mRNA sequence</td><td></td><td></td></tr>
<tr><td>EL757477_3 AD0035070 Taenia solium UNAM-cd1_adult Taenia solium cDNA, mRNA sequence</td><td></td><td></td></tr>
<tr><td>EL757361_1 AD0034177 Taenia solium UNAM-cd1_adult Taenia solium cDNA, mRNA sequence</td><td></td><td></td></tr>
<tr><td>EL757342_1 AD0034158 Taenia solium UNAM-cd1_adult Taenia solium cDNA, mRNA sequence</td><td></td><td></td></tr>
<tr><td>EL757307_1 AD0034123 Taenia solium UNAM-cd1_adult Taenia solium cDNA, mRNA sequence</td><td></td><td></td></tr>
<tr><td>EL757293_1 AD0034109 Taenia solium UNAM-cd1_adult Taenia solium cDNA, mRNA sequence</td><td></td><td></td></tr>
<tr><td>EL757251_2 AD0034067 Taenia solium UNAM-cd1_adult Taenia solium cDNA, mRNA sequence</td><td></td><td></td></tr>
<tr><td>EL757212_1 AD0034028 Taenia solium UNAM-cd1_adult Taenia solium cDNA, mRNA sequence</td><td></td><td></td></tr>
<tr><td>EL756912_3 AD0031185 Taenia solium UNAM-cd1_adult Taenia solium cDNA, mRNA sequence</td><td></td><td></td></tr>
<tr><td>EL756794_1 AD0031067 Taenia solium UNAM-cd1_adult Taenia solium cDNA, mRNA sequence</td><td></td><td></td></tr>
<tr><td>EL756491_1 AD0030023 Taenia solium UNAM-cd1_adult Taenia solium cDNA, mRNA sequence</td><td></td><td></td></tr>
<tr><td>EL756103_1 AD0028161 Taenia solium UNAM-cd1_adult Taenia solium cDNA, mRNA sequence</td><td></td><td></td></tr>
<tr><td>EL756056_1 AD0028114 Taenia solium UNAM-cd1_adult Taenia solium cDNA, mRNA sequence</td><td></td><td></td></tr>
<tr><td>EL756021_3 AD0028079 Taenia solium UNAM-cd1_adult Taenia solium cDNA, mRNA sequence</td><td></td><td></td></tr>
<tr><td>EL755922_3 AD0027218 Taenia solium UNAM-cd1_adult Taenia solium cDNA, mRNA sequence</td><td></td><td></td></tr>
<tr><td>EL755906_2 AD0027202 Taenia solium UNAM-cd1_adult Taenia solium cDNA, mRNA sequence</td><td></td><td></td></tr>
<tr><td>EL755864_1 AD0027160 Taenia solium UNAM-cd1_adult Taenia solium cDNA, mRNA sequence</td><td></td><td></td></tr>
<tr><td>EL755863_1 AD0027159 Taenia solium UNAM-cd1_adult Taenia solium cDNA, mRNA sequence</td><td></td><td></td></tr>
<tr><td>EL755300_1 AD0025070 Taenia solium UNAM-cd1_adult Taenia solium cDNA, mRNA sequence</td><td></td><td></td></tr>
<tr><td>EL755207_1 AD0024224 Taenia solium UNAM-cd1_adult Taenia solium cDNA, mRNA sequence</td><td></td><td></td></tr>
<tr><td>EL755203_1 AD0024220 Taenia solium UNAM-cd1_adult Taenia solium cDNA, mRNA sequence</td><td></td><td></td></tr>
<tr><td>EL754426_1 AD0021216 Taenia solium UNAM-cd1_adult Taenia solium cDNA, mRNA sequence</td><td></td><td></td></tr>
<tr><td>EL754387_1 AD0021177 Taenia solium UNAM-cd1_adult Taenia solium cDNA, mRNA sequence</td><td></td><td></td></tr>
<tr><td>EL754329_2 AD0021119 Taenia solium UNAM-cd1_adult Taenia solium cDNA, mRNA sequence</td><td></td><td></td></tr>
<tr><td>EL754085_1 AD0020130 Taenia solium UNAM-cd1_adult Taenia solium cDNA, mRNA sequence</td><td></td><td></td></tr>
<tr><td>EL753844_1 AD0019131 Taenia solium UNAM-cd1_adult Taenia solium cDNA, mRNA sequence</td><td></td><td></td></tr>
<tr><td>EL753617_3 AD0018155 Taenia solium UNAM-cd1_adult Taenia solium cDNA, mRNA sequence</td><td></td><td></td></tr>
<tr><td>EL753524_1 AD0018062 Taenia solium UNAM-cd1_adult Taenia solium cDNA, mRNA sequence</td><td></td><td></td></tr>
<tr><td>EL752763_6 AD0015022 Taenia solium UNAM-cd1_adult Taenia solium cDNA, mRNA sequence</td><td></td><td></td></tr>
<tr><td>EL752551_1 AD0014092 Taenia solium UNAM-cd1_adult Taenia solium cDNA, mRNA sequence</td><td></td><td></td></tr>
<tr><td>EL752487_3 AD0014028 Taenia solium UNAM-cd1_adult Taenia solium cDNA, mRNA sequence</td><td></td><td></td></tr>
<tr><td>EL751473_6 AD0009211 Taenia solium UNAM-cd1_adult Taenia solium cDNA, mRNA sequence</td><td></td><td></td></tr>
<tr><td>EL751227_1 AD0008238 Taenia solium UNAM-cd1_adult Taenia solium cDNA, mRNA sequence</td><td></td><td></td></tr>
<tr><td>EL750761_1 AD0006200 Taenia solium UNAM-cd1_adult Taenia solium cDNA, mRNA sequence</td><td></td><td></td></tr>
<tr><td>EL750640_1 AD0006079 Taenia solium UNAM-cd1_adult Taenia solium cDNA, mRNA sequence</td><td></td><td></td></tr>
<tr><td>EL750521_4 AD0005184 Taenia solium UNAM-cd1_adult Taenia solium cDNA, mRNA sequence</td><td></td><td></td></tr>
<tr><td>EL750291_2 AD0004206 Taenia solium UNAM-cd1_adult Taenia solium cDNA, mRNA sequence</td><td></td><td></td></tr>
<tr><td>EL749620_1 AD0001243 Taenia solium UNAM-cd1_adult Taenia solium cDNA, mRNA sequence</td><td></td><td></td></tr>
<tr><td>EL749552_3 AD0001175 Taenia solium UNAM-cd1_adult Taenia solium cDNA, mRNA sequence</td><td></td><td></td></tr>
<tr><td>EL749494_3 AD0001117 Taenia solium UNAM-cd1_adult Taenia solium cDNA, mRNA sequence</td><td></td><td></td></tr>
<tr><td>EL749435_1 AD0001058 Taenia solium UNAM-cd1_adult Taenia solium cDNA, mRNA sequence</td><td></td><td></td></tr>
<tr><td>EL749403_1 AD0001026 Taenia solium UNAM-cd1_adult Taenia solium cDNA, mRNA sequence</td><td></td><td></td></tr>
<tr><td>EL749393_1 AD0001016 Taenia solium UNAM-cd1_adult Taenia solium cDNA, mRNA sequence</td><td></td><td></td></tr>
<tr><td>EL748514_2 LV0341030 Taenia solium UNAM-cd2_larva Taenia solium cDNA, mRNA sequence</td><td></td><td></td></tr>
<tr><td>EL746617_3 LV0311046 Taenia solium UNAM-cd2_larva Taenia solium cDNA, mRNA sequence</td><td></td><td></td></tr>
<tr><td>Hypothetical protein</td><td></td><td></td></tr>
<tr><td>>gi|256079415|ref|XP_002575983.1| hypothetical protein [Schistosoma mansoni]</td><td>found 4 times</td><td>205</td></tr>
<tr><td>GT892414_3 TSBE.R59.esd Taenia solium cysticerca cDNA library Taenia solium cDNA, mRNA sequence</td><td></td><td></td></tr>
<tr><td>GT891028_3 TSAF.R3.esd Taenia solium cysticerca cDNA library Taenia solium cDNA, mRNA sequence</td><td></td><td></td></tr>
<tr><td>EL753794_5 AD0019081 Taenia solium UNAM-cd1_adult Taenia solium cDNA, mRNA sequence</td><td></td><td></td></tr>
<tr><td>EL746946_2 LV0316023 Taenia solium UNAM-cd2_larva Taenia solium cDNA, mRNA sequence</td><td></td><td></td></tr>
</table>

Table 1: Continued.

**Protein representing protein group**<br>>Protein identified by ESTs<br>EST	number of ESTs linked to protein	TBLASTN score
**Glutamate carboxypeptidase 2**		
>gi|358331956|dbj|GAA50701.1| glutamate carboxypeptidase 2 [Clonorchis sinensis]	found 3 times	222
EL748460_1 LV0340034 Taenia solium UNAM-cd2_larva Taenia solium cDNA, mRNA sequence		
EL747676_3 LV0327019 Taenia solium UNAM-cd2_larva Taenia solium cDNA, mRNA sequence		
EL741730_1 LV0231008 Taenia solium UNAM-cd2_larva Taenia solium cDNA, mRNA sequence		
**Aminoacylase**		
>gi|348534238|ref|XP_003454610.1| PREDICTED: aminoacylase-1A-like [Oreochromis niloticus]	found 1 time	333
EL759311_1 AD0121007 Taenia solium UNAM-cd1_adult Taenia solium cDNA, mRNA sequence		
>gi|358335081|dbj|GAA30793.2| aminoacylase [Clonorchis sinensis]	found 1 time	481
EL757173_3 AD0033035 Taenia solium UNAM-cd1_adult Taenia solium cDNA, mRNA sequence		
**Filamin**		
>gi|358335748|dbj|GAA54374.1| basement membrane-specific heparan sulfate proteoglycan core protein [Clonorchis sinensis]	found 3 times	297
EL751295_4 AD0009033 Taenia solium UNAM-cd1_adult Taenia solium cDNA, mRNA sequence		
EL751031_3 AD0008042 Taenia solium UNAM-cd1_adult Taenia solium cDNA, mRNA sequence		
EL745042_2 LV0285014 Taenia solium UNAM-cd2_larva Taenia solium cDNA, mRNA sequence		
>gi|118404138|ref|NP_001072386.1| filamin A, alpha [Xenopus (Silurana) tropicalis]	found 1 time	210
EL762946_3 AD0195066 Taenia solium UNAM-cd1_adult Taenia solium cDNA, mRNA sequence		
>gi|358341099|dbj|GAA48860.1| filamin-C [Clonorchis sinensis]	found 3 times	531
EL759003_1 AD0115028 Taenia solium UNAM-cd1_adult Taenia solium cDNA, mRNA sequence		
EL752917_2 AD0015176 Taenia solium UNAM-cd1_adult Taenia solium cDNA, mRNA sequence		
EL747433_3 LV0323042 Taenia solium UNAM-cd2_larva Taenia solium cDNA, mRNA sequence		
**Glucose-6-phosphate 1-dehydrogenase-like**		
>gi|350595984|ref|XP_003360563.2| PREDICTED: glucose-6-phosphate 1-dehydrogenase-like, partial [Sus scrofa]	found 2 times	234
EL749830_5 AD0002194 Taenia solium UNAM-cd1_adult Taenia solium cDNA, mRNA sequence		
EL741305_1 LV0224025 Taenia solium UNAM-cd2_larva Taenia solium cDNA, mRNA sequence		
**Methionyl-tRNA synthetase cytoplasmic**		
>gi|358255967|dbj|GAA57560.1| methionyl-tRNA synthetase cytoplasmic [Clonorchis sinensis]	found 5 times	238
EL762957_5 AD0196010 Taenia solium UNAM-cd1_adult Taenia solium cDNA, mRNA sequence		
EL762912_2 AD0195032 Taenia solium UNAM-cd1_adult Taenia solium cDNA, mRNA sequence		
EL756751_1 AD0031024 Taenia solium UNAM-cd1_adult Taenia solium cDNA, mRNA sequence		
EL746673_3 LV0312034 Taenia solium UNAM-cd2_larva Taenia solium cDNA, mRNA sequence		
EL745111_2 LV0286028 Taenia solium UNAM-cd2_larva Taenia solium cDNA, mRNA sequence		
**Apolipoprotein AI binding protein**		
>gi|223640019|emb|CAX16804.1| apolipoprotein AI binding protein [Echinococcus multilocularis]	found 2 times	257
GT890247_1 TSAQ.R57.esd Taenia solium cysticerca cDNA library Taenia solium cDNA, mRNA sequence		
EL762467_1 AD0186031 Taenia solium UNAM-cd1_adult Taenia solium cDNA, mRNA sequence		
**SJCHGC09631 protein**		
>gi|76156252|gb|AAX27472.2| SJCHGC09631 protein [Schistosoma japonicum]	found 1 time	267
EL746357_1 LV0308007 Taenia solium UNAM-cd2_larva Taenia solium cDNA, mRNA sequence		
>gi|350646584|emb|CCD58796.1| fer-1-related [Schistosoma mansoni]	found 1 time	506
EL747982_3 LV0332004 Taenia solium UNAM-cd2_larva Taenia solium cDNA, mRNA sequence		
**Sodium/glucose cotransporter**		
>gi|130489902|ref|NP_001076138.1| sodium/nucleoside cotransporter [Oryctolagus cuniculus]	found 1 time	431
EL757606_1 AD0035199 Taenia solium UNAM-cd1_adult Taenia solium cDNA, mRNA sequence		
>gi|405951099|gb|EKC19041.1| Sodium/glucose cotransporter 4 [Crassostrea gigas]	found 6 times	240
EL759523_3 AD0124037 Taenia solium UNAM-cd1_adult Taenia solium cDNA, mRNA sequence		
EL757099_1 AD0032111 Taenia solium UNAM-cd1_adult Taenia solium cDNA, mRNA sequence		
EL756752_1 AD0031025 Taenia solium UNAM-cd1_adult Taenia solium cDNA, mRNA sequence		
EL754284_1 AD0021074 Taenia solium UNAM-cd1_adult Taenia solium cDNA, mRNA sequence		
EL751374_6 AD0009112 Taenia solium UNAM-cd1_adult Taenia solium cDNA, mRNA sequence		
EL749969_5 AD0003111 Taenia solium UNAM-cd1_adult Taenia solium cDNA, mRNA sequence		
**T24**		
>gi|37786712|gb|AAP47268.1| T24 [Taenia solium]	found 16 times	226
GT892052_2 TSAK.R37.esd Taenia solium cysticerca cDNA library Taenia solium cDNA, mRNA sequence		
GT890179_3 TSBU.R58.esd Taenia solium cysticerca cDNA library Taenia solium cDNA, mRNA sequence		
EL747841_1 LV0330006 Taenia solium UNAM-cd2_larva Taenia solium cDNA, mRNA sequence		
EL747667_3 LV0327010 Taenia solium UNAM-cd2_larva Taenia solium cDNA, mRNA sequence		
EL747249_1 LV0320046 Taenia solium UNAM-cd2_larva Taenia solium cDNA, mRNA sequence		
EL746811_1 LV0314024 Taenia solium UNAM-cd2_larva Taenia solium cDNA, mRNA sequence		
EL746463_1 LV0309033 Taenia solium UNAM-cd2_larva Taenia solium cDNA, mRNA sequence		
EL746245_2 LV0305029 Taenia solium UNAM-cd2_larva Taenia solium cDNA, mRNA sequence		
EL744786_2 LV0280012 Taenia solium UNAM-cd2_larva Taenia solium cDNA, mRNA sequence		
EL743637_1 LV0260025 Taenia solium UNAM-cd2_larva Taenia solium cDNA, mRNA sequence		
EL743266_3 LV0254047 Taenia solium UNAM-cd2_larva Taenia solium cDNA, mRNA sequence		
EL742782_2 LV0246065 Taenia solium UNAM-cd2_larva Taenia solium cDNA, mRNA sequence		
EL742759_1 LV0246042 Taenia solium UNAM-cd2_larva Taenia solium cDNA, mRNA sequence		
EL741588_1 LV0228070 Taenia solium UNAM-cd2_larva Taenia solium cDNA, mRNA sequence		
EL741128_1 LV0221029 Taenia solium UNAM-cd2_larva Taenia solium cDNA, mRNA sequence		
EL740403_1 LV0208023 Taenia solium UNAM-cd2_larva Taenia solium cDNA, mRNA sequence		
**TSP1**		
>gi|209967595|gb|ACJ02402.1| TSP1 [Echinococcus multilocularis]	found 4 times	309
EL744430_1 LV0272045 Taenia solium UNAM-cd2_larva Taenia solium cDNA, mRNA sequence		
EL742816_1 LV0247034 Taenia solium UNAM-cd2_larva Taenia solium cDNA, mRNA sequence		

Table 1: Continued.

**Protein representing protein group**<br>>Protein identified by ESTs<br>EST	number of ESTs linked to protein	TBLASTN score
EL740770_2 LV0216004 Taenia solium UNAM-cd2_larva Taenia solium cDNA, mRNA sequence		
EL740643_1 LV0213025 Taenia solium UNAM-cd2_larva Taenia solium cDNA, mRNA sequence		
**Phosphoenolpyruvate carboxykinase**		
>gi|283466482|emb|CBH36496.1| phosphoenolpyruvate carboxykinase, partial [Taenia solium]	found 1 time	838
EL743308_2 LV0255022 Taenia solium UNAM-cd2_larva Taenia solium cDNA, mRNA sequence		
>gi|338827788|gb|AEJ15814.1| phosphoenolpyruvate carboxykinase [Echinococcus granulosus]	found 3 times	924
EL762926_3 AD0195046 Taenia solium UNAM-cd1_adult Taenia solium cDNA, mRNA sequence		
EL758250_3 AD0102061 Taenia solium UNAM-cd1_adult Taenia solium cDNA, mRNA sequence		
EL744294_1 LV0270041 Taenia solium UNAM-cd2_larva Taenia solium cDNA, mRNA sequence		
**Phosphoglucomutase-1**		
>gi|358337844|dbj|GAA56181.1| phosphoglucomutase-1 [Clonorchis sinensis]	found 2 times	424
GT891888_1 TSBY.R45.esd Taenia solium cysticerca cDNA library Taenia solium cDNA, mRNA sequence		
GT894047_1 TSAX.R47.esd Taenia solium cysticerca cDNA library Taenia solium cDNA, mRNA sequence		
**Phosphoglucose isomerase**		
>gi|154369446|gb|ABS81352.1| phosphoglucose isomerase [Echinococcus multilocularis]	found 3 times	592
EL761414_1 AD0163023 Taenia solium UNAM-cd1_adult Taenia solium cDNA, mRNA sequence		
EL758048_2 AD0099060 Taenia solium UNAM-cd1_adult Taenia solium cDNA, mRNA sequence		
EL742200_1 LV0238019 Taenia solium UNAM-cd2_larva Taenia solium cDNA, mRNA sequence		
**Family T2 unassigned peptidase**		
>gi|256088374|ref|XP_002580314.1| family T2 unassigned peptidase (T02 family) [Schistosoma mansoni]	found 4 times	244
EL753348_5 AD0017106 Taenia solium UNAM-cd1_adult Taenia solium cDNA, mRNA sequence		
EL753330_5 AD0017088 Taenia solium UNAM-cd1_adult Taenia solium cDNA, mRNA sequence		
EL752760_5 AD0015019 Taenia solium UNAM-cd1_adult Taenia solium cDNA, mRNA sequence		
EL751935_5 AD0011178 Taenia solium UNAM-cd1_adult Taenia solium cDNA, mRNA sequence		
**Trypsin-like protein**		
>gi|311335041|gb|ADP89566.1| trypsin-like protein [Taenia solium]	found 4 times	594
GT226944_1 tscaa0_001330.z1.scf Taenia solium adult full-length cDNA library Taenia solium cDNA clone tscaa0_001330.z1.scf 5', mRNA sequence		
EL748643_1 LV0343028 Taenia solium UNAM-cd2_larva Taenia solium cDNA, mRNA sequence		
EL743166_1 LV0253013 Taenia solium UNAM-cd2_larva Taenia solium cDNA, mRNA sequence		
EL741024_3 LV0219074 Taenia solium UNAM-cd2_larva Taenia solium cDNA, mRNA sequence		
**Hypothetical protein**		
>gi|21912540|emb|CAD21525.1| hypothetical protein [Taenia solium]	found 19 times	326
GT890827_1 TSAF.R79.esd Taenia solium cysticerca cDNA library Taenia solium cDNA, mRNA sequence		
GT890725_1 TSAY.R93.esd Taenia solium cysticerca cDNA library Taenia solium cDNA, mRNA sequence		
GT890255_1 TSBN.R46.esd Taenia solium cysticerca cDNA library Taenia solium cDNA, mRNA sequence		
GT284868_1 NDTC009-H06 Taenia saginata adult full-length cDNA library Taenia saginata cDNA clone NDTC009-H06 5', mRNA sequence		
EL759563_3 AD0125021 Taenia solium UNAM-cd1_adult Taenia solium cDNA, mRNA sequence		
EL755026_1 AD0024043 Taenia solium UNAM-cd1_adult Taenia solium cDNA, mRNA sequence		
EL753060_1 AD0016098 Taenia solium UNAM-cd1_adult Taenia solium cDNA, mRNA sequence		
EL751101_2 AD0008112 Taenia solium UNAM-cd1_adult Taenia solium cDNA, mRNA sequence		
EL748967_4 LV0349016 Taenia solium UNAM-cd2_larva Taenia solium cDNA, mRNA sequence		
EL748908_3 LV0348019 Taenia solium UNAM-cd2_larva Taenia solium cDNA, mRNA sequence		
EL748313_1 LV0338002 Taenia solium UNAM-cd2_larva Taenia solium cDNA, mRNA sequence		
EL748042_2 LV0332064 Taenia solium UNAM-cd2_larva Taenia solium cDNA, mRNA sequence		
EL747429_3 LV0323038 Taenia solium UNAM-cd2_larva Taenia solium cDNA, mRNA sequence		
EL746269_3 LV0306020 Taenia solium UNAM-cd2_larva Taenia solium cDNA, mRNA sequence		
EL744802_3 LV0280028 Taenia solium UNAM-cd2_larva Taenia solium cDNA, mRNA sequence		
EL742091_3 LV0236020 Taenia solium UNAM-cd2_larva Taenia solium cDNA, mRNA sequence		
EL741905_2 LV0233038 Taenia solium UNAM-cd2_larva Taenia solium cDNA, mRNA sequence		
EL741778_3 LV0231056 Taenia solium UNAM-cd2_larva Taenia solium cDNA, mRNA sequence		
EL740944_1 LV0218055 Taenia solium UNAM-cd2_larva Taenia solium cDNA, mRNA sequence		
**Cathepsin**		
>gi|325302580|dbj|BAJ83490.1| cathepsin B-like peptidase [Echinococcus multilocularis]	found 1 time	681
GT893175_2 TSCE.R69.esd Taenia solium cysticerca cDNA library Taenia solium cDNA, mRNA sequence		
>gi|226476878|emb|CAX72319.1| cathepsin D (lysosomal aspartyl protease) [Schistosoma japonicum]	found 1 time	203
EL761292_4 AD0161009 Taenia solium UNAM-cd1_adult Taenia solium cDNA, mRNA sequence		
>gi|336454164|gb|AEI58896.1| cathepsin D [Pinctada maxima]	found 1 time	292
EL745444_2 LV0291036 Taenia solium UNAM-cd2_larva Taenia solium cDNA, mRNA sequence		
**Kunitz protein 8**		
>gi|223037336|gb|ACM79010.1| Kunitz protein 8 [Echinococcus granulosus]	found 2 times	356
GT889523_3 TSBZ.R31.esd Taenia solium cysticerca cDNA library Taenia solium cDNA, mRNA sequence		
GT890420_2 TSCD.R67.esd Taenia solium cysticerca cDNA library Taenia solium cDNA, mRNA sequence		
>gi|405977066|gb|EKC41536.1| Papilin [Crassostrea gigas]	found 1 time	202
EL743081_3 LV0251046 Taenia solium UNAM-cd2_larva Taenia solium cDNA, mRNA sequence		
**Putative major vault protein**		
>gi|62178032|gb|AAX73176.1| putative MVP protein [Echinococcus granulosus]	found 2 times	563
GT227141_2 tscaa0_002678.z1.scf Taenia solium adult full-length cDNA library Taenia solium cDNA clone tscaa0_002678.z1.scf 5', mRNA sequence		
EL741525_1 LV0228007 Taenia solium UNAM-cd2_larva Taenia solium cDNA, mRNA sequence		
**SJCHGC02435 protein**		

Table 1: Continued.

<table>
<tr><th>Protein representing protein group
>Protein identified by ESTs
EST</th><th>number of ESTs linked to protein</th><th>TBLASTN score</th></tr>
<tr><td>>gi|56756018|gb|AAW26187.1| SJCHGC02435 protein [Schistosoma japonicum]</td><td>found 5 times</td><td>248</td></tr>
<tr><td>EL758001_2 AD0099013 Taenia solium UNAM-cd1_adult Taenia solium cDNA, mRNA sequence</td><td></td><td></td></tr>
<tr><td>EL749217_1 LV0355031 Taenia solium UNAM-cd2_larva Taenia solium cDNA, mRNA sequence</td><td></td><td></td></tr>
<tr><td>EL748701_3 LV0344024 Taenia solium UNAM-cd2_larva Taenia solium cDNA, mRNA sequence</td><td></td><td></td></tr>
<tr><td>EL748450_2 LV0340024 Taenia solium UNAM-cd2_larva Taenia solium cDNA, mRNA sequence</td><td></td><td></td></tr>
<tr><td>EL745962_2 LV0299051 Taenia solium UNAM-cd2_larva Taenia solium cDNA, mRNA sequence</td><td></td><td></td></tr>
<tr><td>Sodium/potassium-transporting ATPase</td><td></td><td></td></tr>
<tr><td>>gi|124784584|gb|ABN14984.1| sodium/potassium-transporting ATPase beta nervous system antigen 1 [Taenia asiatica]</td><td>found 1 time</td><td>446</td></tr>
<tr><td>EL741140_2 LV0221041 Taenia solium UNAM-cd2_larva Taenia solium cDNA, mRNA sequence</td><td></td><td></td></tr>
<tr><td>>gi|74794482|sp|Q6RWA9.1|AT1A_TAESO RecName: Full=Sodium/potassium-transporting ATPase subunit alpha;</td><td>found 1 time</td><td>2114</td></tr>
<tr><td>EL748309_1 LV0337030 Taenia solium UNAM-cd2_larva Taenia solium cDNA, mRNA sequence</td><td></td><td></td></tr>
<tr><td>Four and a half LIM domains protein 3</td><td></td><td></td></tr>
<tr><td>>gi|358341124|dbj|GAA48877.1| four and a half LIM domains protein 3 [Clonorchis sinensis]</td><td>found 7 times</td><td>283</td></tr>
<tr><td>GT891759_3 TSBI.R46.esd Taenia solium cysticerca cDNA library Taenia solium cDNA, mRNA sequence</td><td></td><td></td></tr>
<tr><td>GT891463_1 TSAJ.R8.esd Taenia solium cysticerca cDNA library Taenia solium cDNA, mRNA sequence</td><td></td><td></td></tr>
<tr><td>GT891328_1 TSAE.R45.esd Taenia solium cysticerca cDNA library Taenia solium cDNA, mRNA sequence</td><td></td><td></td></tr>
<tr><td>GT890904_1 TSAT.R31.esd Taenia solium cysticerca cDNA library Taenia solium cDNA, mRNA sequence</td><td></td><td></td></tr>
<tr><td>GT893900_3 TSAH.R24.esd Taenia solium cysticerca cDNA library Taenia solium cDNA, mRNA sequence</td><td></td><td></td></tr>
<tr><td>GT892683_3 TSBY.R78.esd Taenia solium cysticerca cDNA library Taenia solium cDNA, mRNA sequence</td><td></td><td></td></tr>
<tr><td>EL757588_3 AD0035181 Taenia solium UNAM-cd1_adult Taenia solium cDNA, mRNA sequence</td><td></td><td></td></tr>
<tr><td>Immunogenic protein</td><td></td><td></td></tr>
<tr><td>>gi|4803715|emb|CAB42638.1| immunogenic protein [Taenia solium]</td><td>found 72 times</td><td>141</td></tr>
<tr><td>EL747788_2 LV0329026 Taenia solium UNAM-cd2_larva Taenia solium cDNA, mRNA sequence</td><td></td><td></td></tr>
<tr><td>EL747764_3 LV0329002 Taenia solium UNAM-cd2_larva Taenia solium cDNA, mRNA sequence</td><td></td><td></td></tr>
<tr><td>EL747757_1 LV0328045 Taenia solium UNAM-cd2_larva Taenia solium cDNA, mRNA sequence</td><td></td><td></td></tr>
<tr><td>EL747690_1 LV0327033 Taenia solium UNAM-cd2_larva Taenia solium cDNA, mRNA sequence</td><td></td><td></td></tr>
<tr><td>EL747533_3 LV0325012 Taenia solium UNAM-cd2_larva Taenia solium cDNA, mRNA sequence</td><td></td><td></td></tr>
<tr><td>EL747481_1 LV0324017 Taenia solium UNAM-cd2_larva Taenia solium cDNA, mRNA sequence</td><td></td><td></td></tr>
<tr><td>EL747413_3 LV0323022 Taenia solium UNAM-cd2_larva Taenia solium cDNA, mRNA sequence</td><td></td><td></td></tr>
<tr><td>EL747358_3 LV0322035 Taenia solium UNAM-cd2_larva Taenia solium cDNA, mRNA sequence</td><td></td><td></td></tr>
<tr><td>EL747197_2 LV0319056 Taenia solium UNAM-cd2_larva Taenia solium cDNA, mRNA sequence</td><td></td><td></td></tr>
<tr><td>EL747037_2 LV0317034 Taenia solium UNAM-cd2_larva Taenia solium cDNA, mRNA sequence</td><td></td><td></td></tr>
<tr><td>EL746883_1 LV0315025 Taenia solium UNAM-cd2_larva Taenia solium cDNA, mRNA sequence</td><td></td><td></td></tr>
<tr><td>EL746838_1 LV0314051 Taenia solium UNAM-cd2_larva Taenia solium cDNA, mRNA sequence</td><td></td><td></td></tr>
<tr><td>EL746722_1 LV0313005 Taenia solium UNAM-cd2_larva Taenia solium cDNA, mRNA sequence</td><td></td><td></td></tr>
<tr><td>EL746698_3 LV0312059 Taenia solium UNAM-cd2_larva Taenia solium cDNA, mRNA sequence</td><td></td><td></td></tr>
<tr><td>EL746685_2 LV0312046 Taenia solium UNAM-cd2_larva Taenia solium cDNA, mRNA sequence</td><td></td><td></td></tr>
<tr><td>EL746645_1 LV0312006 Taenia solium UNAM-cd2_larva Taenia solium cDNA, mRNA sequence</td><td></td><td></td></tr>
<tr><td>EL746626_1 LV0311055 Taenia solium UNAM-cd2_larva Taenia solium cDNA, mRNA sequence</td><td></td><td></td></tr>
<tr><td>EL746464_3 LV0309034 Taenia solium UNAM-cd2_larva Taenia solium cDNA, mRNA sequence</td><td></td><td></td></tr>
<tr><td>EL746350_2 LV0307064 Taenia solium UNAM-cd2_larva Taenia solium cDNA, mRNA sequence</td><td></td><td></td></tr>
<tr><td>EL746176_6 LV0304001 Taenia solium UNAM-cd2_larva Taenia solium cDNA, mRNA sequence</td><td></td><td></td></tr>
<tr><td>EL745928_3 LV0299017 Taenia solium UNAM-cd2_larva Taenia solium cDNA, mRNA sequence</td><td></td><td></td></tr>
<tr><td>EL745713_1 LV0296003 Taenia solium UNAM-cd2_larva Taenia solium cDNA, mRNA sequence</td><td></td><td></td></tr>
<tr><td>EL745541_3 LV0293019 Taenia solium UNAM-cd2_larva Taenia solium cDNA, mRNA sequence</td><td></td><td></td></tr>
<tr><td>EL745242_1 LV0288024 Taenia solium UNAM-cd2_larva Taenia solium cDNA, mRNA sequence</td><td></td><td></td></tr>
<tr><td>EL744793_1 LV0280019 Taenia solium UNAM-cd2_larva Taenia solium cDNA, mRNA sequence</td><td></td><td></td></tr>
<tr><td>EL744790_1 LV0280016 Taenia solium UNAM-cd2_larva Taenia solium cDNA, mRNA sequence</td><td></td><td></td></tr>
<tr><td>EL744770_2 LV0279039 Taenia solium UNAM-cd2_larva Taenia solium cDNA, mRNA sequence</td><td></td><td></td></tr>
<tr><td>EL744722_3 LV0278041 Taenia solium UNAM-cd2_larva Taenia solium cDNA, mRNA sequence</td><td></td><td></td></tr>
<tr><td>EL744667_1 LV0277044 Taenia solium UNAM-cd2_larva Taenia solium cDNA, mRNA sequence</td><td></td><td></td></tr>
<tr><td>EL744622_3 LV0276040 Taenia solium UNAM-cd2_larva Taenia solium cDNA, mRNA sequence</td><td></td><td></td></tr>
<tr><td>EL744551_2 LV0275026 Taenia solium UNAM-cd2_larva Taenia solium cDNA, mRNA sequence</td><td></td><td></td></tr>
<tr><td>EL744550_1 LV0275025 Taenia solium UNAM-cd2_larva Taenia solium cDNA, mRNA sequence</td><td></td><td></td></tr>
<tr><td>EL744525_5 LV0274028 Taenia solium UNAM-cd2_larva Taenia solium cDNA, mRNA sequence</td><td></td><td></td></tr>
<tr><td>EL744225_1 LV0269036 Taenia solium UNAM-cd2_larva Taenia solium cDNA, mRNA sequence</td><td></td><td></td></tr>
<tr><td>EL744041_2 LV0266041 Taenia solium UNAM-cd2_larva Taenia solium cDNA, mRNA sequence</td><td></td><td></td></tr>
<tr><td>EL743840_3 LV0263009 Taenia solium UNAM-cd2_larva Taenia solium cDNA, mRNA sequence</td><td></td><td></td></tr>
<tr><td>EL743738_3 LV0261047 Taenia solium UNAM-cd2_larva Taenia solium cDNA, mRNA sequence</td><td></td><td></td></tr>
<tr><td>EL743722_3 LV0261031 Taenia solium UNAM-cd2_larva Taenia solium cDNA, mRNA sequence</td><td></td><td></td></tr>
<tr><td>EL743636_2 LV0260024 Taenia solium UNAM-cd2_larva Taenia solium cDNA, mRNA sequence</td><td></td><td></td></tr>
<tr><td>EL743383_1 LV0256044 Taenia solium UNAM-cd2_larva Taenia solium cDNA, mRNA sequence</td><td></td><td></td></tr>
<tr><td>EL743280_3 LV0254061 Taenia solium UNAM-cd2_larva Taenia solium cDNA, mRNA sequence</td><td></td><td></td></tr>
<tr><td>EL743251_2 LV0254032 Taenia solium UNAM-cd2_larva Taenia solium cDNA, mRNA sequence</td><td></td><td></td></tr>
<tr><td>EL743207_3 LV0253054 Taenia solium UNAM-cd2_larva Taenia solium cDNA, mRNA sequence</td><td></td><td></td></tr>
<tr><td>EL742907_2 LV0249022 Taenia solium UNAM-cd2_larva Taenia solium cDNA, mRNA sequence</td><td></td><td></td></tr>
<tr><td>EL742882_2 LV0248044 Taenia solium UNAM-cd2_larva Taenia solium cDNA, mRNA sequence</td><td></td><td></td></tr>
<tr><td>EL742767_3 LV0246050 Taenia solium UNAM-cd2_larva Taenia solium cDNA, mRNA sequence</td><td></td><td></td></tr>
<tr><td>EL742691_2 LV0245046 Taenia solium UNAM-cd2_larva Taenia solium cDNA, mRNA sequence</td><td></td><td></td></tr>
<tr><td>EL742617_1 LV0244048 Taenia solium UNAM-cd2_larva Taenia solium cDNA, mRNA sequence</td><td></td><td></td></tr>
<tr><td>EL742582_2 LV0244013 Taenia solium UNAM-cd2_larva Taenia solium cDNA, mRNA sequence</td><td></td><td></td></tr>
<tr><td>EL742435_3 LV0241061 Taenia solium UNAM-cd2_larva Taenia solium cDNA, mRNA sequence</td><td></td><td></td></tr>
<tr><td>EL742316_3 LV0240011 Taenia solium UNAM-cd2_larva Taenia solium cDNA, mRNA sequence</td><td></td><td></td></tr>
<tr><td>EL742282_2 LV0239034 Taenia solium UNAM-cd2_larva Taenia solium cDNA, mRNA sequence</td><td></td><td></td></tr>
<tr><td>EL742179_1 LV0237067 Taenia solium UNAM-cd2_larva Taenia solium cDNA, mRNA sequence</td><td></td><td></td></tr>
<tr><td>EL742178_1 LV0237066 Taenia solium UNAM-cd2_larva Taenia solium cDNA, mRNA sequence</td><td></td><td></td></tr>
<tr><td>EL742032_3 LV0235021 Taenia solium UNAM-cd2_larva Taenia solium cDNA, mRNA sequence</td><td></td><td></td></tr>
<tr><td>EL741952_2 LV0234013 Taenia solium UNAM-cd2_larva Taenia solium cDNA, mRNA sequence</td><td></td><td></td></tr>
<tr><td>EL741923_3 LV0233056 Taenia solium UNAM-cd2_larva Taenia solium cDNA, mRNA sequence</td><td></td><td></td></tr>
</table>

Table 1: Continued.

Protein representing protein group<br>>Protein identified by ESTs<br>EST	number of ESTs linked to protein	TBLASTN score
EL741817_3 LV0232022 Taenia solium UNAM-cd2_larva Taenia solium cDNA, mRNA sequence		
EL741555_1 LV0228037 Taenia solium UNAM-cd2_larva Taenia solium cDNA, mRNA sequence		
EL741451_3 LV0227007 Taenia solium UNAM-cd2_larva Taenia solium cDNA, mRNA sequence		
EL741428_3 LV0226047 Taenia solium UNAM-cd2_larva Taenia solium cDNA, mRNA sequence		
EL741340_3 LV0224060 Taenia solium UNAM-cd2_larva Taenia solium cDNA, mRNA sequence		
EL741252_3 LV0223030 Taenia solium UNAM-cd2_larva Taenia solium cDNA, mRNA sequence		
EL741190_3 LV0222023 Taenia solium UNAM-cd2_larva Taenia solium cDNA, mRNA sequence		
EL741112_1 LV0221013 Taenia solium UNAM-cd2_larva Taenia solium cDNA, mRNA sequence		
EL741102_3 LV0221003 Taenia solium UNAM-cd2_larva Taenia solium cDNA, mRNA sequence		
EL740923_1 LV0218034 Taenia solium UNAM-cd2_larva Taenia solium cDNA, mRNA sequence		
EL740891_3 LV0218002 Taenia solium UNAM-cd2_larva Taenia solium cDNA, mRNA sequence		
EL740818_3 LV0217001 Taenia solium UNAM-cd2_larva Taenia solium cDNA, mRNA sequence		
EL740773_3 LV0216007 Taenia solium UNAM-cd2_larva Taenia solium cDNA, mRNA sequence		
EL740471_2 LV0209049 Taenia solium UNAM-cd2_larva Taenia solium cDNA, mRNA sequence		
EL740282_2 LV0206014 Taenia solium UNAM-cd2_larva Taenia solium cDNA, mRNA sequence		
>gi|45181628|gb|AAS55469.1| Cysticercus cellulosae-specific antigenic polypeptide [Taenia solium]	found 17 times	143
EL748630_3 LV0343015 Taenia solium UNAM-cd2_larva Taenia solium cDNA, mRNA sequence		
EL748084_3 LV0333035 Taenia solium UNAM-cd2_larva Taenia solium cDNA, mRNA sequence		
EL747923_1 LV0331016 Taenia solium UNAM-cd2_larva Taenia solium cDNA, mRNA sequence		
EL747819_5 LV0329057 Taenia solium UNAM-cd2_larva Taenia solium cDNA, mRNA sequence		
EL746703_3 LV0312064 Taenia solium UNAM-cd2_larva Taenia solium cDNA, mRNA sequence		
EL746054_3 LV0301009 Taenia solium UNAM-cd2_larva Taenia solium cDNA, mRNA sequence		
EL745579_3 LV0293057 Taenia solium UNAM-cd2_larva Taenia solium cDNA, mRNA sequence		
EL744936_2 LV0282062 Taenia solium UNAM-cd2_larva Taenia solium cDNA, mRNA sequence		
EL744151_2 LV0268018 Taenia solium UNAM-cd2_larva Taenia solium cDNA, mRNA sequence		
EL743742_1 LV0261051 Taenia solium UNAM-cd2_larva Taenia solium cDNA, mRNA sequence		
EL743564_2 LV0259027 Taenia solium UNAM-cd2_larva Taenia solium cDNA, mRNA sequence		
EL742865_3 LV0248027 Taenia solium UNAM-cd2_larva Taenia solium cDNA, mRNA sequence		
EL742687_1 LV0245042 Taenia solium UNAM-cd2_larva Taenia solium cDNA, mRNA sequence		
EL742234_2 LV0238053 Taenia solium UNAM-cd2_larva Taenia solium cDNA, mRNA sequence		
EL741113_3 LV0221014 Taenia solium UNAM-cd2_larva Taenia solium cDNA, mRNA sequence		
EL741091_1 LV0220064 Taenia solium UNAM-cd2_larva Taenia solium cDNA, mRNA sequence		
EL740873_2 LV0217056 Taenia solium UNAM-cd2_larva Taenia solium cDNA, mRNA sequence		
