# Supplementary material for: Use of expressed sequence tags as an alternative approach for the identification of Taenia solium metacestode excretion/secretion proteins
Source: BMC Res Notes. 2013 Jun 6;6:224. doi: 10.1186/1756-0500-6-224 (PMC3686625; doi:10.1186/1756-0500-6-224)
Supplement: Additional file 2 — Protein groups (n = 106) identified in Taenia solium metacestode excretion/secretion proteins, organized by Gene Ontology annotation information on biological process and molecular function. Groups marked with an asterisk have been identified in the previous analysis as well. For simplicity, all protein groups are represented by one protein. [file 1756-0500-6-224-S2.pdf]

Table 1: Protein groups ($n = 106$) identified in *T. solium* metacestode ESPs, organized by Gene Ontology annotation information on biological process and molecular function. Groups marked with an asterisk have been identified in the previous analysis as well[1]. For simplicity, all protein groups are represented by one protein.

**Gene Ontology classification** Protein group	Closest organism	gi code	Proteins[a)]	ESTs[b)]
**1) No Gene Ontology information**				
Major egg antigen	*Clonorchis sinensis*	358336515	1	2
8 kDa protein family*	*Taenia solium*	-	12	511
ES1 protein homolog	Multiple[c)]	-	3	5
Phosphoglyceride transfer protein	*Taenia asiatica*	124782980	1	7
Alpha-2-macroglobulin-like protein 1	*Clonorchis sinensis*	358333571	1	5
Hypothetical protein*	*Taenia solium*	21726976	1	2
Diagnostic antigen GP50*	*Taenia solium*	-	2	11
Aldose 1-epimerase	*Clonorchis sinensis*	358334888	1	2
SJCHGC02626 protein	*Schistosoma japonicum*	-	3	7
Hypothetical protein	*Schistosoma mansoni*	256079415	1	4
T24*	*Taenia solium*	37786712	1	16
TSP1	*Echinococcus multilocularis*	209967595	1	4
Hypothetical protein*	*Taenia solium*	21912540	1	19
Immunogenic protein*	*Taenia solium*	-	2	89
Putative major vault protein	*Echinococcus granulosus*	62178032	1	2
**2) Binding (miscellaneous)**				
Actin-binding and severin family * group-like protein	Multiple	-	4	60
H17g protein, tegumental antigen*	*Taenia* spp.	-	3	11
14-3-3 protein*	Multiple	-	8	26
Peptidyl-prolyl cis-trans isomerase*	Multiple	-	5	25
Calcium binding protein 39*	Multiple	-	2	2
Filamin	Multiple	-	3	7
Methionyl-tRNA synthetase cytoplasmic	*Clonorchis sinensis*	358255967	1	5
SJCHGC09631 protein	*Schistosoma* spp.	-	2	2
four and a half LIM domains protein 3	*Clonorchis sinensis*	358341124	1	7
Annexin*	Multiple	-	5	12
Alpha-actinin isoform B	*Taenia asiatica*	124783372	1	3
Calumenin	*Taenia asiatica*	124784033	1	2
Calcium-binding protein	*Schistosoma mansoni*	256071353	1	2
Lysyl oxidase-like	*Schistosoma mansoni*	256072781	1	2
Nucleoside diphosphate kinase B-like*	*Metaseiulus occidentalis*	391344390	1	3
Porphobilinogen synthase	Multiple	-	3	3
Phosphoglucomutase-1	*Clonorchis sinensis*	358337844	1	2
Fibrillar collagen	Multiple	-	9	16
Apolipoprotein AI binding protein*	*Echinococcus multilocularis*	223640019	1	2
Small GTPase RhoA*	Multiple	-	11	19
**3) Gluconeogenesis/Glycolysis/Metabolic processes (miscellaneous)**				
Glutathione S-transferase*	*Taenia solium*	-	3	26
Adenylosuccinate synthetase	*Schistosoma mansoni*	387912858	1	4
Adenylate kinase	Multiple	-	2	4
UDP-glucose pyrophosphorylase 2	*Schistosoma* spp.	-	2	2
Hypothetical protein SINV_09109	*Solenopsis invicta*	322793762	1	2
Aspartate aminotransferase	Multiple	-	3	5
Lactate dehydrogenase A	*Taenia solium*	318054471	1	6
SJCHGC05968 protein	Multiple	-	2	2
Methylthioadenosine phosphorylase	Multiple	-	2	2
Glycogen phosphorylase muscle form*	Multiple	-	2	7
Ornithine aminotransferase	Multiple	-	3	3
Endoglycoceramidase	Multiple	-	3	3
Carbonic anhydrase*	Multiple	-	3	5

Table 1: Continued.

**Gene Ontology classification** Protein group	Closest organism	gi code	Proteins[a)]	ESTs[b)]
Transketolase*	*Echinococcus multilocularis*	27526313	1	5
Aminoacylase	Multiple[c)]	-	2	2
Glucose-6-phosphate 1-dehydrogenase-like	*Sus scrofa*	350595984	1	2
Triosephosphate isomerase*	Multiple	-	2	2
Enolase*	Multiple	-	3	5
Phosphoenolpyruvate carboxykinase*	Multiple	-	2	4
Phosphoglucose isomerase*	*Echinococcus multilocularis*	154369446	1	3
Glyceraldehyde-3-phosphate dehydrogenase*	Multiple	-	3	14
Phosphoglycerate kinase*	Multiple	-	5	5
Phosphoglycerate mutase	Multiple	-	3	7
Fructose-bisphosphate aldolase*	*Echinococcus granulosus*	29336561	1	9
**4) (Endo)peptidase activity**				
Calpain	Multiple	-	5	5
UDP-glucose 4-epimerase	Multiple	-	2	2
Proteasome subunits*	Multiple	-	10	19
Cathepsin*	Multiple	-	3	3
Trypsin-like protein*	*Taenia solium*	311335041	1	4
Dipeptidyl-peptidase	Multiple	-	2	3
Glutamate carboxypeptidase 2	*Clonorchis sinensis*	358331956	1	3
**5) Endopeptidase inhibitor activity**				
Leukocyte elastase inhibitor*	Multiple	-	2	2
Kunitz protein 8	Multiple	-	2	3
Immunogenic protein Ts11*	*Taenia solium*	7339849	1	3
**6) Cell redox homeostasis/Oxidation-reduction related**				
Thioredoxin*	Multiple	-	5	23
Peroxiredoxin*	Multiple	-	6	31
Aldo-keto reductase*	Multiple	-	4	25
Carbonyl reductase	*Schistosoma* spp.	-	3	3
Cu/Zn Superoxide dismutase*	*Taenia solium*	347948498	1	2
Methionine sulfoxide reductase	Multiple	-	2	4
procollagen-lysine, 2-oxoglutarate 5-dioxygenase 3	Multiple	-	3	6
**7) Transport**				
Ferritin*	*Taenia saginata*	1297064	1	15
Charged multivesicular body protein	Multiple	-	3	4
SJCHGC06082 protein	Multiple	-	2	8
Glycolipid transfer protein-like protein	*Taenia asiatica*	124782916	1	2
Gamma-soluble NSF attachment protein	Multiple	-	4	10
Cytosolic fatty acid binding protein*	*Taenia solium*	82412213	1	352
Sodium/glucose cotransporter	Multiple	-	2	7
Sodium/potassium-transporting ATPase*	*Taenia* spp.	-	2	2
**8) Motor activity/Cytoskeleton and Microtubule related**				
Tubulin polymerization-promoting protein	Multiple	-	2	2
Tubulin*	Multiple	-	13	27
Dynein light chain*	Multiple	-	9	42
Tegumental protein*	*Echinococcus granulosus*	60459970	1	37
Paramyosin*	Multiple	-	2	2
Myophilin	Multiple	-	2	19
Actin*	Multiple	-	6	10

Table 1: Continued.

**Gene Ontology classification**<br>Protein group	Closest organism	gi code	Proteins[a)]	ESTs[b)]
**9) Translational elongation/initiation**				
Elongation factors*	Multiple[c)]	-	6	7
Translation initiation factor 5A	Multiple	-	2	4
**10) Ubiquitin-protein ligase activity**				
Ubiquitin*	Multiple	-	6	10
Ubiquitin-conjugating enzyme	Multiple	-	4	10
**11) Response to unfolded protein**				
Heat shock protein 90*	Multiple	-	4	7
Heat shock protein 70*	Multiple	-	7	13
**12) Methylation**				
Protein-l-isoaspartate o-methyltransferase	*Schistosoma mansoni*	256081696	1	2
Adenosylhomocysteinase*	Multiple	-	3	4
**13) Miscellaneous**				
Protein DJ-1-like	Multiple	-	2	4
6-phosphogluconolactonase	Multiple	-	2	4
SJCHGC02435 protein	*Schistosoma japonicum*	56756018	1	5
Family T2 unassigned peptidase	*Schistosoma mansoni*	256088374	1	4
3'(2'), 5'-bisphosphate nucleotidase	Multiple	-	2	2
RAB GDP dissociation inhibitor alpha	Multiple	-	2	3
Laminin	Multiple	-	2	2

∗ Identified in the previous study as well.
a) The number of proteins in each protein group.
b) The number of ESTs that were matches to proteins in this protein group.
c) 'Multiple' indicates that different (helminth) genera have identified proteins in that protein group.

[1] Victor B, Kanobana K, Gabriël S, Polman K, Deckers N, Dorny P, Deelder AM, Palmblad M: **Proteomic analysis of *Taenia solium* metacestode excretion-secretion proteins**. *Proteomics* 2012, **12**(11):1860–1869.
